# Supplementary material for: Systematic selection between age and household structure for models aimed at emerging epidemic predictions
Source: Nat Commun. 2020 Feb 14;11:906. doi: 10.1038/s41467-019-14229-4 (PMC7021781; doi:10.1038/s41467-019-14229-4)
Supplement: Supplementary file 1 — Supplementary Information [file 41467_2019_14229_MOESM1_ESM.pdf]

Supplementary information

Pellis *et al.*

Systematic selection between age and household structure for models aimed at emerging epidemic predictions

Contents

1 Supplementary Methods **3**
1.1 General approach . . . 3
1.1.1 Model mapping procedure . . . 3
1.1.2 Temporal details of the infectivity profile . . . 4
1.1.3 Model mapping at fixed R_0 . . . 4
1.1.4 Contact matrices and mixing . . . 5
1.1.5 Transmission: next-generation matrices . . . 6
1.2 Age- and household-stratified model . . . 7
1.2.1 Population structure . . . 7
1.2.2 Global infectivity . . . 7
1.2.3 Within-household infectivity . . . 8
1.2.4 Computation of R_0 . . . 9
1.2.5 Incidence ratio of adults versus children in each generation . . . 11
1.2.6 Household secondary attack rate . . . 12
1.2.7 Final size . . . 15
1.2.8 Peak incidence and time to the peak . . . 16
1.2.9 Monte-Carlo computation of the real-time growth rate . . . 16
1.3 Age-stratified model . . . 18
1.4 Households model . . . 20
1.5 Individual-based stochastic simulation . . . 24
1.6 Data and parameter values . . . 26
1.6.1 Population structure of Great Britain . . . 26
1.6.2 Other population structures . . . 26
1.6.3 Contact patterns for the United Kingdom . . . 27
1.6.4 Summary of input parameter values . . . 28
1.6.5 Parameter values for infections explored in the main text . . . 28

2 Supplementary Discussion **29**
2.1 Numerical results with random mixing . . . 29
2.1.1 Early epidemic indicators and mapped parameters . . . 29
2.1.2 Predicted outputs . . . 30
2.1.3 Other rejection thresholds . . . 33
2.2 Numerical results with realistic UK contact patterns . . . 33
2.2.1 Early epidemic indicators and mapped parameters . . . 33
2.2.2 Predicted outputs . . . 34
2.3 Further sensitivity analysis . . . 34
2.3.1 Intermediate contact patterns . . . 34
2.3.2 Accuracy of model mapping: fixed R_0 versus fixed r . . . 35
2.3.3 Children less susceptible and/or infectious than adults . . . 35
2.3.4 Other populations . . . 36
2.4 Rule of thumb . . . 37

3 Supplementary Tables **40**

4 Supplementary Figures **45**

Supplementary References **69**

1 Supplementary Methods

1.1 General approach

1.1.1 Model mapping procedure

This study is fundamentally a work of model comparison, and comparing structurally different models, with parameters differing in number and biological meaning, is not a straightforward process. For a fair comparison, it is necessary to choose:

1. What is kept fixed across models: given different choices are possible (House and Keeling, 2008; Ajelli et al., 2010) we argue that for a fair comparison such quantities should be observable or directly estimable and with a model-independent biological interpretation; referred to in this study as *observables*, they are therefore likely to be some aggregate macro-parameters, like R_0 or the real-time growth rate;

2. How the other *basic model parameters*, which are likely not measured directly and whose definition is model-specific (e.g. the transmission rates in different environments) are derived from these observables: this process is likely to unravel numerous unidentifiability issues that should be made as clear as possible; and

3. How the models are compared in terms of their predictions, which we refer to as *model outputs*; like the observables, they are aggregate quantities that typically depend in a complex fashion on the basic model parameters.

The conclusions may fundamentally depend on such choices, so they need to be spelt out clearly and how results depend on them should be investigated. We recognise many practical contexts are likely to be significantly more complex than the one considered here, and parameter spaces are less likely to be explored so thoroughly, but we still advocate that this general approach be followed, choices made explicit and issues encountered discussed openly.

Here we assume we are observing the early phase of an outbreak of a new infection. By "early" we formally mean during the time window that occurs after the epidemic has taken off (i.e. when the initial phase, dominated by random events and possible stochastic extinction, is over and the initial conditions have been forgotten) but while the global depletion of susceptibles is still negligible. We refer to this time window as the *stable exponential growth window*. The observables, which are aggregate early indicators of how the epidemic is progressing, are mathematically well-defined during this stable exponential growth window.

We start by considering a (reasonably) complex model – an age- and household-stratified one, denoted by AH – and we assume it to be the "truth", i.e. a perfect representation of reality. For any arbitrary choice of the basic model parameters for model AH, all quantities of interest are computed, including the observables we want to keep fixed between all models in which they can be defined (in this study, R_0, the household secondary attack rate and the adults-to-children incidence ratio in each generation) and the outputs we want to compare between models (in this study, final size, peak daily incidence and time to incidence peak). We assume a sufficiently large population (ideally infinite), so that despite the models being stochastic we can invoke the law of large numbers to assume zero variance and work with average quantities only. In other words, we neglect any stochastic variability and assume a perfect deterministic relationship between the basic model parameters and the other model quantities.

The "true" model AH is then mapped onto structurally simpler ones. The term *map* is used to imply that the basic model parameters are not estimated from the observables using a statistical procedure. Instead, they are computed deterministically by inverting the function that would give the observable in terms of the basic model parameters (in most cases, when dealing with scalar functions, this is achieved using the `fzero` routine in Matlab; in more dimensions, the `fsolve` routine is used). Therefore, in

addition to lack of stochastic variation, we assume perfect knowledge of the observable, i.e. no measurement error, no noise and no other forms of bias in the observation. This approach has the limitation that some parts of the mapping procedure might in reality be hard to perform because of excessively wide confidence intervals in any practical statistical estimation procedure, but has the benefit of making the relationships and dependencies between all model quantities totally transparent, thus uncovering possible non-identifiability issues, which could instead be obscured by a complex statistical technique, and highlighting which pieces of information can and should be used to solve such issues even when advanced statistical tools are used.

1.1.2 Temporal details of the infectivity profile

We assume that individuals make infectious contacts at the points of a Poisson process with rate that changes over time; such a rate is called the *infectivity profile*. Infectious contacts lead to an infection if and only if, when contacted, an individual is susceptible. We assume for simplicity that the infectivity profile has the same shape, as a function of time, for all individuals in all environments (i.e. within-household and in the community), and that therefore the only difference occurs in a scaling factor. The normalised infectivity profile is called the *infectious contact interval distribution* (Kenah et al., 2008; Kenah, 2011) or, sometimes, the *generation time distribution* (Svensson, 2007).

Specifically, we choose a infectious contact interval distribution typical of influenza: a Gamma distribution with mean $T_{\mathrm{G}} = 2.85$ days (sometimes referred to as the *generation time*) and shape parameter $\alpha = 9$, in line with Wallinga and Lipsitch (2007), Fraser (2007) and as used by Pellis et al. (2010). Note that this choice has no impact on the mapping procedure, as explained in the next section: however, it affects other quantities, such as the real-time growth rate r, and some model outputs like the peak incidence and the time of the epidemic peak. Therefore, in order to quantify the mean number of generations to the peak and allow simpler generalisation of results to other infections, the time to the peak is rescaled by the generation time T_{G}.

Although some studies highlighted the presence of detectable differences in the generation time of individuals in different age classes in different environments (e.g. children at school versus adults in the community; see Cauchemez et al., 2011), the choice of using a single infectious contact interval distribution everywhere is a very convenient one, and is very commonly adopted (Ball et al., 2016; Trapman et al., 2016). The main reason is that the next-generation matrix approach and the real-time one (see Pellis et al., 2008, for details and Diekmann et al., 2012, p. 30-31 and 212-219, for a comparison between the two approaches) become equivalent for models with no households and, when households are included, are still quantitatively strongly related (Ball et al., 2016; Trapman et al., 2016).

1.1.3 Model mapping at fixed R_0

Some epidemic quantities, like the real-time growth rate (r), the peak incidence or the time to the peak, depend on the temporal details of the individuals' infectivity profiles; therefore, they are in general difficult to handle analytically. Some other quantities, notably the basic reproduction number R_0 (see Diekmann et al., 2012, Section 7, for the basic definition in homogeneously mixing and multitype models, and Pellis et al., 2012, and Ball et al., 2016, for its extension to models with households) and the epidemic final size distributions in either a small group or in the population at large, are time-integrated quantities: they depend on the individuals' infectivity profiles only through their total infectivity, i.e. the area under the infectivity profile curve. For this reason, they are often more easily tractable (see Pellis et al., 2008, or Ball et al., 2016) and available analytical results allow their efficient calculation to machine precision.

In order to make extensive model comparison feasible, we choose to base the mapping procedure only on time-integrated quantities: specifically, R_0, the household secondary attack rate and the incidence ratio of adults versus children in each generation. Once the parameters of each model are chosen or mapped, we use individual-based stochastic simulations to plot those outputs that depend on the temporal details of the individuals' infectivity profiles.

Because of our assumption that the infectious contact interval distribution is the same for all types in all environments, keeping fixed R_0 in the model mapping procedure is quantitatively very similar to keeping fixed the real-time growth rate r, which is an even more intuitive and readily available quantity early on in an epidemic (Trapman et al., 2016). The discrepancies between keeping fixed R_0 and r are due to two factors:

i) infectives make repeated infectious contacts towards each single susceptible household member, and the infection is transmitted at the time of the first contact (if any at all), thus occurring on average after a time spell shorter than T_G in general (see Pellis et al., 2015, for a careful explanation of this in the context of networks);

ii) generations of infections in households overlap (Ball et al., 2016).

Because these two factors need to be taken into account to calculate r, no analytical formula to express r in terms of more basic parameters is available in the presence of households (Ball et al., 2016, Appendix G), and model comparison based on r is therefore computationally intensive. Although machine-precision numerical methods for computing r do exist for households models with constant rates of flow between compartments and constant infection rates in each of them (e.g. Pellis et al., 2010), this is not the case for time-since-infection models like the one considered here. Therefore, Fraser (2007) developed an analytical technique to compute r approximately in households models, later extended in Pellis et al. (2010). However, this approximate method ignores exactly the two points highlighted above, i.e. those intricacies that result from the interaction of the household structure with the temporal details of the infectivity profile. Matching the same analytically tractable approximation of r is therefore equivalent to matching the same R_0 in the mapping procedure. The contribution of the two factors above is small for peaked unimodal infectious contact interval distributions (generations tend not to overlap) and low within-household infection rates (repeated infectious attempts between the same infective and susceptible are rare; see Pellis et al., 2010, and Ball et al., 2016). Therefore, the discrepancy between matching the same R_0 and the same r widens for increasing within-household infectivity. In order to quantify such a discrepancy and the implications it has for the mapping procedure, in the Supplementary Methods, Section 1.2.9, we discuss how to compute r using Monte Carlo simulations of within-household epidemics and in the Supplementary Discussion, Section 2.3.2, we use this method to perform the model comparison at fixed r. Only the baseline scenario discussed in the main text is considered, due to the computational cost.

1.1.4 Contact matrices and mixing

The contact patterns between adults and children, both in the household (h) and in the community (g, for "global") is here parametrised in terms of next-generation matrices (NGMs). The general idea of how the NGM is constructed from more fundamental parameters is described in this section, with non-indexed symbols or symbols with generic indices, to stress that the approach is not connected to age classes only or the specific topic of this study. In the next sections, the NGMs for the household and the global infectious contacts are presented with the suitably indexed symbols and the environment-specific details (e.g. accommodating for finite size in the household, etc.).

We found no fully satisfying 1-to-1 parametrisation of the NGM in terms of more fundamental quantities in the literature, i.e. one for which any NGM can be derived from suitably observed quantities, but without redundancy (see Glass et al., 2011, for an exploration of different parametrisations). Therefore we derived a new parametrisation which is good enough for our purposes, but has the serious limitation of not being easily generalisable to more than two groups.

Denote by $i = 1$ or 2 the group index (we have in mind group 2 referring to children) and by c_i the contact rates, i.e. the total number of contacts a single individual in group i has per unit of time (here, a day). Denote by f_{ij} the fraction of contacts that a single type-j individual makes with type-i individuals

$(0 \leq f_{ij} \leq 1)$, so that the matrix of contact rates has the following structure:

$$C = (c_{ij}) = \begin{pmatrix} f_{11}c_1 & f_{12}c_2 \\ f_{21}c_1 & f_{22}c_2 \end{pmatrix} = \begin{pmatrix} f_{11}c_1 & (1-f_{22})c_2 \\ (1-f_{11})c_1 & f_{22}c_2 \end{pmatrix}.$$

Denoting by N_i the number of type-i individuals and assuming that the contacts are undirected, we impose the balancing condition

$$(1-f_{11})c_1 N_1 = (1-f_{22})c_2 N_2$$

stating that the total number of contacts from 1 to 2 equals the total number of contacts from 2 to 1. Note that this assumption is not true in general (there are forms of one-way transmissions, which can be thought of as due to particular contacts, e.g. through fomites, blood transfusions, etc.). However, it is a customary assumption for airborne infections in which bidirectional contacts are thought to be responsible for most transmission events and one-way contacts are difficult to define and therefore measure. Using the balancing condition to eliminate f_{11} and setting $f_{22} = \theta$, we obtained the balanced contact matrix

$$C^* = (c^*_{ij}) = \begin{pmatrix} c_1 - (1-\theta)c_2\dfrac{N_2}{N_1} & (1-\theta)c_2 \\ (1-\theta)c_2\dfrac{N_2}{N_1} & \theta c_2 \end{pmatrix}.$$

Note that θ represents a measure of the assortativity of type-2 individuals, defined as the fraction of the contacts of a type-2 individual that occurs with other type-2 individuals. This definition of assortativity – where $\theta = 1$ gives fully assortative mixing, $\theta = 0$ fully anti-assortative mixing and random mixing is obtain for θ coinciding with the proportion of cases of type 2 in the population – is somewhat not standard in the literature, where usually $\theta = 1$ gives full assortativity and $\theta = 0$ random mixing. Furthermore, note that not all values of θ between 0 and 1 necessarily lead to acceptable contact matrices: depending on the other parameter, certain values of θ might lead to a violation of the natural requirement that $c^*_{11} \geq 0$.

1.1.5 Transmission: next-generation matrices

Denote now by p_{ij} the probability of transmission across a contact from a type-j to a type-i individual. Choosing group 1 as the baseline, we assume that $p_{12} = p_{11}\phi$, $p_{21} = \psi p_{11}$ and $p_{22} = \psi p_{11}\phi$, where ψ and ϕ are, respectively, the relative susceptibility and infectivity of a type-2 versus a type-1 individual. Finally, because the contact rates c_i are defined per unit of time while the elements of a NGM give the total number of infectious contacts during the entire infectious period, we need to multiply all elements of C or C^* by a constant $\xi = T\zeta$, where T is the duration between infection and recovery and ζ denotes the fraction of all contacts occurring during the infectious period that are *infectious contacts*, i.e. that will result in an infection if the contacted individual is susceptible at that time.

One can think, as shown in Supplementary Figure 1, that ζ is the ratio of the area under the infectivity profile over the area of a rectangle at height given by the contact rate c and width given by T. The infectivity profile cannot cross the horizontal line a height c because we assume constant contact rate and transmission occurring with a certain probability during a contact, so that the infectious contact rate is never larger than the contact rate. Note however that T is not uniquely determined: one can double T (i.e. can consider T' in Supplementary Figure 1) assuming that the infectivity is null in the second half of this longer infectious period, but this means that ζ is halved, resulting in the same ξ.

Here we have assumed that individuals of all types have the same infectious contact interval distribution in all environments, so that the infectivity profiles of different types in all environments are proportional to one another and ξ is the same for all types. As mentioned before, this is not necessarily the case (for example, Cauchemez et al., 2011, detected a shorter generation time of children in schools compared to adults in the community), but such level of detail is not considered in this study for a matter of convenience.

Overall, by gathering c_2 and the common factors ξ and p_{11}, we obtain a NGM structure of the form

$$K=(k_{ij})=p_{11}\xi c_2\begin{pmatrix}\frac{c_1}{c_2}-(1-\theta)\frac{N_2}{N_1} & (1-\theta)\phi\\ \psi(1-\theta)\frac{N_2}{N_1} & \psi\theta\phi\end{pmatrix}=\beta\begin{pmatrix}\gamma-(1-\theta)\frac{N_2}{N_1} & (1-\theta)\phi\\ \psi(1-\theta)\frac{N_2}{N_1} & \psi\theta\phi\end{pmatrix},$$

where we have set $\gamma=c_1/c_2$ and $\beta=p_{11}\xi c_2$. Note that the factors in β cannot be separately quantified unless an arbitrary definition of contact is chosen (e.g. a common one is a two-way conversation, see Mossong et al., 2008).

Typically, the next generation matrix cannot be fully specified from epidemic data alone (Glass et al., 2011). The parametrisation above reduces to a minimum the extra information used, but still requires knowledge of γ and θ in all environments, which either needs to be assumed or estimated from contact studies (see Supplementary Methods, Section 1.6.3).

1.2 Age- and household-stratified model

1.2.1 Population structure

The age- and household-stratified (AH) model assumes a population partitioned in two age classes: adults (a) and children (c). We denote by h_{n_a,n_c} the probability that a randomly selected household has composition (n_a,n_c), i.e. consists of n_a adults and n_c children. Although we also consider other populations (see Supplementary Methods, Section 1.6.2), our baseline scenario focuses on Great Britain. The data, based on the 2001 UK census (Office for National Statistics, 2001), is reported in the Supplementary Methods, Section 1.6.1, and in particular the distribution $\{h_{n_a,n_c}\}$ for Great Britain obtained from these data is reported in Supplementary Table 1. From the distribution $\{h_{n_a,n_c}\}$ we derived the distributions $\left\{\pi^a_{n_a,n_c}\right\}$ and $\left\{\pi^c_{n_a,n_c}\right\}$ of the composition of the household of a randomly selected adult and child, respectively, where

$$\pi^a_{n_a,n_c}:=\frac{n_a\,h_{n_a,n_c}}{\sum\limits_{n'_a,n'_c} n'_a\,h_{n'_a,n'_c}}\qquad\text{and}\qquad \pi^c_{n_a,n_c}:=\frac{n_c\,h_{n_a,n_c}}{\sum\limits_{n'_a,n'_c} n'_c\,h_{n'_a,n'_c}} \tag{1}$$

(see Supplementary Table 3).

We denote the total number of adults and children by N_a and N_c respectively, the total population by $N=N_a+N_c$ and the fractions of adults and children by $F_a=N_a/N$ and $F_c=N_c/N$.

1.2.2 Global infectivity

With the parametrisation described in Section 1.1.5, the next generation matrix (NGM) describing transmission in the community is given by

$$K_g=\begin{pmatrix}k^g_{aa} & k^g_{ac}\\ k^g_{ca} & k^g_{cc}\end{pmatrix}=\beta_g\begin{pmatrix}\gamma_g-(1-\theta_g)\frac{N_c}{N_a} & (1-\theta_g)\phi\\ \psi(1-\theta_g)\frac{N_c}{N_a} & \psi\theta_g\phi\end{pmatrix},$$

where k^g_{ij} gives the average number of cases in age-class i an individual in age-class j generates through global infectious contacts only, during the entire infectious period. During the early phase of the epidemic, i.e. when the depletion of susceptible in the community is negligible, all these contacts lead to new infections. Here, γ_g represents the ratio of the numbers c_a and c_c of daily contacts an adult and a child have outside the household, ψ and ϕ are, respectively, the relative susceptibility and infectivity of children versus adults, and θ_g is the (*global*) *assortativity* of children, defined as the fraction of contacts that a child

makes with other children in the community. As pointed out before, this definition of the assortativity is somewhat non-standard, as $\theta_g = 1$ and $\theta_g = 0$ give, respectively, fully assortative and fully antiassortative mixing, and

$$\theta_g = \frac{c_c N_2}{c_a N_1 + c_c N_2} = \frac{N_2}{\gamma_g N_1 + N_2}$$

gives random mixing. (On the contrary, in the literature, assortativity is often parametrised such that $\theta_g = 0$ leads to random mixing.) The multiplicative constant β_g is then chosen to match the desired value of R_0 (see details about the computation of R_0 for model AH in the Supplementary Methods, Section 1.2.4). In the baseline scenario, we use $\gamma_g = 1$ and global random mixing, i.e. $\theta_g = N_c/(N_a + N_c) = F_c$.

We denote by R_0^g the dominant eigenvalue of K_g, which is always smaller than R_0 and gives an intuitive measure of the amount of transmission occurring in the community. The remaining part, $R_0 - R_0^g$, is the component attributable to the within-household transmission, so we define

$$F_h := \frac{R_0 - R_g}{R_0}$$

as the fraction of total transmission occurring in the household, which is explored numerically under different assumptions in Sections 2.1.1 and 2.2.1 of the Supplementary Discussion.

1.2.3 Within-household infectivity

The NGM within a household with n_a adults and n_c children is

$$K_h = \begin{pmatrix} k_{aa}^h & k_{ac}^h \\ k_{ca}^h & k_{cc}^h \end{pmatrix} = \beta_h \begin{pmatrix} \gamma_h - (1-\theta_h)\dfrac{n_c}{n_a} & (1-\theta_h)\phi \\ \psi(1-\theta_h)\dfrac{n_c}{n_a} & \psi\theta_h\phi \end{pmatrix}$$

(see Supplementary Methods, Section 1.1.5, for derivation), where k_{ij}^h gives the average number of infectious contacts an individual in age-class j makes towards individual in age-class i (if there were infinitely many of them in the household, then k_{ij}^h would be the number of new cases of age-class i generated early on in the within-household epidemic). Similarly to the global case, γ_h represents the ratio of the numbers of daily contacts an adult and a child have within the household and θ_h is the (within-household) assortativity of children, defined as the fraction of all contacts a child makes that are with other children in a household of composition (n_a, n_c). Note that the concept of a single within-household assortativity parameter independent of the household composition is not well defined, because the requirement that $k_{aa}^h \geq 0$ might be satisfied in a household of a certain composition, but not of other ones. In order to avoid manual specification of a value of θ_h for each household composition, we assumed random mixing within the household and set $\theta_h = (n_c - 1)/(n - 1)$, where $n = n_a + n_c$ is the household size (a child has $n_c - 1$ other children to contact, out of the $n - 1$ other household members). We assume the biological parameters ψ and ϕ do not change between different environments. The multiplicative constant β_h is then derived from p_{aa} by inverting the construction of p_{aa} described below.

The within-household average numbers of infectious contacts between two specific individuals in a household with composition (n_a, n_c) are given by the matrix

$$\Lambda_{n_a,n_c}^h = \begin{pmatrix} \lambda_{aa}^h & \lambda_{ac}^h \\ \lambda_{ca}^h & \lambda_{cc}^h \end{pmatrix} = \begin{pmatrix} \dfrac{k_{aa}^h}{n_a - 1} & \dfrac{k_{ac}^h}{n_a} \\ \dfrac{k_{ca}^h}{n_c} & \dfrac{k_{cc}^h}{n_c - 1} \end{pmatrix} \tag{2}$$

(provided the above quantities are defined: when they are not defined, e.g. in the case of λ_{cc}^h when there is only one child in the household, they are also not necessary).

The amount of within-household transmission is quantified by β_h, but we re-parametrised the model in terms of p_{aa}, a quantity more common in the literature and referred to as the susceptible-exposure attack-rate in Simpson (1952), secondary attack rate in Cauchemez et al. (2004), or as one minus the susceptible-infectious escaping probability in Fraser (2007). We define p_{aa} as the probability of a randomly selected susceptible being infected directly by a single initial household case, in a randomly selected household with at least two individuals, when adults and children have the same susceptibility and infectivity ($\psi = \phi = 1$). In other words, we first compute $p_n = 1 - \exp(-\beta_h/(n-1))$ for each household size n, and then average p_n over the size distribution of a randomly selected household, conditional on the size being larger than 1. We denote this distribution as $\left\{\tilde{h}_n\right\}$, where

$$\tilde{h}_n := \begin{cases} 0 & \text{for } n = 1 \\ \dfrac{h_n}{\sum\limits_{n>1} h_n} & \text{for } n > 1 \end{cases}$$

(see Supplementary Tables 2, 5 and 7). Different other similar choices would have been possible. Given that p_{aa} and β_h are monotonically related, with $p_{aa} = 0$ when $\beta_h = 0$ and $p_{aa} \to 1$ as $\beta_h \to \infty$ (see Supplementary Figure 2), it is always possible to find the value of β_h required to achieve the desired value of p_{aa}.

Other measures, such as the household secondary attack rate (SAR, stratified either by household size or composition, or averaged over a suitable household distribution), or the fraction F_h of total transmission occurring within the household compared to outside, could be more intuitive. However, they are too aggregate, as they depend on other variables we want to explore independently (such as ψ and ϕ), or, as in the case of the SAR, even on the ratio of adults versus children during the stable exponential growth window, which in turn is a complex model output affected by all other variable choices. In the Supplementary Discussion, Sections 2.1.1 and 2.2.1, we explore the numerical values of these quantities for different scenarios. Eventually, in the main text we provide a rule of thumb that can guide model design directly in terms of the observed SAR, without the need to compute p_{aa} explicitly. This is somewhat surprising, given the amount of complexity captured in the SAR.

1.2.4 Computation of R_0

The computation of the basic reproduction number R_0 for model AH is based on the extension of the technique developed in Pellis et al. (2012) to a model with two types.

For each household composition (n_a, n_c), we compute the probability of each possible epidemic chain using a 2-type Reed-Frost model, with 1-to-1 transmission probabilities given by the elements of $\Lambda^h_{n_a,n_c}$ in Supplementary Equation 2, starting either with a single adult or a single child. For either age class p ($p = a$ or c) of the primary case, we computed the average number $\mu^p_{t,i,(n_a,n_c)}$ of cases of age class t ($t = a$ or c) in each generation i, $i = 0, 1, \ldots, i_{\max}$, where the initial infectives are in generation 0 and $i_{\max}$ the maximum generation index (which is at most $n_a + n_c - 1$, but can be smaller for if there are more initial infectives).

Because time integrated quantities, like R_0 or the final size, depend only on the distribution of the total infectivity, and not on the particular shape of the infectivity profile, the time-since-infection model used here is equivalent to a standard stochastic SIR model (Andersson and Britton, 2000, Section 2) with constant duration of infection period or to a simple Reed-Frost model (Andersson and Britton, 2000, Section 1.2). Therefore, following Equation A.1 of Pellis et al. (2012) (with P replaced here by Q), we define $Q_{(b_a,b_c)}\left((m_a, m_c), (s_a, s_c)\right)$ to be the probability of m_a adults and m_c children out of s_a adults and s_c children escaping infection from b_a and b_c infectious adults and children, which we compute as:

$$P_{(b_a,b_c)}\left((m_a, m_c), (s_a, s_c)\right) = \binom{s_a}{m_a} q_a^{m_a} (1 - q_a)^{s_a - m_a} \binom{s_c}{m_c} q_c^{m_c} (1 - q_c)^{s_c - m_c}.$$

The probability q_a that an adult escapes infection independently from the infectious adults and children is $q_a = q_{aa}^{b_a} q_{ac}^{b_c}$, where the 1-to-1 escaping probabilities can be derived from Supplementary Equation 2 as $q_{aa} = \mathrm{e}^{-\lambda^h_{aa}}$ and $q_{ac} = \mathrm{e}^{-\lambda^h_{ac}}$. Similarly, $q_c = q_{ca}^{b_a} q_{cc}^{b_c}$ with $q_{ca} = \mathrm{e}^{-\lambda^h_{ca}}$ and $q_{cc} = \mathrm{e}^{-\lambda^h_{cc}}$.

Denote by $Y_{t,i,(b_a,b_c),(s_a,s_c)}$ the number of cases of type t ($t = a$ or c) in generation i of an epidemic in a household with s_a and s_c susceptible adults and children and b_a and b_c initial infectives adults and children, and let $\mu_{t,i,(b_a,b_c),(s_a,s_c)} = \mathbb{E}\left[Y_{t,i,(b_a,b_c),(s_a,s_c)}\right]$ be its mean. Then, conditioning on the first generation, for $t = a$ or c we obtain the recursive relation:

$$
\begin{aligned}
\mu_{t,i,(b_a,b_c),(s_a,s_c)} &= \mathbb{E}\left[Y_{t,i,(b_a,b_c),(s_a,s_c)}\right] \\
&= \mathbb{E}\left[\mathbb{E}\left[Y_{t,i,(b_a,b_c),(s_a,s_c)} | Y_{a,1,(b_a,b_c),(s_a,s_c)}, Y_{c,1,(b_a,b_c),(s_a,s_c)}\right]\right] \\
&= \sum_{j_a=0}^{s_a} \sum_{j_c=0}^{s_c} \Big(\mathbb{E}\left[Y_{t,i,(b_a,b_c),(s_a,s_c)} | Y_{a,1,(b_a,b_c),(s_a,s_c)}, Y_{c,1,(b_a,b_c),(s_a,s_c)}\right] \\
&\mathbb{P}\left(Y_{a,1,(b_a,b_c),(s_a,s_c)} = j_a, Y_{c,1,(b_a,b_c),(s_a,s_c)} = j_c\right) \Big) \\
&= \sum_{j_a=0}^{s_a} \sum_{j_c=0}^{s_c} P_{(b_a,b_c)}\left((s_a - j_a, s_c - j_c), (s_a, s_c)\right) \mu_{t,i-1,(j_a,j_c),(s_a-j_a,s_c-j_c)},
\end{aligned}
$$

where s_a, s_c, b_a and b_c are all greater than or equal to 0 (households with no adults or no children are allowed), the number of generations ranges from 1 to $i_{\max} = s_a + s_c$ (the longest possible chain of infections is when, after the initial infectives in generation 0, there is exactly one infective in each generation irrespective of their type), and: $\mu_{t,0,(b_a,b_c),(s_a,s_c)} = b_t$, for $t = a$ or c and $b_a, b_c, s_a, s_c \geq 0$; $\mu_{t,i,(b_a,b_c),(0,0)} = 0$, for $t = a$ or c, $1 \leq i \leq i_{\max}$ and $b_a, b_c \geq 0$; and $\mu_{t,i,(0,0),(s_a,s_c)} = 0$, for $t = a$ or c, $0 \leq i \leq i_{\max}$ and $s_a, s_c \geq 0$. Note that other terms might be null, for example when $s_a - j_a + s_c - j_c < i - 1$, as there can be no infectives in generation $i-1$ if there are not enough susceptibles to have at least one case in each generation till then. However, explicitly accounting for this in the sum indices is cumbersome, so we just let the sums include such null terms. Because we are only interested in within-household epidemics started with a single initial case, we set $\mu^a_{t,i,(n_a,n_c)} = \mu_{t,i,(1,0),(n_a-1,n_c)}$ and $\mu^c_{t,i,(n_a,n_c)} = \mu_{t,i,(0,1),(n_a,n_c-1)}$.

Once we have $\mu^p_{t,i,(n_a,n_c)}$, as done in Pellis et al. (2012) for the single-type model, we then obtain the expected number of cases $\mu^p_{t,i}$ of each age class t in each generation i of the average household epidemic started by a randomly selected primary case in age class p as

$$
\mu^p_{t,i} = \sum_{n_a,n_c} \mu^p_{t,i,(n_a,n_c)} \pi^p_{n_a,n_c}, \qquad i = 0, 1, ..., i_{\max},
$$

i.e. by averaging $\mu^p_{t,i,(n_a,n_c)}$, for either $p = a$ or c, over the composition distributions $\pi^p_{n_a,n_c}$ of the household of a randomly selected individual of age class p (see Supplementary Equation 1 in Supplementary Methods, Section 1.2.1).

In order to compute R_0, individuals need to be distinguished in types according to their potential to infect, and this depends on i) their age class, ii) the age class of the primary case of the household epidemic and iii) the generation they belong to. We denote by $x^p_{n,t,i}$ the average number of individuals in generation n of the whole epidemic, which are of age class t ($t = a$ or c) and belong to generation i ($i = 0, 1, \ldots, i_{\max}$) of a within-household epidemic started by an initial infective of age class p ($p = a$ or c). Given that, for all $i = 0, 1, \ldots, i_{\max}$ and $t, t', p \in \{a, c\}$,

$$
x^p_{n,t,i} = \sum\nolimits_{t'} \mu^p_{t,i} x^p_{n-1,t',0},
$$

we only need to track the dynamics of $x^p_{n,t,0}$, for all $n > 0$, $t, p \in \{a, c\}$. Furthermore, because $x^p_{n,t,0} = 0$ when $t \neq p$ (there are no children infected in generation 0 of a household epidemic started by a single adult, and vice versa), we simply need to track $x^p_{n,p,0}$, $n > 0, p = a$ or c.

In the spirit of Pellis et al. (2012), we consider the (column) vector

$$\mathbf{x_{n,0}} := \left(x^a_{n,a,0}, x^a_{n-1,a,0}, \dots, x^a_{n-i_{\max},a,0}, x^c_{n,c,0}, x^c_{n-1,c,0}, \dots, x^c_{n-i_{\max},c,0}\right)^\top \tag{3}$$

(throughout, $^\top$ denotes matrix transposition) and argue that the dynamics of $\mathbf{x_{n,0}}$ are described by the system

$$\mathbf{x_{n,0}} = M\mathbf{x_{n-1,0}},$$

where the $2i_{\max} \times 2i_{\max}$ block matrix (empty spaces denote 0 entries)

$$M = \begin{pmatrix} \alpha_0^{a,a} & \alpha_1^{a,a} & \cdots & \cdots & \alpha_{i_{\max}}^{a,a} & \alpha_0^{a,c} & \alpha_1^{a,c} & \cdots & \cdots & \alpha_{i_{\max}}^{a,c} \\ 1 & & & & 0 & & & & & \\ & 1 & & & \vdots & & & & & \\ & & \ddots & & \vdots & & & & & \\ & & & 1 & 0 & & & & & \\ \alpha_0^{c,a} & \alpha_1^{c,a} & \cdots & \cdots & \alpha_{i_{\max}}^{c,a} & \alpha_0^{c,c} & \alpha_1^{c,c} & \cdots & \cdots & \alpha_{i_{\max}}^{c,c} \\ & & & & & 1 & & & & 0 \\ & & & & & & 1 & & & \vdots \\ & & & & & & & \ddots & & \vdots \\ & & & & & & & & 1 & 0 \end{pmatrix}$$

(note that the unit elements occupy the subdiagonal, not the main diagonal). The coefficients of M are defined by

$$\alpha_i^{p,p'} := k^g_{p,a}\mu^{p'}_{a,i} + k^g_{p,c}\mu^{p'}_{c,i}$$

and express the fact that an initial household case of type p' in generation $n-i-1$ contributes to primary household cases in generation n by producing $\mu^{p'}_{a,i}$ and $\mu^{p'}_{c,i}$ adults and children in generation $n-1$ (i.e. i generations later), which in turn produce, respectively, $k^g_{p,a}$ and $k^g_{p,c}$ new primary cases of type p in generation n (the infection can only enter a new household via a global infection).

As shown in a slightly different context in Pellis et al. (2012), R_0 can be obtained as the dominant eigenvalue $\rho(M)$ of M. (Throughout, we denote by $\rho(J)$ the dominant eigenvalue, or spectral radius, of a square matrix J.) Because the coefficients depend on β_g via K_g, we can think of R_0 being a function of β_g. Given that $R_0(\beta_g)$ is strictly monotonically increasing, it can be inverted. In addition, $R_0(0) = 0$ (the infection does not exit the households of the initial cases if there is no global transmission) and $R_0(\beta_g) \to \infty$ as $\beta_g \to \infty$, so for each choice of parameters p_{aa}, ψ, ϕ and θ_g, we can compute the value of β_g required to achieve a desired value of R_0.

1.2.5 Incidence ratio of adults versus children in each generation

We denote by $v^{\mathrm{AH}} = (v_a, v_c)^\top$ the vector whose components represent the proportions of adults and children in each generation during the stable exponential growth window, i.e. after they have converged to a stable value, but before experiencing global depletion of susceptibles. To obtain v^{AH} we proceed as follows. In each generation, the components of $\mathbf{x_{n,0}}$, as defined in Supplementary Equation 3, give the average number of household primary cases of each type spread across different generations. They can be used to obtain the average number of cases of either type t in generation n but belonging to each possible household generation i ($i = 0, 1, \dots, i_{\max}$) from the relationship

$$x^p_{n,t,i} = \mu^p_{t,i} x^p_{n-1,p,0}.$$

Summing over p and i we obtain the average number of individuals of age class $t = a$ or c in generation n

$$x_{n,t} = \sum_{i=0}^{i_{\max}} \left(x^a_{n,t,i} + x^c_{n,t,i}\right). \tag{4}$$

The total number of cases in generation n is $x_n = x_{n,a} + x_{n,c}$. During the stable exponential growth window, all these discrete-generation quantities grow geometrically by the same per-generation factor R_0 so that, independently of n, $\mathbf{x_{n,0}} = w$, where

$$w := (w_{0,a}, w_{-1,a}, \dots, w_{-i_{\max},a}, w_{0,c}, w_{-1,c}, \dots, w_{-i_{\max},c})^\top$$

is the *dominant eigenvector* of M, i.e. the eigenvector of M relative to the dominant eigenvalue R_0. Using w in Supplementary Equation 4, we obtain

$$w_t = \sum_{i=0}^{i_{\max}} \left(\mu^a_{t,i} w_{-i,a} + \mu^c_{t,i} w_{-i,c}\right)$$

and, renormalizing, the components $v_a = w_a/(w_a + w_c)$ and $v_c = w_c/(w_a + w_c)$ of v^{AH}. We also denote by v_h^{AH} the vector obtained by renormalizing the components of $K_g v^{\mathrm{AH}}$ so that they sum to 1. This represents the distribution of age classes of any new household primary case in the stable exponential growing window, because individuals make global infections independently of the within-household generation they belong to and the age of their household's primary case.

1.2.6 Household secondary attack rate

The household secondary attack rate (SAR) is a measure of how much transmission occurs in households, and it is readily measurable from data collected in household studies. Although study designs vary, in many cases households are recruited when at least one of their members develops symptoms. The household is then followed for a limited amount of time (usually one or two weeks in the case of influenza, see for example Cauchemez et al., 2004, 2009 or House et al., 2012), while the epidemic is still raging in the general population. It is often very difficult to discriminate whether household cases other than the first have been infected within the household or from the community, so it is often assumed that all observed cases have been infected by other household members, unless time proximity of the first few cases makes direct transmission implausible.

Following Cauchemez et al. (2009) we define SAR as

$$\mathrm{SAR} := \frac{\#\ \text{infecteds}}{\#\ \text{contacts}},$$

where "# contacts" is the number of all initial susceptibles in all households recruited in the study, i.e. the sum of all household members, excluding each single primary case, of all recruited households, and "# infecteds" is the number of those initial susceptibles that acquire infection within the follow-up period.

Consider a household study conducted in an epidemic spreading according to model AH (the "truth"), and further assume that:

1. the study takes place in the early phase of the epidemic (in the stable exponential growth window) so that reintroductions of the infection in previously infected households are rare and can be neglected; and

2. households are followed for long enough to fully observe the first within-household epidemic, started by the single primary case. (After the stable exponential growth window, susceptibles that escaped the first household epidemic can still be infected from the community and infect each other.)

Under these assumptions, it makes sense to define the household secondary attack rate in model AH as

$$\mathrm{SAR}^{\mathrm{AH}} := \frac{\mu^{\mathrm{AH}} - 1}{\chi^v - 1},$$

where χ^v and μ^{AH} are, respectively, the average size of a household infected during the stable exponential growth window in model AH (note the explicit dependence, indicated by the superscript v, on v_h^{AH}, which is explained in detail below) and the average size of an epidemic in such a household.

The rest of this section is devoted to clarifying the reasons for this definition and the precise assumptions we are making.

Denote by $\mathcal{L}$ the set of all households recruited in the study and let $L = |\mathcal{L}|$ be the total number of households (hereafter, $|\cdot|$ denotes denote the number of elements of $\cdot$). Analogously, indicate by $\mathcal{L}_n$, $\mathcal{L}_{n_a,n_c}$ and $\mathcal{L}^p_{n_a,n_c}$ the sets of all recruited households, respectively, of size n, with composition (n_a, n_c), and with composition (n_a, n_c) and primary case of age class p, where $p = a$ or c (note that available data might not contain this last piece of information). Let $L_n = |\mathcal{L}_n|$, $L_{n_a,n_c} = |\mathcal{L}_{n_a,n_c}|$ and $L^p_{n_a,n_c} = |\mathcal{L}^p_{n_a,n_c}|$. Furthermore, define by

$$\hat{\pi}^p_{n_a,n_c} := \frac{L^p_{n_a,n_c}}{L}$$

the observed distribution of households with composition (n_a, n_c) and with a primary case of age class p recruited in the study. Finally, let $n_{\max}$ be the maximum household size and let n^l and Z^l be, respectively, the size of household l $(l = 1, 2, \ldots, L)$ and the size of the observed epidemic in it, including the primary case. Then the observed SAR in the household study is

$$\begin{aligned}
\mathrm{SAR} = \frac{\sum\limits_{l\in\mathcal{L}} \left(Z^l - 1\right)}{\sum\limits_{l\in\mathcal{L}} (n^l - 1)} &= \frac{\left(\sum\limits_{n=1}^{n_{\max}} \sum\limits_{n_a+n_c=n} \sum\limits_{l\in\mathcal{L}_{n_a,n_c}} Z^l\right) - L}{\left(\sum\limits_{n=1}^{n_{\max}} \sum\limits_{l\in\mathcal{L}_n} n^l\right) - L} \\
&= \frac{\sum\limits_{n=1}^{n_{\max}} \sum\limits_{n_a+n_c=n} \left(\sum\limits_{l\in\mathcal{L}^a_{n_a,n_c}} Z^l + \sum\limits_{l\in\mathcal{L}^c_{n_a,n_c}} Z^l\right) - L}{\sum\limits_{n=1}^{n_{\max}} nL_n - L} \\
&= \frac{\sum\limits_{n=1}^{n_{\max}} \sum\limits_{n_a+n_c=n} \left(L^a_{n_a,n_c}\hat{\mu}^a_{n_a,n_c} + L^c_{n_a,n_c}\hat{\mu}^c_{n_a,n_c}\right) - L}{\sum\limits_{n=1}^{n_{\max}} nL_n - L},
\end{aligned}$$

where $\hat{\mu}^p_{n_a,n_c}$ is the observed average size of an epidemic started by a primary case of age class p ($p = a$ or c) in a household of composition (n_a, n_c), which includes the initial case. Dividing both the numerator and the denominator by the total number L of households, we obtain

$$\mathrm{SAR} = \frac{\sum\limits_{n=1}^{n_{\max}} \sum\limits_{n_a+n_c=n} \left(\hat{\mu}^a_{n_a,n_c}\hat{\pi}^a_{n_a,n_c} + \hat{\mu}^c_{n_a,n_c}\hat{\pi}^c_{n_a,n_c}\right) - 1}{\sum\limits_{n=1}^{n_{\max}} n\hat{\pi}_n - 1} = \frac{\hat{\mu} - 1}{\hat{\chi} - 1},$$

where $\hat{\chi}$ and $\hat{\mu}$ are, respectively, the average household size and the average household epidemic size (including the primary case) observed in the study.

In a real household study, the recruitment of households is subject to many constraints, but it ideally monitors a representative proportion of the population of infected households. Assume this ideal condition is met and assume, for the time being, that households of size 1 are included in the survey. Then, if model AH were accurately describing how the infection spreads in the population at large, the following

approximations would hold:

$$\hat{\pi}^p_{n_a,n_c} \approx \pi^p_{n_a,n_c} v^p_h \qquad\qquad p = a \text{ or } c \tag{5}$$

$$\hat{\pi}_n \approx \pi^v_n := \sum_{n_a+n_c=n} \left(\pi^a_{n_a,n_c} v^a_h + \pi^c_{n_a,n_c} v^c_h\right) \tag{6}$$

$$\hat{\mu}^p_{n_a,n_c} \approx \mu^p_{n_a,n_c}, \tag{7}$$

where v^a_h and v^c_h are the components of the vector v^{AH}_h giving the proportions of primary cases of each age class in the stable exponential growth window and

$$\mu^p_{n_a,n_c} = \mu^p_{n_a,n_c}\left(\Lambda^h_{n_a,n_c}\right)$$

is the average size of an epidemic started by an individual of age class p in a household of composition (n_a, n_c). This depends on the within-household infection rates contained in the matrix $\Lambda^h_{n_a,n_c}$ and can be computed either from the average chains of infectives in each generation introduced in the Supplementary Methods, Section 1.2.4, as

$$\mu^p_{n_a,n_c} = \sum_{i=0}^{i_{\max}} \mu^p_{a,i,(n_a,n_c)} + \mu^p_{c,i,(n_a,n_c)}$$

or by averaging the stratified final size distribution for a multitype model in a single (small) population that can be calculated, for example, using the technique described in Addy et al. (1991) in the special case of no infection from outside the household. In general, however, households of size 1 are not recruited in a household study, because they are not useful to estimate the within-household transmission parameters. Therefore, in general, the approximations to use should be $\hat{\pi}^p_{n_a,n_c} \approx \tilde{\pi}^p_{n_a,n_c}$ and $\hat{\pi}_n \approx \tilde{\pi}_n$, where

$$\tilde{\pi}^p_{n_a,n_c} := \begin{cases} 0 & \text{when } n_a + n_c = 1 \\ \dfrac{\pi^p_{n_a,n_c} v^p_h}{C^v} & \text{otherwise} \end{cases} \qquad \text{and} \qquad \tilde{\pi}_n := \begin{cases} 0 & \text{when } n = 1 \\ \dfrac{\pi^v_n}{C^v} & \text{when } n > 1 \end{cases}$$

are the same distributions used before, but conditional on n being larger than 1. The normalising constant C^v is defined as

$$C^v := \sum_{n=2}^{n_{\max}} \sum_{n_a+n_c=n} \left(\pi^a_{n_a,n_c} v^a_h + \pi^c_{n_a,n_c} v^c_h\right)$$

and depends on the elements of the vector v^{AH}_h. (This has to be the case: for example, in the extreme case where global infections hit only adults and adults only live in households of size 1, no household would be recruited in the study and C^v would in fact be 0.) The observed SAR, defined as $(\hat{\mu} - 1)/(\hat{\chi} - 1)$ should now be approximated by

$$\mathrm{SAR} = \frac{\sum_{n=2}^{n_{\max}} \sum_{n_a+n_c=n} \left(\hat{\mu}^a_{n_a,n_c} \hat{\pi}^a_{n_a,n_c} + \hat{\mu}^c_{n_a,n_c} \hat{\pi}^c_{n_a,n_c}\right) - 1}{\sum_{n=1}^{n_{\max}} n\hat{\pi}_n - 1} \tag{8}$$

$$\approx \frac{\sum_{n=2}^{n_{\max}} \sum_{n_a+n_c=n} \left(\mu^a_{n_a,n_c} \tilde{\pi}^a_{n_a,n_c} + \mu^c_{n_a,n_c} \tilde{\pi}^c_{n_a,n_c}\right) - 1}{\sum_{n=1}^{n_{\max}} n\tilde{\pi}_n - 1}. \tag{9}$$

Acting on both the numerator and the denominator, we can multiply both by C^v and add and subtract $(1 - C^v)$ to obtain

$$\mathrm{SAR} \approx \frac{\sum_{n=2}^{n_{\max}} \sum_{n_a+n_c=n} \left(\mu^a_{n_a,n_c} \pi^a_{n_a,n_c} v^a_h + \mu^c_{n_a,n_c} \pi^c_{n_a,n_c} v^c_h\right) - C^v - (1 - C^v) + (1 - C^v)}{\sum_{n=1}^{n_{\max}} n\pi^v_n - C^v - (1 - C^v) + (1 - C^v)}$$

and, noting that

$$1 - C^v = \pi_1^v = \sum_{n_a+n_c=1} \left(\pi^a_{n_a,n_c} v_h^a + \pi^c_{n_a,n_c} v_h^c\right) = \sum_{n_a+n_c=1} \left(\mu^a_{n_a,n_c}\pi^a_{n_a,n_c} v_h^a + \mu^c_{n_a,n_c}\pi^c_{n_a,n_c} v_h^c\right)$$

because $\mu^a_{n_a,n_c} = \mu^c_{n_a,n_c} = 1$ in households of size 1, we can merge the last terms in the first sums of both the numerator and the denominator, obtaining

$$\mathrm{SAR} = \frac{\hat{\mu} - 1}{\hat{\chi} - 1} \approx \frac{\sum_{n=1}^{n_{\max}} \sum_{n_a+n_c=n} \left(\mu^a_{n_a,n_c}\pi^a_{n_a,n_c} v_h^a + \mu^c_{n_a,n_c}\pi^c_{n_a,n_c} v_h^c\right) - 1}{\sum_{n=1}^{n_{\max}} n\pi_n^v - 1} = \frac{\mu^{\mathrm{AH}} - 1}{\chi^v - 1} = \mathrm{SAR}^{\mathrm{AH}}.$$

1.2.7 Final size

The average final size can be computed analytically in the asymptotic limit of an infinite number of households. The methodology is carefully described in Ball et al. (2011). Although they only apply it in the case of a constant recovery rate (i.e. exponentially distributed infectious periods), they reference the work of Addy et al. (1991) for the computation of final size distribution of a within-household epidemic, which is more general and can be applied to the model used here. We briefly report it below, restricting it to the present context, i.e. with two classes and a non-random total infectivity (because the shape of the infectivity profile does not matter for the final size, our time-since-infection model is equivalent to a standard stochastic SIR model where the infectious period has constant duration; see comment in Supplementary Methods, Section 1.2.4).

First, we compute the final size distribution of a within-household epidemic allowing individuals to be infected from outside the household. We denote by $P_{n_a,n_c}\left((u_a,u_c)|\Lambda^h_{n_a,n_c},(q_a,q_c)\right)$ the probability that u_a adults and u_c children will have eventually experienced infection in a household composed of n_a adults and n_c children, who are all initially susceptible, when within-household transmission rates are given by the elements of $\Lambda^h_{n_a,n_c}$ in Supplementary Equation 2 and each adult escapes a constant infection pressure from outside the household, independently of each other, with probability q_a and each child escapes a constant infectious pressure from outside, independently of each other and of each adult, with probability q_c. The probabilities $P_{n_a,n_c}\left((u_a,u_c)|\Lambda^h_{n_a,n_c},(q_a,q_c)\right)$ for $u_a = 0, 1, \ldots, n_a$ and $u_c = 0, 1, \ldots, n_c$ can be obtained numerically by solving the system (Addy et al., 1991)

$$\sum_{u_a=0}^{\ell_a} \sum_{u_c=0}^{\ell_c} \frac{\binom{n_a-u_a}{\ell_a-u_a}\binom{n_c-u_c}{\ell_c-u_c} P_{n_a,n_c}\left((u_a,u_c)|\Lambda^h_{n_a,n_c},(q_a,q_c)\right)}{q_a^{n_a-\ell_a} q_c^{n_c-\ell_c} \left(\mathcal{M}_a(\ell_a,\ell_c)\right)^{u_a} \left(\mathcal{M}_c(\ell_a,\ell_c)\right)^{u_c}} = \binom{n_a}{\ell_a}\binom{n_c}{\ell_c}, \tag{10}$$

for $0 \le \ell_a \le n_a$, $0 \le \ell_c \le n_c$, where

$$\mathcal{M}_a(\ell_a,\ell_c) = \exp\left[-\left((n_a-\ell_a)\lambda^h_{aa} + (n_c-\ell_c)\lambda^h_{ca}\right)\right]$$

and

$$\mathcal{M}_c(\ell_a,\ell_c) = \exp\left[-\left((n_c-\ell_c)\lambda^h_{ac} + (n_c-\ell_c)\lambda^h_{cc}\right)\right]$$

(these function differ from what appears in Ball et al., 2011, because they are the moment-generating functions of the random variable that is here a constant, rather than an exponential).

Denote by z_a and z_c, respectively, the proportion of the population of adults and the proportion of the population of children that are ultimately infected ($0 \le z_a \le 1$, $0 \le z_c \le 1$). Then the infection pressures coming from outside the household a single adult and a single child have to withstand to escape infection altogether are due to the cumulative infectivity of all adults and children infected throughout the epidemic, and in a finite population of size N are approximately given by

$$q_a = \exp\left[-\left(Nz_a\frac{k^g_{aa}}{N_a} + Nz_c\frac{k^g_{ac}}{N_c}\right)\right] = \exp\left[-\left(z_a\frac{k^g_{aa}}{F_a} + z_c\frac{k^g_{ac}}{F_c}\right)\right]$$

and

$$q_c = \exp\left[-\left(z_a \frac{k^g_{ca}}{F_a} + z_c \frac{k^g_{cc}}{F_c}\right)\right],$$

respectively. As the number of households, and hence N, tend to ∞, such escaping probabilities become exact and distinct individuals escape infection independently.

Finally, z_a and z_c are found as the implicit solutions of the system

$$z_a = \sum_{(n_a,n_c)} \pi^a_{n_a,n_c} \frac{\mu_{a,(n_a,n_c)}\left(\Lambda^h_{n_a,n_c} | q_a(z_a,z_c), q_c(z_a,z_c)\right)}{n_a}$$

$$z_c = \sum_{(n_a,n_c)} \pi^c_{n_a,n_c} \frac{\mu_{c,(n_a,n_c)}\left(\Lambda^h_{n_a,n_c} | q_a(z_a,z_c), q_c(z_a,z_c)\right)}{n_c},$$

where the sums are over all possible household compositions and, for either type $t = a$ or c, $\pi^t_{n_a,n_c}$ is the probability that the household of a randomly selected individual of type t given in Supplementary Equation 1 and $\mu_{t,(n_a,n_c)}$ gives the average number of individuals of type t infected in an epidemic within a household with n_a adults and n_c children, who are all initially susceptible and escape infection independently from outside with probabilities q_a and q_c (of which the explicit dependence on z_a and z_c themselves is highlighted), which can be computed after solving the system in Supplementary Equation 10. Note that the system for the final size can be read as a balancing condition, where the left-hand side represents the probability that a randomly chosen initial susceptible of type t is ultimately infected and the right-hand side the proportion of individuals of type t infected in an epidemic within the household of a randomly selected individual of type t, obtained conditioning on the household composition.

1.2.8 Peak incidence and time to the peak

The peak daily incidence and the time to peak incidence are computed by averaging the result of 100 individual-based stochastic simulations in a synthetic population of 100 000 individuals with household composition structure relative to the population under study (in our baseline scenario, Great Britain – see Supplementary Methods, Section 1.6.1 and in Supplementary Tables 1, 2 and 3; for Sierra Leone and South Africa, see Supplementary Methods, Section 1.6.2, and Supplementary Tables 4-7).

Each epidemic starts with $n_0 = 50$ initial cases, to minimise the chance of stochastic extinction and the impact of random delays before the epidemics becomes established (despite this watchfulness, stochastic noise is still visible, in particular in the time to the peak – see Figure 1c in the main text). Furthermore, all initial cases are chosen as primary cases in a different household, and the number of adults and children among them are given by $v^{\mathrm{AH}}_h n_0$ (rounded to the nearest integer), to start the epidemic as close as possible to the stable proportions of cases during the early exponential growth window and limit the impact of initial conditions on the time to reach peak incidence.

Details about the simulation are reported in the Supplementary Methods, Section 1.5.

1.2.9 Monte-Carlo computation of the real-time growth rate

For maximal computational efficiency, the mapping procedure is performed only through time-integrated quantities that do not depend on the particular shape of the infectivity profile and for which analytical results are available. However, we test the robustness of our conclusions by performing the model comparison at fixed real-time growth rate r. Due to the computational cost, this is done only in the baseline scenario. Results are presented in Supplementary Figure 18 and in the Supplementary Discussion, Section 2.3.2.

The computation of r for households models can be done to machine precision for Markovian models (see Pellis et al., 2010), including those involving Erlang-distributed infectious periods or phase-type distributions (i.e. as long as we have flows at constant rates between compartments and constant infectivity

in each of them), but not for time-since-infection models. For this reason, we describe here how to compute r by relying on Monte-Carlo simulations of within-household epidemics run using the Sellke construction (Andersson and Britton, 2000, Section 2.2).

For this purpose we extend the method from Appendix G of Ball et al. (2016) to a model with 2-types, $t = a$ or c. The key ingredients to compute are the average time-varying infection rates $B_{tt'}(\tau)$ at which any infective in the household of a randomly selected primary case of type t' infects, through global contacts only, new susceptible individuals of type t, who therefore are new primary cases in previously uninfected households, τ units of time after the beginning of the within-household epidemic. Denoting by

$$\mathscr{L}_{B_{tt'}}(\sigma) = \int_0^\infty B_{tt'}(\tau)\mathrm{e}^{-\sigma\tau}\,\mathrm{d}\tau$$

the Laplace transform for the function $B_{tt'}(\tau)$, the real-time growth rate can be calculated from the matrix

$$\Omega(\sigma) = \begin{pmatrix} \mathscr{L}_{B_{aa}}(\sigma) & \mathscr{L}_{B_{ac}}(\sigma) \\ \mathscr{L}_{B_{ca}}(\sigma) & \mathscr{L}_{B_{cc}}(\sigma) \end{pmatrix} \tag{11}$$

by imposing that its dominant eigenvalue $\rho(\Omega)$ be 1 (Pellis et al., 2010; Diekmann et al., 2012).

For each household composition (n_a, n_c) and either type of primary case p, consider n_{sim} simulated within-household epidemics started with a single initial infective of type p in a household with n_a adults and n_c children. For each epidemic e, $e = 1, 2, \ldots, n_{\mathrm{sim}}$, let the final number of adults and children be denoted by $Z_a^{p,e}$ and $Z_c^{p,e}$, respectively, and the time of infection of each of the infective adult be $T_{a,j_a}^{p,e}$, $j_a = 1, 2, \ldots, Z_a^{p,e}$. Then an unbiased estimate of $B_{tp}(\tau)$ is given by

$$\hat{B}_{tp}^{n_{\mathrm{sim}}}(\tau) = \sum_{n_a,n_c} \pi_{n_a,n_c}^p \frac{1}{n_{\mathrm{sim}}} \sum_{e=1}^{n_{\mathrm{sim}}} \left[k_{ta}^g \sum_{j_a=1}^{Z_a^{p,e}} \omega\left(\tau - T_{a,j_a}^{p,e}\right) + k_{tc}^g \sum_{j_c=1}^{Z_c^{p,e}} \omega\left(\tau - T_{c,j_c}^{p,e}\right) \right] \qquad (\tau \geq 0),$$

where $\omega(\tau)$ is the probability density function of the infectious contact interval distribution (see Supplementary Methods, Section 1.1.2; also recall that, in this work, ω is assumed to be independent of the types of infector and infectee and to be the same for both global and within-household infectious contacts), $\omega(\tau) = 0$ for $\tau < 0$ (infectives do not contribute to transmission before their time of infection), π_{n_a,n_c}^p is the probability that the household of a randomly selected susceptible of type p infected through a global contact has n_a adults and n_c children, and $k_{tt'}^g$ are the elements of the next-generation matrix of global contacts K_g, i.e. the average number of cases of type t an individual of type t' generates through global infectious contacts only, throughout their entire infectious period (Supplementary Methods, Section 1.2.2).

It is convenient to work directly with the unbiased estimate for the Laplace transform of $B_{tp}(\tau)$

$$\hat{\mathscr{L}}_{B_{tp}}^{n_{\mathrm{sim}}}(\sigma) = \sum_{n_a,n_c} \pi_{n_a,n_c}^p \frac{1}{n_{\mathrm{sim}}} \sum_{e=1}^{n_{\mathrm{sim}}} \left[k_{ta}^g \sum_{j_a=1}^{Z_a^{p,e}} \mathrm{e}^{-\sigma T_{a,j_a}^{p,e}} + k_{tc}^g \sum_{j_c=1}^{Z_c^{p,e}} \mathrm{e}^{-\sigma T_{c,j_c}^{p,e}} \right] \mathcal{M}_\omega(\sigma), \tag{12}$$

where $\mathcal{M}_\omega(\sigma) = \int_0^\infty \omega(\vartheta)\mathrm{e}^{-\sigma\vartheta}\,\mathrm{d}\vartheta$ is the moment-generating function of the infectious contact interval distribution. Inserting Supplementary Equation 12 into Supplementary Equation 11 to obtain an estimate $\hat{\Omega}$ of Ω and solving for $\rho\left(\hat{\Omega}(\sigma)\right) = 1$ yields an estimate $\hat{r}$ of the real-time growth rate.

In order to compute the final size and times of infection for each epidemic, we use the Sellke construction (Andersson and Britton, 2000, Section 2.2) as follows.

Consider a household with n_a adults and n_c children, out of which b_a adults and b_c children are the initial infectives, respectively. Denote by T_{t,j_t} the time of infection of the j_t-th individual of age class t, $j_t = 1, 2, \ldots, n_t$ ($t = a$ or c). All initial infectives are assumed to have been infected at time $\tau = 0$, so that $T_{t,j_t} = 0$ for $j_t = 1, 2, \ldots, b_t$ (in practice we only need the two cases $(b_a, b_c) = (1, 0)$ and $(0, 1)$ for

the computation of r). Each initial susceptible of type t, $j_t = b_t + 1, b_t + 2, \ldots, n_t$, is given a resilience threshold $\mathcal{Q}_{t,j_t} \sim \text{Exp}(1)$. Because susceptible individuals of the same type are indistinguishable, it is convenient to index them by increasing resilience thresholds so that, without loss of generality, for each type t, $\mathcal{Q}_{t,b_t+1} \leq \mathcal{Q}_{t,b_t+2} \leq \cdots \leq \mathcal{Q}_{t,n_t}$. As time τ progresses the total infection pressure, $\Pi_t(\tau)$ say, acting on a each susceptible of type t grows. Individual j_t of type t gets infected at time $\tau = T_{t,j_t}$ at which the accumulated infection pressure on type t reaches their resilience threshold, i.e. $\Pi_t(T_{t,j_t}) = \mathcal{Q}_{t,j_t}$.

Assume we have followed the process until time τ and currently ℓ_a adults and ℓ_c children have been infected up to now (including any initial infective), and $s_a = n_a - \ell_a$ adults and $s_c = n_c - \ell_c$ children are still susceptible. The infection pressure accumulated up to now towards a susceptible of age class t is

$$\Pi_t(\tau) = \lambda^h_{t,a} \sum_{j_a=1}^{\ell_a} \int_0^{\tau - T_{a,j_a}} \omega(\sigma) \mathrm{d}\sigma + \lambda^h_{t,c} \sum_{j_c=1}^{\ell_c} \int_0^{\tau - T_{c,j_c}} \omega(\sigma) \mathrm{d}\sigma, \tag{13}$$

where the transmission rates multiplying each sum are the elements of the matrix $\Lambda^h_{n_a,n_c}$ defined in Supplementary Equation 2 and ω is the probability density function of the infectious contact interval distribution (the same as for global contacts outside the household in this work; extensions to different infectious contact interval distributions in different environments and that depend on the types of infector and infectee are trivial).

The next person to get infected, if any, can only be either the adult with index $j_a = \ell_a + 1$ or the child with index $j_c = \ell_c + 1$, i.e. the susceptible in either group with the lowest resilience threshold. The potential time of infection τ_t of the next susceptible in class t, if no other event happens between now and then, can be found by implicitly solving Supplementary Equation 13 such that $\Pi_t(\tau_t) = \mathcal{Q}_{t,\ell_t+1}$, with the convention that $\tau_t = \infty$ if the maximum infection pressure on type t potentially spread by the current infectives $\Pi_t = \lim_{\tau\to\infty} \Pi_t(\tau)$ is smaller than $\mathcal{Q}_{t,\ell_t+1}$.

If no other infection can be caused in finite time, the epidemic stops. Otherwise, the next event is given by a new infection of type t' corresponding to the minimum τ_t. At that point, the current time is moved forward to $\tau = \tau_{t'}$ and the infection pressure of Supplementary Equation 13 is updated, before checking if someone else will be infected next.

Note that, if both potential times for the next infection τ_a and τ_c are finite and, say, $\tau_a < \tau_c$, then $t' = a$, individual $\ell_a + 1$ is infected at time τ_a and the time τ_c needs to be discarded. However, we know that individual $\ell_c + 1$ will certainly be infected, because the infection pressures available even before the infection of individual $\ell_a + 1$ was already sufficient for that to happen at some point, let alone if we now add the contribution to the total infection pressure of the newly infected adult. In fact, the actual time of infection of the next child can only be anticipated compared to τ_c.

1.3 Age-stratified model

The population of the age-structured model consists of the same numbers N_a and N_c (and proportions F_a and F_c) of adults and children as in model AH, and the transmission between them is parameterised in terms of a single next generation matrix

$$K^{\mathrm{A}} = \begin{pmatrix} k^{\mathrm{A}}_{aa} & k^{\mathrm{A}}_{ac} \\ k^{\mathrm{A}}_{ca} & k^{\mathrm{A}}_{cc} \end{pmatrix} = \beta^{\mathrm{A}} \begin{pmatrix} \gamma^{\mathrm{A}} - (1-\theta^{\mathrm{A}})\dfrac{N_c}{N_a} & (1-\theta^{A})\phi \\ \psi(1-\theta^{\mathrm{A}})\dfrac{N_c}{N_a} & \psi\theta^{\mathrm{A}}\phi \end{pmatrix}. \tag{14}$$

The biological parameters ψ and ϕ are assumed to be the same as in model AH, and γ^{A} is the ratio between the total number of contacts an adult makes both within and between households and the total number of contacts a child makes in all environments. Again, we choose $\gamma^{\mathrm{A}} = 1$ in the baseline scenario.

Neglecting the household structure, however, affects how individuals interact: in particular, if children have substantial interaction with other children and would potentially infect many of them, the limited

number of children in a household forces a child to apparently infect more adults than they would do if there was no household structure. Therefore we need to re-estimate the assortativity θ^{A}, and we do so by imposing that the incidence ratio of adults versus children in model A matches that of model AH, i.e. by imposing the condition $v^{\mathrm{A}} = v^{\mathrm{AH}}$, where v^{A} is the normalised dominant eigenvector of K^{A}. Although the vectors have two elements, they are normalised, so the imposed condition is effectively one-dimensional. Unfortunately, this condition is often not well posed, and there are cases when no value of $\theta^{\mathrm{A}} \in [0, 1]$ leads to a suitable v^{A}. To visualise this problem, the two components of the function $v^{\mathrm{A}}(\theta^{\mathrm{A}})$ and the constant values of the components of v^{AH} are shown in Supplementary Figure 3 for parameters in the baseline scenario ($R_0 = 2$, $\phi = 1$, $\gamma_g = \gamma_h = 1$ and global random mixing, i.e. $\theta_g = F_c$), $p_{aa} = 0.5$ and various values of ψ, suggesting that a unique solution can in general be found when $\psi > 1$, while it can be found only in certain cases when $\psi < 1$. (In reality, the mapping procedure fails also for very large values of p_{aa} and ψ – e.g. see Supplementary Figures 18, 20 and 22.) Further exploration reveals that the behaviour switch observed in Supplementary Figure 3 for $\psi = 1$ occurs in general when $\psi\phi = \gamma^{\mathrm{A}}$, irrespective of R_0, θ_g or the population structure. Indeed, it is easy to verify from the definition of the next generation matrix K^{A} (Supplementary Equation 14) that, for $\psi = \phi = \gamma^{\mathrm{A}}$, $(F_a, F_c)^{\top}$ is always an eigenvector of K^{A}, independently of the value of θ^{A}. Therefore, when $\psi = \phi = \gamma^{\mathrm{A}}$, $v^{\mathrm{A}} = (F_a, F_c)^{\top}$, which is in general different from v^{AH} whenever $p_{aa} > 0$. Hence, no valid assortativity can be found in this case.

In practice, we focus our analysis on the case of $\phi \geq 1$ and $\psi \geq 1$. In this region, most problems with the mapping procedure are avoided. More precisely:

- Under random mixing, $\gamma^{\mathrm{A}} = 1$ and hence the mapping procedure fails for $\phi = \psi = 1$; however, in this case, there is by definition no difference between adults and children in both biological and mixing aspects. We artificially set $\theta^{\mathrm{A}} = \theta_g$ in this case, as this choice has no impact on the predictions of model A, because of the special structure of the NGM.

- When mixing is assortative, data suggests $\gamma^{\mathrm{A}} < 1$ (Supplementary Methods, Section 1.6.3) and hence a valid assortativity can be found for $\phi = \psi = 1$.

- As mentioned above, the mapping procedure does fail for excessively large values of p_{aa} and ψ. However, such values are arguably biologically unreasonable, and this problem was not encountered in the parameter range used when focusing on our baseline population of Great Britain.

Eventually, to allow a more robust exploration, we keep track of parameter combinations for which the mapping fails as, when no value of assortativity for model A can be found, we conclude that households are essential in the model structure to reproduce the observed age-stratified incidence and we discard model A as inaccurate. Using this principle, in the Supplementary Discussion, Section 2.3.3, we explore a wider parameter space, allowing children to be less susceptible and/or less infectious than adults, and hence covering a wide range of values for which the mapping procedure fails.

In Supplementary Figures 10, 20 and 22 we plot the assortativity θ^{A} under different scenarios and we observe how the presence of households can induce both assortative and disassortative mixing. This observation is not new, as cross-generational disassortative mixing (children having strong contacts with carers) is evident in contact survey studies (e.g. Mossong et al., 2008). However, its implications in the present context need to be investigated further.

The basic reproduction number for model A is simply the dominant eigenvalue of K^{A}. Again, the dominant eigenvalue of K^{A} can be seen as a function of the overall multiplicative constant β^{A}. In particular, given any if square matrix J and any scalar ν, the dominant eigenvalue $\rho(\nu J) = \nu\rho(J)$, and so we know that $R_0\left(\beta^{\mathrm{A}}\right)$ is linear in β^{A} and therefore invertible. Because $R_0(0) = 0$ and $R_0(\beta^{\mathrm{A}}) \to \infty$ as $\beta^{\mathrm{A}} \to \infty$, we can always find a suitable value of β^{A} leading to any predefined value of R_0.

The final size of model A can be computed using well-established asymptotic results for multitype models. Adapting from Andersson and Britton (2000) to the present case where the fraction of initial cases in either adults or children is negligible, and using the current notation, the proportions z_a^{A} and z_c^{A}

of the populations of adults and children ($0 \leq z_a^{\mathrm{A}} \leq 1, 0 \leq z_c^{\mathrm{A}} \leq 1$) that are ultimately infected are the implicit solution of the system

$$1 - z_a^{\mathrm{A}} = \exp\left(-\left(\frac{k_{aa}^{\mathrm{A}}}{N_a} N_a z_a^{\mathrm{A}} + \frac{k_{ac}^{\mathrm{A}}}{N_a} N_c z_c^{\mathrm{A}}\right)\right) = \exp\left(-\left(k_{aa}^{\mathrm{A}} z_a^{\mathrm{A}} + \frac{F_c}{F_a} k_{ac}^{\mathrm{A}} z_c^{\mathrm{A}}\right)\right)$$
$$1 - z_c^{\mathrm{A}} = \exp\left(-\left(\frac{k_{ca}^{\mathrm{A}}}{N_c} N_a z_a^{\mathrm{A}} + \frac{k_{cc}^{\mathrm{A}}}{N_c} N_c z_c^{\mathrm{A}}\right)\right) = \exp\left(-\left(\frac{F_a}{F_c} k_{ca}^{\mathrm{A}} z_a^{\mathrm{A}} + k_{cc}^{\mathrm{A}} z_c^{\mathrm{A}}\right)\right).$$

Each of these equations expresses a balancing condition, where the left-hand side represents the probability that a randomly selected initial susceptible of a specific age class is still susceptible at the end of the epidemic, while the right-hand side gives the probability that the susceptible has escaped the infection pressure due to all individuals infected throughout the epidemic, irrespective of their age class (note that k_{ij}^{A}/N_i represents the total infectivity a single infective of type j exerts on a specific individual of type i). It can be proved (Scalia-Tomba, 1986) that when $R_0 > 1$ the system admits a single positive solution (in addition to $z_a^{\mathrm{A}} = z_c^{\mathrm{A}} = 0$, which is always a solution).

The peak incidence and the time to the peak are again computed using individual based stochastic simulations (Supplementary Methods, Section 1.5) with the newly estimated assortativity, no household structure and starting with n_0 initial cases consisting of adults and children in proportions given by the components of the vector $v^{\mathrm{A}} = v^{\mathrm{AH}}$.

Similarly to Section 1.2.9 of the Supplementary Methods, the computation of the real-time growth rate r requires the infectivity profile from an infective of type t' towards any individual of type t ($t, t' = a$ or c), which in model A is $k_{t,t'}^{\mathrm{A}} \omega(\tau)$. Therefore, the matrix with the Laplace transforms of these infectivity profiles can be simplified as

$$\begin{pmatrix} \int_0^\infty k_{aa}^{\mathrm{A}} \omega(\tau) \mathrm{e}^{-\sigma\tau}\, \mathrm{d}\tau & \int_0^\infty k_{ac}^{\mathrm{A}} \omega(\tau) \mathrm{e}^{-\sigma\tau}\, \mathrm{d}\tau \\ \int_0^\infty k_{ca}^{\mathrm{A}} \omega(\tau) \mathrm{e}^{-\sigma\tau}\, \mathrm{d}\tau & \int_0^\infty k_{cc}^{\mathrm{A}} \omega(\tau) \mathrm{e}^{-\sigma\tau}\, \mathrm{d}\tau \end{pmatrix} = K^{\mathrm{A}} \mathcal{M}_\omega(\sigma), \tag{15}$$

where, as for model AH (Supplementary Methods, Section 1.2.9), $\mathcal{M}_\omega$ is the moment-generating function of the infectious contact interval distribution, i.e.

$$\mathcal{M}_\omega(\sigma) = \left(1 + \sigma \frac{T_{\mathrm{G}}}{\alpha}\right)^{-\alpha}, \qquad \sigma > -\frac{\alpha}{T_{\mathrm{G}}}.$$

The real-time growth rate is found by imposing that the dominant eigenvalue of the matrix in Supplementary Equation 15 be 1 and, because $\rho\left(K^{\mathrm{A}}\right) = R_0$ by construction,

$$r = \frac{\alpha}{T_{\mathrm{G}}}\left(R_0^{\frac{1}{\alpha}} - 1\right).$$

1.4 Households model

In the pure households model, individuals are all equal, so the model is parametrised in terms of a global infectivity parameter β_g^{H} and a within-household infectivity parameter β_h^{H}. The parameter β_g^{H} represents the average number of global contacts an individual makes outside the household, throughout the entire infection period: during the stable exponential growth window all such contacts lead to infections of new household primary cases. Similarly, β_h^{H} is defined as the average number of infectious contacts an individual makes in the household (i.e. β_h^{H} would be the number of new cases generated by an individual in a fully susceptible, infinitely large household).

As suggested from households studies, although the person-to-person transmission rate can depend non-trivially on the household size (Cauchemez et al., 2009; House et al., 2012), between the parsimonious

model choices of density- and frequency-dependent transmissions, the latter one is by far more accurate (Cauchemez et al., 2004, 2009). Therefore we assume β_h^{H} to be independent of the household size and we assume that the average number of infectious contacts from an infective to any other randomly selected household member in a household of size n is given by

$$\lambda_n^{\mathrm{H}} = \frac{\beta_h^{\mathrm{H}}}{n-1}.$$

Assuming that in a real-case scenario, the within-household infectivity is measured from household studies, we compute β_h^{H} by matching the household SAR of model AH.

With considerations very similar to those exposed in the Supplementary Methods, Section 1.2.6, we assume that the hypothetical household study from which β_g^{H} would be estimated takes place during the stable exponential growth window and follows households until the first household epidemic finishes. With the same notation as in the Supplementary Methods, Section 1.2.6, the SAR in a household study where adults and children are indistinguishable would be computed as

$$\mathrm{SAR} = \frac{\sum_{l\in\mathcal{L}} \left(Z^l - 1\right)}{\sum_{l\in\mathcal{L}} \left(n^l - 1\right)} = \frac{\left(\sum_{n=1}^{n_{\max}} \sum_{l\in\mathcal{L}_n} Z^l\right) - L}{\left(\sum_{n=1}^{n_{\max}} \sum_{l\in\mathcal{L}_n} n^l\right) - L} = \frac{\sum_{n=1}^{n_{\max}} L_n\hat{\mu}_n - L}{\sum_{n=1}^{n_{\max}} nL_n - L},$$

where $\hat{\mu}_n$ is the average observed epidemic size (including the initial case) in a household of size n. Dividing both the numerator and the denominator by L, we obtain again

$$\mathrm{SAR} = \frac{\sum_{n=1}^{n_{\max}} \hat{\mu}_n\hat{\pi}_n - 1}{\sum_{n=1}^{n_{\max}} n\hat{\pi}_n - 1} = \frac{\hat{\mu}-1}{\hat{\chi}-1}.$$

Under the assumption that model AH represents the truth, if households of size 1 were included in the study, the observed household size distribution $\{\hat{\pi}_n\}$ would be approximately the same as the distribution $\{\pi_n^v\}$. Note that the distribution $\{\pi_n^v\}$ can only be estimated from collected data, i.e. it cannot be constructed from the basic ingredients of model H, as it depends on the vector v_h^{AH}. In other words, the household size distribution of the observed household study is not the household size distribution $\{h_n\}$ nor the size-biased distribution $\{\pi_n\}$ because, for example, children could be more susceptible than adults, and hence more likely to be household primary cases, and they also typically more likely to reside in larger households. Similarly, $\hat{\mu}_n$ should be approximated by μ_n, defined as the average size of a single epidemic in a household of size n infected during the stable exponentially growing window. As a consequence of these two approximations, $\hat{\chi} \approx \chi^v$ (as for model AH), $\hat{\mu} \approx \mu^{\mathrm{H}}$, where $\mu^{\mathrm{H}} := \sum_{n=1}^{n_{\max}} \pi_n^v\mu_n$, and therefore

$$\mathrm{SAR} = \mathrm{SAR}^{\mathrm{H}} := \frac{\mu^{\mathrm{H}}-1}{\chi^v-1}.$$

The average size μ_n of a within-household epidemic in a household of size n can be computed from the full distribution of final sizes, for which standard numerical techniques are available (Ball, 1986; Andersson and Britton, 2000; Addy et al., 1991). In brief, adapting Equation 2.4 of Andersson and Britton (2000) to count the initial infectives directly into the final size, the probability $P_{b,n}(u)$ that an epidemic in a group of size n, started with b initial infectives, results in u individuals ultimately infected (including the initial b, i.e. $u = b, b+1, \dots, n$), with a non-random total infectivity from each infective to each specified susceptible given by λ_n^H, can be obtained as the solution of the system

$$\sum_{b=u}^{\ell} \binom{n-u}{\ell-u} P_{b,n}(u) \Big/ \left[\exp\left(-(s-\ell)\lambda_n^H\right)\right]^u = \binom{n-b}{\ell-b}, \qquad \ell = b, b+1, \dots, n.$$

(The exponential at the denominator is the moment-generating function of a random variable with a constant value). The average final size μ_n is then obtained when there is a single initial infective as $\mu_n = \sum_{u=1}^{n} u P_{1,n}(u)$ for $n = 1, 2, \dots, n_{\max}$.

Given that SAR^{H} depends on μ_n, which is a function of $\lambda_n^H = \beta_h^{\mathrm{H}}/(n-1)$, we are interested in the value of β_h^{H} such that $SAR^{\mathrm{H}} = SAR^{\mathrm{AH}}$, i.e. the household secondary attack rate of model AH. In practice we equivalently find the value of β_h^{H} such that $\mu^{\mathrm{H}} = \mu^{\mathrm{AH}}$. Such a value of β_h^{H} exists and is unique, because $\mu^{\mathrm{H}} = \mu^{\mathrm{H}}\left(\beta_h^{\mathrm{H}}\right)$ is increasing in β_h^{H}, with $\mu^{\mathrm{H}}(0) = 1$ and $\mu^{\mathrm{H}}\left(\beta_h^{\mathrm{H}}\right) \to \chi^v$ as $\beta_h^{\mathrm{H}} \to \infty$ (everyone in the household is infected immediately).

The computation of R_0 for model H follows the method suggested in Pellis et al. (2012). Briefly, following Appendix A of Pellis et al. (2012), first the probabilities $Q_b(m,s)$ that m out of s susceptibles escape infection from b infectives need to be computed ($b, s = 1, 2 \dots$ and $m = 0, 1, \dots, s$). Given the present model with a non-random time-since-infection infectivity profile is equivalent to a simple Reed-Frost model, such probabilities are

$$Q_b(m,s) = \binom{s}{m} (q^b)^m (1-q^b)^{s-m},$$

where the probability q that a single susceptible escapes infection from a single infective is $q = \mathrm{e}^{-\lambda_n^H}$. Then the average numbers $\mu_{i,b,s}$ of cases infected in generation i of a within-household epidemic with b initial infectives and s initial susceptibles can be obtained from the recursive relation

$$\mu_{i,b,s} = \sum_{j=1}^{s-i+1} Q_b(s-j,s)\mu_{i-1,j,s-j}, \qquad b, s = 1, 2, \dots \text{ and } i = 1, 2, \dots, s,$$

with $\mu_{0,b,s} = b$ and $\mu_{i,b,0} = 0$ for all valid b, s and i. (The sum could go up to s, but $\mu_{i-1,j,s-j} = 0$ for $s - j < i - 1$ as there are not enough susceptibles to reach generation $i-1$ with at least one cases in each of them.) The interest is specifically in the average number $\mu_{i,n} = \mu_{i,1,n-1}$ of cases infected in each generation $i = 0, 1, \dots, n-1$ of a within-household epidemic in a household of size n with a single initial infective and $n-1$ initial susceptibles, so it is sufficient to compute $\mu_{i,b,s}$ for $s = 1, 2, \dots, n-1$ and, for each s, for $b = 1, 2, \dots, n-s$ (and i from 1 to s). The average number of cases in each generation are then averaged over the size-biased household size distribution, obtaining $\mu_i = \sum_{n=1}^{n_{\max}} \mu_{i,n}\pi_n$ and, from Theorem 1 of Pellis et al. (2012), R_0 is computed as the dominant eigenvalue of the matrix

$$M^H = \begin{pmatrix} \beta_g^H \mu_0 & \beta_g^H \mu_1 & \cdots & \cdots & \beta_g^H \mu_{n_{\max}-1} \\ 1 & & & & 0 \\ & 1 & & & \vdots \\ & & \ddots & & \vdots \\ & & & 1 & 0 \end{pmatrix},$$

where $n_{\max}$ is the largest household size (note that the unit elements occupy the subdiagonal, not the main diagonal).

The global infectivity β_g^{H} is then computed to match the same R_0 as initially specified in model AH. Again, this is always possible because $R_0\left(\beta_g^{\mathrm{H}}\right)$ is monotonic, with $R_0(0) = 0$ (the infection never exits from an infected household) and $R_0\left(\beta_g^{\mathrm{H}}\right) \to \infty$ for $\beta_g^{\mathrm{H}} \to \infty$.

Once the desired parameters have been obtained, the final size is computed using standard analytical techniques (e.g. Andersson and Britton, 2000; Ball and Neal, 2002). Adapting the notation to resemble more closely the argument in the Supplementary Methods, Section 1.2.7, first denote by $P_n(u|\lambda_h^n, q)$ the probability that u individual will eventually experience infection in a household of size n, where all individuals are initially susceptible, each infective exerts an infection pressure $\lambda_n^H = \beta_h^H/(n-1)$ on each other household member and each individual escapes infection from outside, independently of any other,

with probability q. Such probabilities can be obtained numerically by solving the 1-dimensional equivalent of the system in Supplementary Equation 10, i.e.

$$\sum_{u=0}^{\ell} \frac{\binom{n-u}{\ell-u} P_n\left(u|\lambda_n^H, q\right)}{q^{n-\ell}\left(\mathcal{M}(\ell)\right)^u} = \binom{n}{\ell},$$

for $0 \leq \ell \leq n$, where

$$\mathcal{M}(\ell) = \exp\left(-(n-\ell)\lambda_n^H\right)$$

is the moment-generating function of a constant random variable.

Let z denote the fraction of the population that is ultimately infected by a large epidemic. The infection pressure coming from outside the household a specified susceptible has to withstand to escape infection is due to the cumulative infectivity of all individuals infected throughout the epidemic, and in a finite population of size N is approximately given by

$$q = \exp\left(-Nz\frac{\beta_g^H}{N}\right) = \exp\left(-\beta_g^H z\right).$$

As the population size N tends to ∞, this escaping probability becomes exact and distinct individuals escape infection independently, so z needs to satisfy the self-consistent condition

$$z = \sum_{n=1}^{n_{\max}} \pi_n \frac{\mu_n\left(\lambda_n^H|q(z)\right)}{n},$$

where $\mu_n = \sum_{u=1}^{n} uP^n(u|\lambda_h^n, q(z))$ is the average final size of a within household epidemic where all individuals start susceptible and escape infection from outside with probability q (of which the explicit dependence on z itself is highlighted). Note that the final size can be seen as a balancing condition, where the left-hand side represents the probability that a randomly chosen initial susceptible is ultimately infected and the right-hand side the proportion of individuals infected in an epidemic within the household of a randomly selected individual after conditioning on the household size.

The peak incidence and time to the peak are computed from stochastic simulations starting with n_0 cases, who are all initial cases in different households. Despite adults and children being indistinguishable from an epidemiological point of view, they are chosen in proportions given by the components of the vector v_h^{AH} to ensure that the average size of an infected household is close to χ^v already from the start of the epidemic, thus minimising the impact of transient dynamics on the computation of the time to peak incidence.

Finally, the real-time growth rate r, which is needed to perform the model mapping at constant r instead of R_0 as considered in the Supplementary Discussion, Section 2.3.2, can be computed via Monte-Carlo simulations with the one-dimensional equivalent of the method explained in the Supplementary Methods, Section 1.2.9, which is presented below and is also described, in a slightly different notation, in Appendix G of Ball et al. (2016).

Let $B(\tau)$ be the average infectivity profile of the household of a randomly selected individual who is infected globally, i.e. the rate at which primary cases in new households are infected through global contacts only by any individual infected in a within-household epidemic started by a single primary case that has been infected globally. Then the real-time growth rate can be found as the implicit solution of $\mathscr{L}_B(r) = 1$ (Pellis et al., 2010; Diekmann et al., 2012), where

$$\mathscr{L}_B(\sigma) = \int_0^\infty B(\tau)\mathrm{e}^{-\sigma\tau}\,\mathrm{d}\tau \tag{16}$$

is the Laplace transform of the average household infectivity profile $B(\tau)$, which we need to estimate via Monte-Carlo simulations of within-household epidemics.

For each household size n, consider n_{sim} epidemics with a single initial infective and $n-1$ initial susceptibles. For each epidemic $e, e = 1, 2, \ldots, n_{\text{sim}}$, let $T_1^e = 0$ be the time of infection of the initial infective, Z^e the final epidemic size, including the initial infective, and T_j^e the time of infection of each infective $j, j = 2, 3, \ldots, Z^e$ assuming the epidemic starts at time $\tau = 0$. Then the average household infectivity profile from the n_{sim} epidemics is

$$\hat{B}^{n_{\text{sim}}}(\tau) = \sum_{n=1}^{n_{\max}} \pi_n \frac{1}{n_{\text{sim}}} \sum_{e=1}^{n_{\text{sim}}} \left[\beta_g^H \sum_{j=1}^{Z^e} \omega\left(\tau - T_j^e\right) \right] \qquad (\tau \geq 0),$$

where the infectious contact interval distribution $\omega(\tau) = 0$ for $\tau < 0$. Then an unbiased estimator of $\mathscr{L}_B(\sigma)$ is

$$\hat{\mathscr{L}}_B^{n_{\text{sim}}}(\sigma) = \frac{1}{n_{\text{sim}}} \beta_g^H \sum_{n=1}^{n_{\max}} \sum_{e=1}^{n_{\text{sim}}} \sum_{j=1}^{Z^e} \pi_n \mathrm{e}^{-\sigma T_j^e} \mathcal{M}_\omega(\sigma) \qquad (\sigma > \sigma_0), \tag{17}$$

where π_n is the probability that the household of a randomly selected individual has size n, $\mathcal{M}_\omega(\sigma)$ is the moment-generating function of ω and $\sigma_0 = \inf\{\sigma : \mathcal{M}_\omega(\sigma) < \infty\}$. Solving $\hat{\mathscr{L}}_B^{n_{\text{sim}}}(\sigma) = 1$ numerically gives an estimate $\hat{r}^{n_{\text{sim}}}$ of r.

To compute the final size and time of infections for each epidemic, we use the Sellke construction (Andersson and Britton, 2000, Section 2.2). Consider a household of size n, and let $\mathcal{Q}_j, j = 1, 2, \ldots, n$ be the resilience thresholds associated to each individual j. Set $\mathcal{Q}_1 = 0$ for the initial infective, let $\mathcal{Q}_j \sim \operatorname{Exp}(1)$ for all other $j = 2, 3, \ldots, n$, and assume without loss of generality that individuals are numbered by increasing $\mathcal{Q}_j$.

Suppose we have followed the epidemic until time τ and there are currently ℓ infectives, including the initial one. Then the total infection pressure acting on the remaining susceptibles is

$$\Pi(\tau) = \frac{\beta_h^H}{n-1} \sum_{j=1}^{\ell} \int_0^{\tau - T_j} \omega(\sigma)\,\mathrm{d}\sigma.$$

If $\Pi = \lim_{\tau \to \infty} \Pi(\tau) < \mathcal{Q}_{\ell+1}$, then the epidemic stops because the total infection pressure from the current infectives is insufficient to infect even individual $\ell + 1$, i.e. the weakest of the remaining susceptibles. Otherwise, individual $\ell + 1$ becomes infected at time $T_{\ell+1}$ such that $\Pi(T_{\ell+1}) = \mathcal{Q}_{\ell+1}$ and the total infection pressure needs to be updated to decide whether anyone else is further infected and when.

1.5 Individual-based stochastic simulation

The stochastic simulation is used to calculate those outputs that depend on the particular shape of the infectivity profile and for which no exact analytical result is available for a time-since-infection model, namely the average daily peak incidence and the average time to the peak. The simulation is also used to cross-check the average epidemic final size calculated analytically as described in the Supplementary Methods, Sections 1.2.7, 1.3 and 1.4.

The stochastic simulation is individual-based, i.e. it keeps track of the characteristics of each individual separately. These are: index (ID); age class (adult or child); current infectious state (S, I or R, for susceptible, infectious and recovered); index of the household they belong to (HID); time of infection; and time of recovery. Households are also tracked, and each of them has: an index (HID), the numbers of adults and children in it, and the vectors with the IDs of such adults and children.

The simulation is also event-based, i.e. as the epidemic unfolds new events are generated and stored in a time-ordered list. This allows the simulation to be exact in time even if the model is non-Markovian and hence standard asynchronous algorithms, such as the Gillespie algorithm (Keeling and Rohani, 2008, Section 6.3), cannot be used. Each stored event consists of: time of occurrence; type of event (infectious contact or recovery); infectious contact type, i.e. where it occurred (within-household or global); ID of

the subject (the infector); ID of the object (the infectee); and pointer to the next event in the list. In the case the event is a recovery, the infectious contact type and ID of the object are irrelevant.

Assume that we have followed the epidemic until time τ. If the event at time τ is a recovery, of individual i say, the status of i is changed from I to R and the time of recovery is stored. If the event is an infectious contact from another individual to i and i is still susceptible, the status of i is changed from S to I, the time of infection is stored and the infectious life of i is then constructed as follows.

Consider the case when i is an adult. First, the random numbers of infectious contacts i has with other adults in the same household $Y^h_{a,i} \sim \text{Poi}\left(k^h_{aa}\right)$ and in the community $Y^g_{a,i} \sim \text{Poi}\left(k^g_{aa}\right)$ are drawn. Then, for $j_h = 1, 2, \ldots, Y^h_{a,i}$ and for $j_g = 1, 2, \ldots, Y^g_{a,i}$ the ID of the potential infectee is selected uniformly at random among the (other) adults in i's household and all (other) adults in the population, respectively. If j_h and j_g refer to individuals that are already infectious or recovered at time τ, the event is discarded. Otherwise, if they refer to a susceptible individual, their status is marked as X, to denote they will receive an infectious contact and ultimately be infected, and the time for the infectious contact relative to the infection of i is drawn from the infectious contact interval distribution ω. If the status of the potential infectee is already X, it means the same individual has already received an infectious contact from i (a repeated infectious attempt in the household, which can be relatively common in a small household, a global infectious contact made with a member of the same household or a repeated global infectious contacts made with the same individual – the last two being very unlikely events, but possible in a finite population), so the relative time since the infection of i is drawn from ω and only stored if smaller than the previously stored relative infectious contact time. The same procedure is then repeated for all children in the same household of i and in the community. Finally, all these infectious contact events are stored in the event list and all individuals marked as X are re-labelled as S, because they are all still susceptible at time τ and could potentially be infected by another infective between time τ and the time of the earliest infectious contact from i.

Note that the procedure just described may leads to some infectious contacts being stored that will not result in an infection, e.g. an infectious contact from i to j that occurs at time τ' for a j that is susceptible at time τ (i.e. when the infectious life of i is constructed) but who is infected by another infective between τ and τ'. Then, when the event list is followed that time τ' arrives, j will have already been marked as I or R and no further action is taken.

The initial infectives are infected at time $t = 0$ and the simulation starts by constructing their infectious life.

Maintaining the time-ordered list of events is computationally costly, because the time intervals involved when constructing the infectious life of an individual i remain of the same order of magnitude throughout the epidemic, while the density of events increases towards the peak of the epidemic roughly proportionally with the peak size. The complexity of the algorithm where one single temporal list is searched for the right position where to store an event, which experimentally appears to scale roughly as $O(N^3)$, can be reduced to scale roughly linearly with the population size N by using a *hash table* as follows.

A (small) time interval Δ is chosen and the list of events is broken into a collection of sublists, each one contained in the time interval $[\kappa\Delta, (\kappa + 1)\Delta], \kappa = 0, 1, 2, \ldots,$ so that when a new event is created at time τ, the value of κ corresponding to the time interval in which τ falls is easily found and only the relevant sublist is followed until the right time is reached and the event is stored. If Δ is too small many intervals are empty or almost empty toward the beginning or end of the epidemic, while if Δ is too large time the sublists towards the centre of the epidemic are excessively long and time-consuming to search: therefore, a compromise between memory and computational cost is found for an intermediate value of Δ. For fixed parameter values, such optimal value of Δ is determined by the size of the peak, which grows linearly with the population size N. The optimal constant of proportionality depends on the parameter values in a non-trivial fashion, so it is chosen by timing the epidemics for a number of different parameter combinations to find an acceptable compromise.

After each simulation, which is exact in time, the epidemic is post-processed in discrete time steps of

1 day.

For each parameter combination of interest, 100 realisations of the epidemic in a population of $N = 100\,000$ are performed, with $n_0 = 50$ initial infectives.

A major outbreak is defined as an outbreak infecting more than 1% of the total population, including the n_0 initial infectives. Major epidemics are then shifted in time and synchronised at the day of the peak, in order to superimpose properly their stable exponential growth windows and limit the effects of the random delays at the beginning of each epidemic on the time-point averages of the epidemic curves of all 100 realisations for each model and parameter combination.

The surface of the time to peak incidence (see Figure 1c of the main text) is still highly sensitive to stochastic variations. Limiting this noise is the main reason for the choice of the relatively large number $n_0 = 50$ of initial infectives. Furthermore, initial cases are divided among adults and children as required by the model mapping procedure (see Methods and Supplementary Methods, Sections 1.2.8, 1.3 and 1.4), i.e. in numbers given by the vector $n_0 v_h^{\mathrm{AH}}$ with components rounded to the nearest integers and with each of them being a primary case in a different household. This reduces the time it takes for the proportions of cases of each type to converge, from the initial conditions, to the stable values observed during the stable exponential growth phase, and therefore to highlight structural differences between epidemic speeds in different models.

The epidemic is coded in C++ and each run of 100 simulations requires a few to a few tens of seconds (depending on parameter values, in particular the within-household infectivity) on a common laptop.

1.6 Data and parameter values

1.6.1 Population structure of Great Britain

Based on census data from 2001 (Office for National Statistics, 2001), the population of Great Britain consists of $N = 56\,047\,012$ individuals, divided in adults and children in proportions $F_a = N_a/N = 77.27\%$ and $F_c = N_c/N = 22.73\%$. Supplementary Table 1 describes how adults and children are distributed in households. A dependent child is defined in the Office for National Statistics (2001) to be a person in a household aged 0 to 15 (whether or not in a family) or a person aged 16 to 18 who is a full time student in a family with parent(s). From the distribution $\{h_{n_a,n_c}\}$ of the probability that a randomly selected household has composition (n_a, n_c) (Supplementary Table 1), using Supplementary Equation 1 we obtain the distributions of compositions of the household of a randomly selected adult $\left\{\pi^a_{n_a,n_c}\right\}$ (Supplementary Table 3A) and of a randomly slected child $\left\{\pi^c_{n_a,n_c}\right\}$ (Supplementary Table 3B).

Ignoring the distinction between age classes, from $\{h_{n_a,n_c}\}$ we derive the distribution $\{h_n\}$ of the size of a randomly selected household and, in turn, the distribution $\{\pi_n\}$ of the size of the household of a randomly selected individual (i.e. adult or child in proportions given by F_a and F_c), and the distribution $\left\{\tilde{h}_n\right\}$ of the size of a randomly selected household, conditional of having more than one member.

Finally, from the distributions $\left\{\pi^a_{n_a,n_c}\right\}$ and $\left\{\pi^c_{n_a,n_c}\right\}$, we obtain the distributions $\{\pi^a_n\}$ and $\{\pi^c_n\}$ of the size of the household of a randomly selected adult and children. All these distributions, together with their mean values, are reported in Supplementary Table 2. Note how larger households tend to contain many children, so that the household of a randomly selected child has an average size (4.20 members) which is significantly larger than that of the household of a randomly selected individual (3.07 members).

1.6.2 Other population structures

To test how the conclusions of our analysis are affected by the population structure, as well as to test how applicable they are to other (in particularly, developing) countries, we briefly consider other two populations: that of Sierra Leone, one of the countries with a largest proportion of population consisting of children, and that of South Africa, which offers a intermediate population structure that can be compared with the two extremes.

The distribution of adults and children in households are obtained from the Demographic and Health Surveys Program (ICF), which reports countries' household information, including the age (in years) of all members. Here, all individuals up to the age of 18 (included) have been accounted as children. Although not necessarily the most appropriate choice for a developing country context, this maintains some degree of uniformity with the UK census data and makes the difference with the social structure of Great Britain as striking as possible. This choice results in a fraction of children $F_c = 53.81\%$ for Sierra Leone and $F_c = 45.92\%$ for South Africa, as opposed to $F_c = 22.73\%$ of the Great Britain. Before usage, the data was adjusted by the accompanying weights, according to the database instructions (ICF).

The household composition structure of Sierra Leone, obtained from the dataset published with the 2008 Sierra Leone Demographic and Health Survey (Statistics Sierra Leone – SSL and ICF Macro, 2009), is described in Supplementary Table 4 and summary population distributions are reported in Supplementary Table 5. Instead, the household composition of South Africa, obtained from the dataset published with the 1998 South Africa Demographic and Health Survey (Department of Health/South Africa and Macro International, 2002), is described in Table 6 and summary population distributions are reported in Table 7.

To compromise between accuracy and computational cost, all households with 18 or more members have been ignored and the distribution of household composition has been renormalised. For Sierra Leone (Statistics Sierra Leone – SSL and ICF Macro, 2009) this amounts to cutting 0.558% off the distribution tail, reducing the fraction of children from 53.90% to 53.81%, the average size of a randomly selected household from 5.92 to 5.85 and the average size of the household of a randomly selected individual from 7.46 to 7.24. The 22 individuals with missing age (out of the 41,985 in the dataset from Statistics Sierra Leone – SSL and ICF Macro, 2009) have been counted as children (again, in the spirit of exacerbating the difference with the population of Great Britain): counting them as adults would have led to a fraction of children of 53.76%. For South Africa (Department of Health/South Africa and Macro International, 2002), only 0.0937% of households have 18 or more members. Ignoring those and renormalising the rest of the distribution reduced the fraction of children from 46.00% to 45.92%, the average size of a randomly selected household from 4.28 to 4.27 and the average size of the household of a randomly selected individual from 5.78 to 5.72. The 31 individuals with missing age (out of the 52,906 in the dataset from Department of Health/South Africa and Macro International, 2002) have been counted as children: counting them as adults would have led to a fraction of children of 45.85%.

1.6.3 Contact patterns for the United Kingdom

Our baseline scenario is characterised by random mixing in order to ignore the contribution of age-stratified heterogeneities in the contact patterns. Their presence would make the model comparison unfair, because model H is intrinsically unable to capture them while model A, which is already more flexible and shares two parameters (ϕ and ψ) with model AH, is structurally designed to accommodate for them. However, realistic contact patterns are far from random, so in a real-world scenario the age stratification would be even more relevant than the household structure compared to what the baseline scenario of the study reveals. We argued on it already in Figure 3 of the main text.

We performed a simple analysis of the raw UK data from the POLYMOD study (Mossong et al., 2008) and obtained the estimates in Supplementary Table 8. Assuming for simplicity that all individuals up to 18 years of age (included) are children and discarding all members of households with missing or contradicting information, we obtained 621 adults having 6,885 contacts and 373 children having 5,468 contacts, for a total of 12,353 contacts (sometimes with the same person in different environments).

We used information about all types of contacts (whether physical or not) and ignored any information concerning the duration and intensity of the contacts. In some cases, only a range of possible ages of the contact was given, in which case we used the lower bound of the range. This may have resulted in a possible miscount of the age class of the contact in favour of a child in at most 187 cases (31 at home, 156 outside). A possible cross-check of the estimates obtained here comes from Supporting Information

Table S8.4 (all contacts for Great Britain) of Mossong et al. (2008). A quick calculation of the fraction of contacts an individual 0-19 has with other individuals 0-19 out of their total contacts leads to an estimate of the overall assortativity of children on 58.1%, very close to the 57.9% obtained here in Table 8.

The values of θ_g, γ_h, γ_g and γ^{A} in Supplementary Table 8 are the only ones required to parametrise the NGMs (the remaining values are not used in this analysis). However, their numerical values needs to be treated with care due to the many limitations in the data, most notably:

1. we used both physical and non physical contacts equally;
2. we neglected all information about frequency and duration of the contact;
3. household contacts are defined simply as contacts occurring while at home, irrespective of whether the contacted individual is another household member or not;
4. some of the contact occurred outside the household might have been with a household member.

It is therefore difficult to blindly trust such estimates, so we only use them as rough guidelines of the UK mixing patterns and explore a few intermediate scenarios in the Supplementary Discussion, Section 2.3.1. Note also that we discard the value obtained as the household assortativity of children and instead assume random mixing within the household. The main reason is that the concept of assortativity within the household is not well defined, as it heavily depends on the household composition and a unique value imposed *a priori* might lead to negative elements of the NGM for certain household compositions.

Based on these estimates, we take as mixing parameters for the UK, $\theta_g = 0.58$ and $\gamma_g = \gamma_h (= \gamma^{\mathrm{A}}) = 0.75$ and, for simplicity, use a single parameter $\gamma = \gamma_g = \gamma_h = \gamma^{\mathrm{A}}$ to refer to all of them.

1.6.4 Summary of input parameter values

A summary of the range of parameter values considered in this study is reported in Supplementary Table 9. GB refers to Great Britain, SL to Sierra Leone and SA to South Africa. The baseline population is that of Great Britain, to which baseline values apply. The range 1-4 for ψ is explored in steps of size 0.2 for all populations. For Great Britain p_{aa} ranges in 0-0.95 in steps of size 0.05, while the extended range of values for ψ (Supplementary Discussion, Section 2.3.3) is explored by letting $\log_2(\psi)$ range between -2 and 2 in steps of 0.2. For Sierra Leone, only the range 0-0.475 for p_{aa} is explored, in steps of 0.025. For South Africa, the range for p_{aa} is 0-0.63, in steps of 0.03.

Note that the values of γ_g and γ_h are both 1 under random mixing, and hence $\gamma^{\mathrm{A}} = 1$ too. Furthermore, their estimate from the POLYMOD data (Supplementary Methods, Section 1.6.3) are so close to each other that we deemed it convenient to set both of them to 0.75. Therefore, given that their values are always the same in every context, we conveniently define a unique parameter γ and set $\gamma = \gamma_g = \gamma_h = \gamma^{\mathrm{A}}$ in the rest of the study.

1.6.5 Parameter values for infections explored in the main text

The parameter values used in Figure 3 in the main text should only be regarded as ballparks, because past infections about which we have enough data either did not show community-wide spread (e.g. SARS) or were affected by prior immunity (even the 2009 H1N1 influenza pandemic: see Chen et al., 2009, and Ajelli et al., 2011).

For an infection similar to the H1N1 2009 pandemic influenza we choose $R_0 \approx 1.5$ (Fraser et al., 2009), $\phi \approx 1$, $\psi \approx 2$ (Cauchemez et al., 2009) and a wide range of values for p_{aa} (from 0.1 to 0.4), reflecting a range of SAR estimates found in the literature (House et al., 2012), from <10% (Cowling et al., 2010) to 40% (House et al., 2012). Early, real-time estimates of a SAR of about 13% (Cauchemez et al., 2009) correspond in Supplementary Tables 10 and 12 to a value of $p_{aa} \approx 0.15$.

For parameters in line with the 1918 influenza pandemic, we take from Fraser et al. (2011) values of $R_0 \approx 2$, $\phi \approx 2$, $\psi \approx 1$ and SAR estimates of about 30%, which from Supplementary Tables 10 and 12 corresponds to a $p_{aa} \approx 0.3$.

A measles- or chickenpox-like infection would have an extremely high R_0 and adults almost totally protected by prior immunity, but not necessarily less infectious. Therefore, in Figure 3 in the main text we use $R_0 \approx 4$ and $\phi \approx 1$. There is little information on ψ and p_{aa}, but they relate to the amount of within-household child-to-child infectivity p_{cc} by the equation $1 - p_{cc} = \exp\left(-\psi\beta_h\phi/(n-1)\right) = (1 - p_{aa})^{\psi\phi}$. Simpson (1952) estimated p_{cc} to be about 0.75, thus leading to the range of possible choices of ψ and ϕ presented in Figure 3 of the main text.

2 Supplementary Discussion

2.1 Numerical results with random mixing

2.1.1 Early epidemic indicators and mapped parameters

The amount of within-household transmission has been measured by the parameter p_{aa}, or equivalently by the parameter β_h, to which p_{aa} is biunivocally related (see Supplementary Figure 2). Despite p_{aa} depending in a non-trivial way on the household composition distribution (unlike β_h), we deemed it nevertheless more intuitive that β_h. In practice, neither of them is particularly intuitive. More directly informative quantities could be the household secondary attack rate (SAR) and the proportion F_h of total transmission that occurs within the household. Unfortunately, those quantities also depend, in addition to the household composition distribution, on the relative infectivity ϕ and susceptibility ψ of children versus adults and also on R_0 (in the case of the SAR, only mildly and indirectly on R_0, through the computation of the vector v_h^{AH}). In particular, the dependence on ψ would have made the axes in most plots presented in this manuscript non-independent of each other. To improve quantitative understanding of p_{aa}, we report in Supplementary Tables 10 and 11 both the SAR and the within-household fraction F_h of total transmission, in the baseline scenario of random mixing (values for more realistic mixing patterns are reported in Supplementary Tables 12 and 13 and commented on in the Supplementary Discussion, Section 2.2.1). However, in order to understand better what parameters they depend on, we first discuss the age-stratified incidence.

After that, in this Section we also explore the implications that the household structure has for the apparent assortativity estimated when households are neglected.

Age-stratified incidence. The technique explained in Supplementary Methods, Section 1.2.4, leads to the generation of a matrix M from which both R_0 and the vector $v^{\mathrm{AH}} = (v_a, v_c)$ for the fractions of adults and children in the incidence can be derived. We report in Supplementary Figure 4A the value in percentage of the component v_c of v^{AH} for varying values of R_0, ϕ, ψ and p_{aa}. Note that, for $\gamma = \gamma_g = \gamma_h = 1$ and $\theta_g = F_c$ (baseline values), the global NGM takes the particular structure

$$K_g^{\text{random}} \propto \begin{pmatrix} F_a & F_a\phi \\ \psi F_c & \psi F_c\phi \end{pmatrix},$$

where the constant of proportionality depends only on R_0. Therefore, the value of v_c correctly turns out to be F_c whenever there is no household transmission ($p_{aa} = 0$) and children are as susceptible as adults ($\psi = 1$). Note that this is true irrespective of ϕ as, no matter how infectious adults and children are, new global infections are still generated by both of them in proportions given by (F_a, F_c). This argument holds also for $\psi \neq 1$, although some rescaling is needed, so that $v_c = \psi F_c/(F_a + \psi F_c)$. Note also that the effect of changing R_0 is barely visible. Intuitively, this is the case because changing R_0 only affects K_g, thus modifying only part of the elements of M, from which we then extract the dominant eigenvector

v^{AH}. In addition to this indirect chain of influences, the high sensitivity of the elements of M to changes in ψ and p_{aa} can also contribute in obscuring the already weak influence of R_0 on v^{AH}.

We then report in Supplementary Figure 4B the component v_h^c of the vector $v_h^{\mathrm{AH}} = (v_h^a, v_h^c)^\top$ of proportions of adults and children who start new household epidemics. Note however that, because of the specific structure of K_g^{random} and the fact that $v_h^{\mathrm{AH}} = K_g^{\mathrm{random}} v^{\mathrm{AH}}$, we always have $v_h^c = \psi F_c/(F_a + \psi F_c)$, irrespective of the values of v^{AH} (and therefore of R_0, ϕ and p_{aa}).

Household secondary attack rate. Supplementary Table 10 and Supplementary Figure 4C reports the household SAR for different values of ψ, ϕ and p_{aa}. Each row in each sub-table of Supplementary Table 12 needs to be interpreted with respect to the value of the average size of a household infected during the stable exponential growth window, χ^v, reported in the rightmost column. Because the average size χ^v of a randomly infected household during the stable exponential growth window depends only on the population structure and the vector v_h^{AH}, in the baseline scenario of random mixing it only depends on ψ. The average size of an epidemic in such a household, however, depends also on p_{aa} and ϕ. R_0 has no impact on either of them. Note how the average size of an infected household can be significantly larger than the average size of a randomly selected one or even of the household of a randomly selected individual.

Within-household fraction of total transmission. Unlike the household SAR, the fraction of total transmission that occurs in households depends also on the amount of global transmission and therefore, even under random mixing, on all the parameters. It is reported in Supplementary Table 11 and Supplementary Figure 4D, for different parameter values. It is worth noting that, under baseline random mixing, the rule of thumb that a third of the total transmission occurs in household (Ferguson et al., 2006; Fraser, 2007; Pellis et al., 2010) is approximately satisfied for parameter values of pandemic influenza, at least in the UK (i.e. values of p_{aa} in the highest range for points 1 in Figure 3 of main text, in line with the SAR estimates from the careful analysis of House et al., 2012, concerning Birmingham).

Assortativity of model A. The assortativity θ^{A} for model A is mapped by imposing that model A has the same proportions of adults and children in the incidence as in model AH, i.e. by imposing the condition $v^{\mathrm{A}} = v^{\mathrm{AH}}$. We plot its value for various possible choices of R_0, ϕ, ψ and p_{aa} in Supplementary Figure 10A (see Supplementary Discussion, Section 2.2.1) to invite immediately a comparison between random and assortative mixing. As mentioned in the Supplementary Methods, Section 1.3, the assortativity is not well defined for $\phi = \psi = 1$ under random mixing (although we artificially set $\theta^{\mathrm{A}} = \theta_g$ in this case, as this choice does not affect the results of model A). Therefore, the plot covers only the range $1.2 \leq \psi \leq 4$, i.e. $\psi = 1$ is discarded. Although this is not necessary in the case of assortative mixing or when $\phi > 1$, the same range for ψ is used for convenience and easier visual comparison.

It is interesting to notice that the contribution of the household structure is not at all trivial, as it forces an apparent mixing that, in different parameter regimes, can be either more or less assortative than it actually is. It is also worth noting that these higher and lower apparent assortativity values both appear for strong within-household infectivity, and which one occurs depends almost exclusively on the relative susceptibility of children versus adults.

A better understanding of the interaction between household structure and assortativity is needed, in order to quantify the real impact of household on transmission and on the effect of control policies.

2.1.2 Predicted outputs

In this Section we further explore each output and compare the behaviour of different models, in the baseline assumption of random mixing. In the following sections, we explore deviations from this assumption.

Average final size. As already mentioned in the main text, one of the most striking feature about the final size in model AH is that increasing the difference in susceptibility between adults and children (by keeping R_0 constant) always decreases the final size. This result is already known for multitype models (Miller, 2007; Andreasen, 2011; Andersson and Britton, 2000, Chapter 6), but the same behaviour seems to occur for any fixed amount of within-household transmission. At the same time, shifting transmission from outside to within the household (i.e. increasing p_{aa} while decreasing β_g so that R_0 remains constant) increases the final size, with the negligible potential exception of extremely high values of p_{aa}. All graphs in Supplementary Figure 5e-h appear in the main text, in Figures 1a, 1d, 1g and 2a. Given that for $R_0 = 2$ the final size in absence of households and age stratification is roughly 80% of the population, there is more room for it to decrease due to age differences than for it to increase due to higher within-household transmission. This is indeed visible in Supplementary Figure 5e, by moving along the y-axis and x-axis. Similar qualitative considerations apply to the rest of the graph: for example, at $\psi = 1$, z increases by 10% only over the whole range of p_{aa}, while at $\psi = 4$ it increases by almost 30%, simply because it has more room to do so. The qualitative behaviour is similar for other values of R_0 (Supplementary Figure 5a and 5i) and ϕ (not shown), although at low R_0 (e.g. $R_0 = 1.5$, Supplementary Figure 5a) the room for decreasing is smaller than that for increasing, while at high R_0 (e.g. $R_0 = 4$, Supplementary Figure 5e) the opposite is true.

In terms of comparison, we have already observed in the main text that model A and model H always predict, respectively, a lower and higher final size than model AH (Figures 1a, 1d and 1g of main text). As a consequence, given that at $R_0 = 2$ there is limited room for increasing the final size, model H appears to fail in a larger region of the parameter space, compared to model A (Supplementary Figure 5d, h and l). This could however be explained, at least partially, by model A having 4 free parameters (ψ, ϕ, θ^{A} and β^{A}) and therefore being naturally more flexible than the model H, which has only 2 parameters (β_h^{H} and β_g^{H}). In addition, model A has two parameters (ψ and ϕ) with the same value as in model AH and is somewhat able to partially capture the household structure via re-estimation of the global assortativity. On the contrary, the ability of model H to capture the age structure is almost unnoticeable, as it is limited to the variation in the household size distribution due to age effects in changing v_h^{AH}. Finally, it can be noticed that, because the predictions of models A and H depart from those of model AH in opposite directions, if both of them give accurate predictions, model U must also give accurate predictions and is therefore preferred as simpler. As such, no green regions appear in the simplest model acceptance regions plots in Supplementary Figure 5d, h and l.

Results are qualitatively and quantitatively very similar for other values of ϕ and are therefore not shown.

Average daily peak incidence. The behaviour of the average daily peak incidence for $R_0 = 2$ (Supplementary Figure 5q-t) also appears in Figures 1b, 1e, 1h and 2b in the main text and, for other values, of R_0 in Supplementary Figure 5a-d and u-x. Its qualitative behaviour is essentially the same as that of the final size: in model AH, the peak incidence decreases with increasing age differences and increases with increasing within-household transmission (at fixed R_0). It shows a similar (though milder) feature of having more room to decrease due to age differences than to decrease due to the presence of households. This is somewhat surprising, given that the peak incidence is not capped by any upper bound, as the final size is by the total population. Analogously to the final size, this is less evident at low R_0 (Supplementary Figure 5m-p) and more pronounced at high R_0 (Supplementary Figure 5u-x).

Again, in terms of comparison between models, models A and H predict, respectively, a lower and higher peak incidence than that of model AH. For the same reasons presented before about the final size, once more model A looks able to capture the "true" peak incidence of model AH on a larger region of the parameter space than model H (especially for $R_0 = 4$, Supplementary Figure 5u-x). As for the final size, the fact that models A and H respectively under- and over-estimate the predictions of model AH implies that, in theory, they cannot be both accurate without model U being accurate too. As such, no regions of the parameters space are expected to be coloured in green in the simplest model acceptance regions

plots for the peak incidence. However, unlike the final size z, the peak incidence π is computed using individual-based stochastic simulations and therefore shows some (moderate) stochastic noise, which can in certain cases (e.g. see Supplementary Figure 5p and, as a consequence, Supplementary Figure 7d and, in the main text, Figure 3a, top-left panel) produce a small unexpected green area.

Results are very similar for other values of ϕ and are not shown.

Average time to peak incidence. As for the other outputs, the average time to reach the epidemic incidence peak in model AH for $R_0 = 2$ appears in Figures 1c, 1f, 1i and 2c in the main text, and for other values of R_0 in Supplementary Figure 6a-d and i-l. Note that the duration of a stochastic epidemic is subject to much larger random fluctuations than the final size and peak incidence. As described in the Supplementary Methods, Sections 1.2.8 and 1.5, to reduce the stochastic noise to a minimum, the epidemic starts with $n_0 = 50$ cases, thus essentially excluding the possibility of early epidemic extinction. Furthermore, to reduce as much as possible the effects of system transients from the initial conditions to the stable exponential growth window, we distribute our n_0 as primary cases in different households and divide them in adults and children according to the components of v_h^{AH}. Despite all this watchfulness, some evident stochastic noise is still present (see Supplementary Figure 6a-l, in particular Supplementary Figure 6d). Note, however, that the attempt to reduce stochastic noise by synchronising the epidemics at the peak and starting with n_0 initial cases have the consequence of making variations in the predicted epidemic time to peak very limited overall (within each of the groups a-c, e-g and i-k in Supplementary Figure 6, as differences between groups are caused by the difference in R_0), because most of the variability is due to random delays in the early epidemic phase. The limited variation of average time to the peak (i.e. excluding random delays) was already noted in House and Keeling (2008) for epidemics with the same real-time growth rate r. Given that, at fixed R_0, r varies very little (see Supplementary Methods, Section 1.1.3, and Trapman et al., 2016), we unsurprisingly find similar results.

However, the qualitative behaviour of this output is very different from that of the other two. In particular, the time to the peak t seems to always decrease when increasing either p_{aa} or ψ or both (irrespective of ϕ or R_0). Therefore, peak incidence and time to the peak are positively correlated when increasing variation is susceptibility (constant p_{aa}) and negatively correlated when increasingly shifting the infectivity in favour of within-household transmission.

In terms of comparison, unlike for the previous two outputs, both models A and H tend to overestimate the time to the peak, suggesting that ignoring either form of heterogeneity results in an underestimation of the how fast control policies need to be implemented to stop the spread. Notice also that this time model H seems to perform better than model A at following the pattern of the output of model AH. This is reasonable, as model H can increase the speed of the spread by increasing the within-household infectivity, while model A has no tools to do so. However, as R_0 increases, the limitations of model A are more and more confined to a region of the parameters space corresponding to unrealistically large values of the within-household infectivity p_{aa}.

Because the predictions of both simpler models depart from those of model AH in the same direction (slower spread), it is not surprising the presence of an evident region where either model can be sufficiently accurate but where model U fails.

Again, results are very similar for other values of ϕ, although for increasing ϕ some regions where models A and AH are required for accurate predictions appear and grow in size (not shown).

Age-stratified average final size. In this study we have mostly focused on the overall epidemic final size. However, it may be interesting to consider also the models' predictions in terms of age-stratified final size. We report this output in Supplementary Figure 6m-x, for models A and AH (model H does not distinguish between adults and children, of course) and for different values of R_0 .

As a general trend, model A underestimates the final size predictions in both age classes. As expected, its accuracy decreases for increasing values of p_{aa}. Results are very similar for other values of ϕ.

Comparisons. Supplementary Figure 7 presents the simplest model acceptance regions based on each output separately and joins them (intersects them), so that the simpler models are rejected when at least one output is not accurate enough. Supplementary Figure 7e-h is the same as Figure 2a-d of the main text. Other values of R_0 are shown in Supplementary Figure 7a-d and i-l. In order to show the impact of ϕ, Supplementary Figure 7m-x reports the same output as in Figure 7a-l, but for $\phi = 2$.

2.1.3 Other rejection thresholds

In the main text we have rejected a simple model when its output is more than 5% different from that of model AH. Here we explore other possible values for this rejection threshold ε.

In Supplementary Figure 8 we explore the simplest models acceptance regions for $\phi = 1$ and R_0 ranging from 1.5 to 4, but for rejection thresholds $\varepsilon = 1\%$ and $\varepsilon = 10\%$ (first and second row, respectively). Results should be compared to those obtained with the 5% tolerance used in Supplementary Figure 7a-l. For ε =10%, it can be easily noted how the approximation of all simpler models improve for large R_0: in particular, the strongly non-linear dependence of the final size z on R_0 causes all models to give very similar predictions for it in a large region of the parameters space (the blue area of Supplementary Figure 8i).

Apart from accepting model U on a wider region of the parameter space, the figures for ε =10% also reveal another interesting observation. We have already noticed how model A is in general better able than model H to follow the qualitative predictions of model AH (see Figure 1 in the main text). This aspect becomes even more evident for $\varepsilon = 10\%$ (Supplementary Figure 8m-x) when, especially for increasing R_0, the region where model A is the simplest (light blue) becomes much wider while the one where model H is the simplest (yellow) shrinks until disappearing. All results are very similar when $\phi > 1$ (not shown).

Finally, in Supplementary Figure 9 we summarise the overall simplest model acceptance regions plots for ε =1%, 5% and 10%. In line with what just said, Supplementary Figure 9 suggests how the presence of households become much less relevant for higher values of R_0, while the age stratification remains important in vast areas of the parameter space. This is true despite the random mixing assumption. In the next section we show how this conclusion is accentuated even further in the case of non-random mixing.

Finally, note how the border between the light blue and red regions, indicating where households are needed in addition to age classes, roughly follows the SAR isoclines, though for different SAR values at different values of R_0 and of the threshold ε.

2.2 Numerical results with realistic UK contact patterns

In this section we illustrate how most of the results shown before change if the realistic UK contact patterns are assumed (see Supplementary Methods, Section 1.6.3).

2.2.1 Early epidemic indicators and mapped parameters

When mixing does not occur at random, all early epidemic indicators and mapped parameters depend on all other parameters. We report in Supplementary Figure 11A and B the child components v_c and v_h^c of the vectors v^{AH} and v_h^{AH}, respectively. The striking difference with Supplementary Figure 4A and B (note the change in the colour scale) is how much more focussed on children the spread is, and this is a direct consequence of the fact that children have more contacts than adults on average, and that those contacts are more likely to be with other children (assortative mixing).

This striking difference has apparently only mild repercussions on the household SAR (Supplementary Table 12 and Supplementary Figure 11C), but changes noticeably the average size of an infected household (compare the values of χ^v in Supplementary Tables 10 and 12), because children are preferentially infected and they tend to live in larger households. The fraction of total transmission that occurs in households

(compare Supplementary Tables 11 and 13, or Supplementary Figure 4D and 11D), however, seems to be only moderately affected, leading again to the conclusion that, also with UK contact patterns, for parameters of pandemic influenza in the UK (House et al., 2012), roughly a third of total transmission occurs in households.

With UK contact patterns, the assortativity of children is already quite high, so the apparent value obtained when the household structure is removed seems to be consistently lower than its original value for different values of R_0 and ϕ (Supplementary Figure 10B), unlike what can be observed for random mixing (Supplementary Figure 10A). Again, a better understanding of this phenomenon and its implications needs to be gained.

2.2.2 Predicted outputs

In Supplementary Figures 12 and 13 we plot the three main outputs and the age-stratified final size for models AH, A and H, together with the simplest model acceptance regions, when adults and children mix according to UK contact patterns. The values of R_0 explored are 1.5, 2 and 4, $\phi = 1$ and $\varepsilon = 5\%$.

The plots for the final size z and the peak incidence π are qualitatively similar to those shown in Figure 1 in the main text, where random mixing is assumed (though the colour scale are different, and are kept fixed between different R_0 values in Supplementary Figures 12 and 13 to facilitate the comparison). It is now striking how far the predictions of model H are from those of model AH. In particular, for $R_0 = 2$ (Supplementary Figure 12e-h) almost nowhere model AH achieves a final size larger than the 80% obtained under pure random mixing (U model). Therefore, model H, which cannot predict anything smaller than 80% leads to an almost flat surface in Supplementary Figure 12g. The same observations hold for the peak incidence (Supplementary Figure 12s). In terms of time to the peak, instead, the presence of heterogeneous mixing tends in general to make the spread faster, in particular for large values of ψ even when p_{aa} is small (compare Supplementary Figure 13e and Figure 1c of the main text).

In terms of age-stratified final size, model A still underestimates predictions for both age classes, especially for large p_{aa}, though the discrepancy is minimal.

Supplementary Figure 14 now reports the simplest model acceptance regions based on each output and on all output together for an $\varepsilon = 5\%$ rejection threshold and $R_0 = 1.5$, 2 and 4, and $\phi = 1$ and 2. The patterns appear to be more complex than in the case of random mixing, but they overall tend to reject model H in most cases and require an age stratification almost everywhere, especially for smaller R_0 or larger values of ϕ.

Further support in this direction comes from Supplementary Figure 15, where thresholds of 1% and 10% are used ($\phi = 1$) and from Supplementary Figure 16, where the overall simplest models acceptance regions are plotted for different values of R_0, ϕ, ψ and p_{aa}, and for rejection thresholds of $\varepsilon = 1\%$ (A) 5% (B), and 10% (C). Apart from the absolute need to have at least the age structure when the tolerance is low ($\varepsilon = 1\%$), it is striking how, even for high tolerance ($\varepsilon = 10\%$) the age structure is almost always necessary.

2.3 Further sensitivity analysis

2.3.1 Intermediate contact patterns

The difference between the simplest model acceptance region plots of Figures 3A and 3B of main text is striking because UK contact patterns are very different from random ones. To illustrate the gradual transition between these two extremes, we plot in Supplementary Figure 17 the simplest model acceptance region plots for intermediate values of the assortativity ($\theta_g = 0.4$ and 0.5, between the 0.2273 of random mixing and the 0.58 of the assortative mixing measured in the UK) and for equal or increased number of contacts children make compared to adults: γ $(= \gamma_g = \gamma_h) = 1$ or $\gamma = 0.75$ (children have 33% more contacts than adults in all environments). As expected, Supplementary Figure 17 shows a gradually increasing importance of the presence of an age structure compared to the household structure.

2.3.2 Accuracy of model mapping: fixed R_0 versus fixed r

As mentioned in Section 1.1.3, the real-time growth rate r is in general the most readily available quantity early on in an epidemic. Therefore, a natural choice would be to match all models to this quantity. In Figure 18 we show the results when the mapping is performed by keeping fixed r.

Although fast machine precision results exist for calculating r for households models when the disease progression occurs by transitioning through a series of compartments (Pellis et al., 2010), this is not the case for the current model, where a fixed (deterministic) time-since-infection (Γ-shaped) infectivity profile is used. In this case, no exact result is available. Approximate methods for computing r in households models exist (Fraser, 2007; Pellis et al., 2010) but matching the same approximate r is exactly the same as matching R_0 as we have done here (see Ball et al., 2016, and Supplementary Methods, Section 1.1.3). Therefore, we rely on Monte Carlo methods to compute r approximately, as described in the Supplementary Methods, Section 1.2.9, for model AH, and Supplementary Methods, Section 1.4, and Appendix G of Ball et al. (2016) for model H.

Given the computational cost, only 100 simulated within-household epidemics for each household composition and either type of initial infective are performed for each parameter combination. Results, therefore, are unavoidably inaccurate and present a significant level of noise (see Supplementary Figure 18). Therefore, despite r being a more intuitive parameter to keep fixed between models, the noisy output and the computational cost are both valid reasons supporting the choice made here of performing the model comparison at fixed R_0.

Broadly, Supplementary Figure 18 confirms the intuition developed in the comparison at fixed R_0. However, because keeping fixed R_0 is the same as keeping fixed the approximation of r of Fraser (2007) and Pellis et al. (2010) and it is known that such approximation is less accurate when household transmission is strong (see Ball et al., 2016), the discrepancy between the results of Figure 18 and Figures 9 and 16 increases for large p_{aa}. The three values or r investigated (r =0.14552, 0.25282 and 0.52588) correspond, respectively, to $R_0 = 1.5$, 2 and 4 at $p_{aa} = 0$, i.e. when there is no household transmission.

The light-shaded red square visible on the top-right panel for $R_0 = 1.5, \psi = 1$ and random mixing denotes a parameter combination for which the mapping procedure fails as no value of the assortativity θ^{A} can be found (see Supplementary Methods, Section 1.3). This case occurs more commonly in Supplementary Figures 19-23, so it is discussed more extensively in Supplementary Methods, Sections 2.3.3 and 2.3.4.

2.3.3 Children less susceptible and/or infectious than adults

Throughout this study, we have focussed on children being more susceptible and/or more infectious than adults, because this seemed to guarantee that a valid value for the assortativity θ^{A} of model A could be found. As explained in the Supplementary Methods, Section 1.3, this is not strictly true, as the main problem arises for $\phi\psi = \gamma$ under random mixing and when ψ and p_{aa} become large enough (see Supplementary Figures 20 and 22A, top-left panel). However, the situation is significantly more complex when children are assumed to be less susceptible and/or less infectious than adults. In Supplementary Figure 19 we allow ψ to vary between 0.25 (children a quarter as susceptible as adults) to $\psi = 4$ – note the $\log_2$ scale on the y-axes – and we consider $\phi = 0.5$ (children half as infectious as adults), 1 and 2. The colours are the same as in all other figures, but the lighter-shaded areas identify the regions of the parameter space where no value of θ^{A} could be found. This implies that, for those parameter values, model A was unable to reflect the observed stratified incidence and was therefore discarded as inaccurate. Therefore, no lighter shaded area appears in regions coloured in light blue (nor in those in green). Note however that it is possible to have parameters to the right of the thick line (i.e. where households are needed to reproduce the observed stratified incidence) for which the simplest unstructured model U (blue region) is sufficient for accurate predictions. This is the case because model U is not required to match any other condition but the correct value of R_0.

Note the visible horizontal spikes occurring when $\psi\phi = \gamma$ (see Supplementary Methods, Section 1.3), which should but might sometimes not appear to extend throughout all values of p_{aa} only because of the limited (p_{aa}, ψ)-grid resolution. However, despite such spikes, the border between the light blue and the red areas seem in general to follow the SAR isoclines throughout the whole range of values of ψ explored, thus suggesting the results of these analysis somewhat extend also to the case of children less susceptible and/or infectious than adults. The problem of matching the assortativity does unavoidably complicate the picture but, within the resolution of Supplementary Figure 19, regions to the right of the thick line but left of the SAR isocline separating red and light blue regions seem to consistently be (shaded) dark blue areas. Therefore, Supplementary Figure 19 seems to suggest that one can still adopt the following approach:

1. Follow the rule of thumb (Results section of main text and Supplementary Discussion, Section 2.4) to decide, based on measures of SAR, whether households are needed. If yes, include households and age.
2. If not, see whether model A can reproduce the observed stratified incidence. If yes, use model A.
3. If not, use model U.

2.3.4 Other populations

Sierra Leone. The population of Sierra Leone is dramatically different from that of the UK, in particularly because of the very large fraction of children. Its household composition structure (Statistics Sierra Leone – SSL and ICF Macro, 2009; see Supplementary Methods, Section 1.6.2) is described in Supplementary Table 4 and the summary population distributions are reported in Supplementary Table 5.

The mapped value for the assortativity of model A is plotted in Supplementary Figure 20 for random (A) and assortative (B) mixing. Note that a large household size and fraction of children implies that outputs are more sensible to variations in p_{aa}, which therefore is plotted only up to 0.475. Furthermore, to facilitate the comparison with Supplementary Figure 10 for Great Britain (as well as Supplementary Figure 22 below for South Africa) the colour axis has been kept the same in all these plots. Note also the presence of a white region, for very large p_{aa} and ψ (top-right of Supplementary Figure 20A), where no valid mapped value for the assortativity can be found.

Given the illustrative purpose of the analysis to other populations, as well as the lack of available data, assortative mixing is assumed to follow the same contact patterns as for the UK. Note that this is a significantly less strong a requirement than for Great Britain: because the fraction of children is much larger in Sierra Leone, a larger assortativity is associated to random mixing ($\theta_g = F_c = 53.81\%$). Requiring that 58% of contacts of a child occurs with other children still implies that mixing that is more assortative than random, but the difference is almost negligible compared to the case of the Great Britain. For this reason, an assortativity of 70% has also been explored, but results are similar and hence not shown.

Qualitatively, although under random mixing (Supplementary Figure 20A) the household structure can impose both assortative and disassortative mixing as in the case of Great Britain, an increase in the apparent assortativity occurs very rarely (only for $\phi = 1$ and some values of $\psi < 1.5$). For already assortative mixing (Supplementary Figure 20B) the household structure only reduces the apparent assortativity.

Supplementary Figure 21 shows the overall simplest model acceptance region plots for Sierra Leone, which leads to the same qualitative conclusions as for Great Britain and thus suggests the results of our analysis are generally applicable to very diverse populations and social structures. The region where the mapping to model A fails, i.e. the region characterised by a lighter colour shading, is significantly larger that the region where no valid assortativity $0 < \theta^{\mathrm{A}} < 1$ could be found, as some values of θ^{A}, though valid, lead to a negative, and hence unviable, adult-to-adult component of the NGM ($k^{\mathrm{A}}_{aa} < 0$).

South Africa. The household composition structure of South Africa (Department of Health/South Africa and Macro International, 2002; see Supplementary Methods, Section 1.6.2) is described in Supplementary Table 6 and summary population distributions are reported in Supplementary Table 7.

As for Sierra Leone, we monitor the mapped assortativity of model A in Supplementary Figure 22 for random mixing (A) and UK-like mixing patterns (B). The within-household infectivity p_{aa} goes only up to 0.66. Qualitative conclusions are the same as before, though quantitative differences are highlighted by the use of the same scale as in Supplementary Figures 10 and 20. Note again the presence of a region where no valid assortativity θ^{A} could be found (top right of Supplementary Figure 22A).

Supplementary Figure 23 shows the overall simplest model acceptance region, for random (A) and UK-like mixing patterns (B), and leads once more to the same conclusions as for the population of Great Britain. Note again a region where the mapping to model A fails because, despite a valid assortativity being found, it leads to negative values for k_{aa}^{A}.

2.4 Rule of thumb

As visible from Figure 3B in the main text, the border between the parameter regions where households are (red) and are not (light blue) needed for predictions within 5% relative accuracy strongly resembles the contour lines of the SAR for the entire range of values of ψ explored. Different contour lines appear to be relevant for different values of R_0 but variations in ϕ seem to be negligible. Somewhat surprisingly, also the choice of whether random mixing or UK-like contact patterns are assumed (compare Supplementary Figure 9 and Supplementary Figure 16) seems to bear negligible influence on the relationship between R_0 and the SAR above which households are important. Note however that, if a region where the age structure is not needed for accurate predictions is present (dark blue and yellow), often model U can provide accurate results even to the right of the thick line in Figure 9, because the contribution of households and that of the age structure cancel each other out. In other words, in drawing the boundary discriminating when households are important or not we have only focussed on whether the difference in predictions from model AH and A is more than ε, irrespective of whether then model U is still selected as the simplest model choice.

We were not able to find a relationship between parameters and model output suggesting when the age structure was necessary for accurate predictions that was as simple as that for the household structure. However, because for realistic UK-like contact patterns the presence of the age structure was generally necessary for accurate predictions, and because age-stratified models are generally mathematically simpler to study than households models, we deemed providing such a criterion less relevant in the first instance.

To illustrate the strong relationship between R_0 and the level of SAR above which households are important, we constructed Figure 4 in the main text as follows. First, we generated the data points according to the following algorithm.

1. We fixed the population structure (Great Britain, Sierra Leone, South Africa) and the global assortativity θ_g (random – which is population dependent – or UK-like assortative, i.e. $\theta_g = 0.58$).

2. For every value of $R_0 \in \{1.1, 1.3, 1.5, 1.7, 2, 2.3, 2.7, 3.2, 4\}$ and $\phi \in \{1, 1.5, 2\}$, we generated the data necessary for any (p_{aa}, ψ) plot in this study, in particular the value of all outputs (final size z, peak incidence π and time to the peak t) for model AH and for model A, after finding θ^{A} from the mapping procedure described in the Supplementary Methods, Section 1.3. If no assortativity was found, the outputs of model A were always considered inaccurate, i.e. more than ε away from those predicted by model AH (hence why the bold line follows the light-shaded area in Supplementary Figure 19).

3. The minimum relative error between output predictions of models AH and A was then calculated as

$$m_{\mathrm{err}} = \min \left\{ \frac{\left| z^{\mathrm{AH}} - z^{\mathrm{A}} \right|}{z^{\mathrm{AH}}}, \frac{\left| \pi^{\mathrm{AH}} - \pi^{\mathrm{A}} \right|}{\pi^{\mathrm{AH}}}, \frac{\left| t^{\mathrm{AH}} - t^{\mathrm{A}} \right|}{t^{\mathrm{AH}}} \right\}.$$

4. An ε-contour line of m_{err} was then generated using the `contourc` function in MATLAB and the contour matrix was stored. Such a matrix contains the (p_{aa}, ψ) points that are joined together to plot the contour line.

5. The contour matrix was "cleaned" as follows. In the presence of noisy output, some red squares might appear inside the light blue area and fully detached from the main red region (see e.g. top-right of Figure 3B of main text, as well as many others). The contour matrix then contains different components, one for every (if any) disconnected small red region inside the light blue region (generating a small, closed contour line visible in many plots) and a main component separating the entire red and light blue regions (long, open line spanning the entire range of ψ). The former components were discarded and only the latter was retained.

6. For every point of the "cleaned" contour line, the SAR was computed and its values plotted against R_0.

Second, we plotted the regression lines as follows. It is evident that, although a straight line fits the data well, it is inaccurate around $R_0 = 1$ (where a threshold transition is expected) and for high R_0 because of saturation effects. Furthermore, the range of R_0 where a linear fit was deemed appropriate, inevitably depends on the threshold ε chosen. Therefore we ran a preliminary analysis (see Supplementary Figure 24 for the case of $\varepsilon = 5\%$, other values were explored similarly) to visually asses an appropriate range of at least three consecutive R_0 values to be used in the linear fit. More specifically, for both random (Supplementary Figure 24A) and UK contact patterns (Supplementary Figure 24B), we fitted a linear regression model to all data points corresponding to a fixed ϕ and a fixed range of ψ values (each of the nine subpanels in Supplementary Figure 24A and B), for a range of R_0 values starting from the one denoting each row and terminating on the one denoting each column (e.g. the top-left cell corresponds to R_0 values of 1.1, 1.3 and 1.5). The regression coefficient and intercept are reported in each cell and the adjusted R^2 is visually expressed by the cell colour. From Supplementary Figure 24 and similar ones corresponding to $\varepsilon = 1\%$ and $\varepsilon = 10\%$ (not shown) we decided empirically that a suitable range of R_0 values for a linear fit for all populations, with both random mixing and UK-like contact patterns was:

- for $\varepsilon = 1\%$: $R_0 \in [1.3, 2.7]$;
- for $\varepsilon = 5\%$: $R_0 \in [1.3, 3.2]$;
- for $\varepsilon = 10\%$: $R_0 \in [1.3, 2.5]$.

Note that the spread of the distribution of data points for each value of R_0 is a result of multiple factors:

- The lack of smoothness of the contour line visible in each plot: this is fundamentally due to the coarseness of the (p_{aa}, ψ)-grid resolution, which can be easily improved at increased computational cost;
- The fact that data points coming from numerous values of ϕ and ψ are pooled together: this is also visible in the slight variations in the goodness of fit in each of the nine sub-panels in Supplementary Figure 24A and B.

The latter reason motivated keeping track of which values of ϕ and ψ are associated with each data point: this more careful analysis revealed that most extreme values in the distribution of data points correspond to joint large values of ϕ and ψ, which are biologically less realistic. Therefore, we distinguished data points for which $\phi\psi \leq 3$ (more realistic, plotted in black) and the others (grey), and we separately fitted a linear regression model to each of these subgroups (the dashed lines in Figure 4 in the main text and in Supplementary Figure 25, each fitting only the data points of the corresponding colour, for the values of R_0 specified before).

In devising an empirically reasonable rule of thumb, i.e. in proposing some coefficients for the thick line in Figure 4 in the main text and in Supplementary Figure 25, we aimed at a good fit, with the same line for the same population, of both the data points relative to random mixing and those relative to UK-like contact patterns with assortative mixing (top and bottom lines, respectively, in Supplementary Figure 25A, B and C). More weight has been given to the more realistic UK-like assortative mixing and to the black data points ($\phi\psi \leq 3$), as biologically more plausible. This is evident, as the thick line in Supplementary Figure 25A, B and C is closer to the black dashed line (often overlapping it) than the grey dashed line.

It is remarkable how good the fit of this rule of thumb is, considering the amount of complexity captured, as a single line seem to describe well the threshold above which household are needed for accurate predictions for a wide range of R_0 values, irrespective of the values of ϕ, ψ (and hence p_{aa}) and any degree of variation in contact rates between adults and children and in assortativity (at least between random mixing and UK-like contact patterns). Furthermore, with suitable adjustments and taking into account threshold (R_0 close to 1) and saturation (large R_0) effects, a linear relationship broadly extends to very different population structures and different accuracy thresholds.

Unavoidably, there was some degree of variability in choosing the coefficients of the rule of thumb, and slightly different choices would have looked equally satisfactory. In particular, it became quickly apparent that, for every accuracy threshold ε, the coefficients of the rule of thumb could roughly be joined by a straight line, so the final choice in the value of such coefficients also reflected an attempt to show a perfectly clear linear relationship between their values for different populations, as visible in Supplementary Figure 25D.

In an attempt to extrapolate our results even further, we highlight the fact that, in each straight line in Supplementary Figure 25D, the coefficients relative to South Africa fit at 1/4 of the distance between Sierra Leone and Great Britain. This is interesting as South Africa has a fraction of children that also roughly fits at 1/4 of the distance between that of Sierra Leone and that of Great Britain. Therefore we speculate that, when a new population is considered, one could compute the fraction of the children, find its position in between that of the Great Britain and Sierra Leone, and choose the coefficients of the new rule of thumb that sit in the same position along the line plotted in Supplementary Figure 25D for each accuracy threshold. Although untested in this work, this would provide a simple extrapolation rule that could guide model design for any population of interest.

3 Supplementary Tables

Supplementary Table 1: Household composition of Great Britain. The table reports the probability h_{n_a,n_c} that a randomly selected household has n_a adults and n_c children, expressed in %. Zero values are not shown.

		Children									
		0	1	2	3	4	5	6	7	8+	
Adults	0		0.00	0.00	0.00	0.00	0.00	0.00	0.00	0.01	**0.02**
	1	30.28	3.01	2.03	0.70	0.18	0.06	0.01	0.02		**36.29**
	2	31.06	6.91	8.65	2.89	0.74	0.13	0.05			**50.43**
	3	6.58	1.83	0.80	0.26	0.06	0.03				**9.56**
	4	2.14	0.54	0.18	0.05	0.03					**2.95**
	5	0.46	0.09	0.03	0.02						**0.60**
	6	0.08	0.02	0.01							**0.11**
	7	0.02	0.01								**0.02**
	8+	0.01									**0.01**
		**70.62**	**12.40**	**11.71**	**3.93**	**1.02**	**0.22**	**0.06**	**0.02**	**0.01**	**100.00**

Supplementary Table 2: Household structure of Great Britain. Relevant statistics are reported with their symbols and means.

**Distribution**	**Symbol**	**Number of household members**								**Mean**
		1	2	3	4	5	6	7	8+	
Size distribution of a randomly selected household	h_n	30.28	34.07	15.51	13.32	4.88	1.41	0.33	0.20	2.35
Size distribution of the house of a randomly selected individual	π_n	12.89	29.00	19.81	22.67	10.37	3.59	0.97	0.69	3.07
Size distribution of the house of a randomly selected individual, conditional on having at least 2 members	$\tilde{h}_n$	–	48.87	22.25	19.11	6.99	2.02	0.47	0.29	2.94
Size distribution of the household of a randomly selected adult	π_n^a	16.68	35.87	19.59	17.65	7.06	2.18	0.58	0.39	2.73
Size distribution of the household of a randomly selected child	π_n^c	0.01	5.64	20.54	39.77	21.63	8.38	2.30	1.73	4.20

Supplementary Table 3: Household composition of Great Britain by individual. The table reports the probabilities $\pi^a_{n_a,n_c}$ (A) and $\pi^c_{n_a,n_c}$ (B) that the household of a randomly selected adults (A) or child (B) has n_a adults and n_c children, expressed in %. Zero values are not shown.

**A)**		Children									
		0	1	2	3	4	5	6	7	8+	
Adults	0										
	1	16.68	1.66	1.12	0.39	0.10	0.03	0.01	0.01		**19.99**
	2	34.22	7.61	9.53	3.19	0.82	0.15	0.05			**55.55**
	3	10.87	3.03	1.32	0.42	0.11	0.06				**15.80**
	4	4.71	1.19	0.41	0.12	0.07					**6.49**
	5	1.27	0.24	0.09	0.07						**1.66**
	6	0.26	0.05	0.05							**0.36**
	7	0.07	0.02								**0.09**
	8+	0.05									**0.05**
		**68.12**	**13.79**	**12.51**	**4.18**	**1.10**	**0.23**	**0.06**	**0.01**		**100.00**

**B)**		Children									
		0	1	2	3	4	5	6	7	8+	
Adults	0		0.01	0.01	0.01	0.00	0.00	0.00	0.00	0.14	**0.18**
	1		5.63	7.60	3.95	1.37	0.53	0.13	0.25		**19.46**
	2		12.93	32.39	16.25	5.56	1.24	0.56			**68.93**
	3		3.43	3.00	1.43	0.48	0.33				**8.67**
	4		1.01	0.69	0.30	0.25					**2.24**
	5		0.16	0.12	0.14						**0.42**
	6		0.03	0.06							**0.08**
	7		0.01								**0.01**
	8+										
			**23.21**	**43.87**	**22.08**	**7.67**	**2.10**	**0.69**	**0.25**	**0.14**	**100.00**

Supplementary Table 4: Household composition of Sierra Leone: probability h_{n_a,n_c} that a randomly selected household has n_a adults and n_c children, expressed in %. Zero values are not shown.

		Children																
			1	2	3	4	5	6	7	8	9	10	11	12	13	14	15	
Adults			0.11	0.04	0.01	0.02												**0.19**
	1	3.88	1.66	2.22	1.79	1.14	0.64	0.16	0.19	0.02								**11.71**
	2	3.48	6.57	9.67	9.31	7.33	3.89	1.60	0.83	0.43	0.17	0.05	0.01				0.03	**43.37**
	3	1.53	2.32	4.23	4.64	3.55	2.55	1.58	1.29	0.47	0.25	0.13	0.20	0.12	0.04			**22.89**
	4	0.60	1.46	2.47	2.40	1.43	1.70	1.25	0.62	0.49	0.22	0.17	0.07	0.04	0.02			**12.93**
	5	0.18	0.51	0.68	0.72	0.71	0.75	0.41	0.31	0.27	0.17	0.10	0.09	0.01				**4.90**
	6	0.13	0.22	0.39	0.33	0.55	0.22	0.12	0.18	0.10	0.06	0.12	0.05					**2.46**
	7	0.09	0.06	0.07	0.19	0.08	0.08	0.10	0.04	0.04	0.04	0.09						**0.88**
	8		0.03	0.04	0.05	0.08	0.04	0.02	0.03	0.01	0.08							**0.38**
	9		0.01	0.01	0.06		0.02	0.02	0.02	0.03								**0.17**
	10				0.03	0.01	0.01	0.04										**0.09**
	11						0.02											**0.02**
	12																	**0.00**
	13					0.01												**0.01**
		**9.90**	**12.96**	**19.83**	**19.52**	**14.90**	**9.92**	**5.30**	**3.51**	**1.85**	**0.98**	**0.67**	**0.41**	**0.16**	**0.06**	**0.00**	**0.03**	**100.00**

Supplementary Table 5: Household structure of Sierra Leone: some statistics, with symbol and mean.

**Distribution**	**Symbol**	**Number of household members**																	**Mean**
		1	2	3	4	5	6	7	8	9	10	11	12	13	14	15	16	17	
Size distribution of a randomly selected household	h_n	0.04	0.05	0.10	0.14	0.16	0.16	0.11	0.07	0.05	0.05	0.02	0.01	0.01	0.01	0.00	0.00	0.00	5.85
Size distribution of the house of a randomly selected individual	π_n	0.01	0.02	0.05	0.10	0.14	0.16	0.13	0.10	0.08	0.08	0.04	0.03	0.02	0.02	0.01	0.01	0.01	7.24
Size distribution of the house of a randomly selected individual, conditional on having at least 2 members	$\tilde{h}_n$	–	0.02	0.05	0.10	0.14	0.16	0.13	0.10	0.08	0.08	0.04	0.03	0.02	0.02	0.01	0.01	0.01	7.29
Size distribution of the household of a randomly selected adult	π_n^a	0.01	0.03	0.07	0.11	0.15	0.16	0.12	0.09	0.07	0.07	0.03	0.03	0.02	0.01	0.01	0.01	0.01	6.79
Size distribution of the household of a randomly selected child	π_n^c	0.00	0.01	0.04	0.09	0.13	0.16	0.14	0.10	0.09	0.08	0.04	0.03	0.02	0.02	0.01	0.01	0.01	7.63

Supplementary Table 6: Household composition of South Africa: probability h_{n_a,n_c} that a randomly selected household has n_a adults and n_c children, expressed in %. Zero values are not shown.

		Children													
		0	1	2	3	4	5	6	7	8	9	10	11	12	
Adults	0		0.23	0.13	0.10	0.12	0.02	0.02	0.03	0.01					**0.65**
	1	11.47	3.67	4.00	2.75	1.93	0.96	0.49	0.18	0.11	0.05	0.04			**25.63**
	2	11.30	8.06	9.16	5.82	3.38	1.83	0.70	0.36	0.15	0.04	0.03	0.01	0.01	**40.85**
	3	3.86	3.27	3.72	2.65	1.93	0.96	0.44	0.28	0.14	0.13	0.10	0.01		**17.50**
	4	1.61	1.72	1.83	1.46	1.04	0.57	0.32	0.29	0.16	0.03	0.03	0.01	0.01	**9.06**
	5	0.64	0.78	0.65	0.70	0.55	0.27	0.25	0.17	0.04	0.05	0.01			**4.10**
	6	0.21	0.09	0.26	0.27	0.22	0.22	0.08	0.05	0.05	0.02	0.02			**1.48**
	7	0.01	0.05	0.12	0.05	0.09	0.07	0.03	0.02	0.01		0.01			**0.46**
	8	0.01	0.02		0.02	0.02	0.03	0.07	0.02						**0.17**
	9			0.01		0.02	0.00	0.02							**0.04**
	10	0.02			0.01	0.01									**0.03**
	11				0.01			0.01							**0.02**
		**29.13**	**17.89**	**19.87**	**13.82**	**9.31**	**4.93**	**2.42**	**1.40**	**0.66**	**0.32**	**0.22**	**0.03**	**0.02**	**100.00**

Supplementary Table 7: Household structure of South Africa: some statistics, with symbol and mean.

**Distribution**	**Symbol**	**Number of household members**																	**Mean**
		1	2	3	4	5	6	7	8	9	10	11	12	13	14	15	16	17	
Size distribution of a randomly selected household	h_n	0.12	0.15	0.16	0.17	0.14	0.10	0.06	0.04	0.02	0.01	0.01	0.01	0.00	0.00	0.00	0.00	0.00	4.27
Size distribution of the house of a randomly selected individual	π_n	0.03	0.07	0.11	0.16	0.16	0.14	0.11	0.07	0.05	0.03	0.03	0.02	0.01	0.01	0.00	0.00	0.00	5.72
Size distribution of the house of a randomly selected individual, conditional on having at least 2 members	$\tilde{h}_n$	–	0.07	0.12	0.16	0.17	0.14	0.11	0.08	0.05	0.03	0.03	0.02	0.01	0.01	0.00	0.00	0.00	5.86
Size distribution of the household of a randomly selected adult	π_n^a	0.05	0.11	0.14	0.16	0.15	0.12	0.09	0.06	0.04	0.02	0.02	0.01	0.01	0.01	0.00	0.00	0.00	5.17
Size distribution of the household of a randomly selected child	π_n^c	0.00	0.02	0.08	0.15	0.18	0.16	0.13	0.09	0.06	0.04	0.04	0.02	0.01	0.01	0.00	0.00	0.00	6.37

Supplementary Table 8: Parameter estimates for UK contact patterns. The values reported are obtained from the raw data of the POLYMOD study (Mossong et al., 2008). The global assortativity θ_g is defined as the fraction of all the contacts a child has which is made with other children. The within-household assortativity depends on the household composition, so the estimate of mean within-household assortativity θ_h is not used: instead random mixing is assumed in each household.

Number of contacts	From a to			From c to			Assortativity	Symbol
	a	c	**any**	a	c	**any**		
household	1449	764	**2213**	980	735	**1715**	36.2 %	(θ_h)
global	3893	779	**4672**	1320	2433	**3753**	57.9 %	θ_g
**overall**	**5342**	**1543**	**6885**	**2300**	**3168**	**5468**	**50.9 %**	

Average number of contacts	From a to			From c to			Ratios	Symbol
	a	c	**any**	a	c	**any**		
household	2.33	1.23	**3.56**	2.63	1.97	**4.6**	0.78	γ_h
global	6.27	1.25	**7.52**	3.54	6.52	**10.06**	0.75	γ_g
**overall**	**8.6**	**2.48**	**11.09**	**6.17**	**8.49**	**14.66**	**0.76**	γ^A

Supplementary Table 9: Summary of parameter values. Model parameters are described, with their notation and value at baseline, and the range of other values explored (GB: Great Britain, SL: Sierra Leone, SA: South Africa).

**Parameter**	**Symbol**	**Baseline value**	**Main values explored**	**Further exploration**
Basic reproduction number	R_0	2	1.5, 2, 4	1.1-4 (Section 2.4)
Relative infectivity of children VS adults	ϕ	1	1, 1.5, 2	0.5, 1, 2 (Section 2.3.3)
Relative susceptibility of children VS adults	ψ	1-4	1-4	0.25-4 (Section 2.3.3)
Within-household adult-to-adult transmission probability (Section 1.2.3)	p_{aa}	0-0.95	GB: 0-0.95<br>SL: 0-0.475<br>SA: 0-0.63	
Assortativity of children in the community (fraction of all contacts a child has which is made with other children; Section 1.2.2)	θ_g	0.2273	Random: 0.2273 (GB), 0.5381 (SL), 0.4592 (SA)<br>Assortative: 0.58 (all populations)	GB only: 0.4, 0.5 (Section 2.3.1)<br>SL only: 0.7 (not shown)
Ratio of average number of contacts an adult and a child have per day	γ $(=\gamma_h$ $=\gamma_g)$	1	Random: 1<br>Assorative: 0.75	
Tolerance for relative accuracy of simpler models' predictions	ε	5%	1%, 10%	

Supplementary Table 10: Household secondary attack rate (SAR) under random mixing. The SAR is reported, expressed in %, for various values of ϕ, ψ and p_{aa}, and is accompanied by the expected size χ^v of a household infected during the stable exponential growth window. In the random mixing scenario, there is no dependence on R_0.

ψ	$\phi = 1$										χ^v
**4**	0	11	24	38	51	62	73	81	88	92	3.53
**3.5**	0	11	23	36	49	61	72	80	87	91	3.48
**3**	0	10	22	34	47	59	70	79	86	90	3.42
**2.5**	0	9	20	32	44	57	68	78	85	90	3.36
**2**	0	9	19	30	42	54	66	76	84	89	3.28
**1.5**	0	8	17	27	39	50	62	73	82	87	3.18
**1**	0	8	16	25	35	46	57	69	79	86	3.07
	$\phi = 1.5$										χ^v
**4**	0	15	31	46	59	70	78	85	90	92	3.53
**3.5**	0	14	29	44	57	68	77	84	89	91	3.48
**3**	0	12	27	41	55	67	76	83	88	91	3.42
**2.5**	0	11	24	38	52	64	74	82	87	90	3.36
**2**	0	10	22	35	48	61	72	80	86	89	3.28
**1.5**	0	9	20	31	44	56	68	77	84	88	3.18
**1**	0	8	18	28	39	50	62	73	81	86	3.07
	$\phi = 2$										χ^v
**4**	0	18	36	52	64	74	81	87	90	92	3.53
**3.5**	0	16	34	50	63	73	80	86	90	91	3.48
**3**	0	15	31	47	60	71	79	85	89	91	3.42
**2.5**	0	13	28	43	57	68	77	84	88	90	3.36
**2**	0	12	25	39	53	65	75	82	87	89	3.28
**1.5**	0	10	22	35	48	60	71	79	85	88	3.18
**1**	0	9	19	30	42	53	65	74	82	86	3.07
p_{aa}	**0**	**.1**	**.2**	**.3**	**.4**	**.5**	**.6**	**.7**	**.8**	**.9**	

Supplementary Table 11: Fraction of within-household transmission under random mixing. The average fraction F_h of total transmission that occurs in households, expressed as %, is reported for various values of R_0, ϕ, ψ and p_{aa}.

ψ	$R_0 = 1.5$ – $\phi = 1$										$R_0 = 2$ – $\phi = 1$										$R_0 = 4$ – $\phi = 1$									
**4**	0	16	28	38	45	51	55	58	60	62	0	12	22	30	37	42	46	49	52	54	0	6	12	17	21	25	28	31	34	36
**3.5**	0	14	27	36	44	49	54	57	59	61	0	11	21	29	35	41	45	48	51	53	0	6	11	16	20	24	27	30	33	36
**3**	0	13	25	34	42	48	53	56	59	60	0	10	19	27	34	39	44	48	50	53	0	5	10	15	19	23	26	29	32	35
**2.5**	0	12	23	32	40	46	51	55	58	60	0	9	18	25	32	38	42	46	50	52	0	5	9	14	18	21	25	28	31	34
**2**	0	11	21	30	37	44	49	53	57	59	0	8	16	23	30	35	41	45	48	51	0	4	8	12	16	20	24	27	30	33
**1.5**	0	9	18	27	34	41	47	51	55	58	0	7	14	21	27	33	38	43	47	50	0	4	7	11	15	18	22	25	29	32
**1**	0	8	16	24	30	37	43	48	52	56	0	6	12	18	24	29	35	40	44	48	0	3	6	10	13	16	20	23	27	30
	$R_0 = 1.5$ – $\phi = 1.5$										$R_0 = 2$ – $\phi = 1.5$										$R_0 = 4$ – $\phi = 1.5$									
**4**	0	20	34	44	50	53	56	58	60	60	0	15	27	35	41	45	48	50	52	53	0	8	15	20	24	27	30	32	34	36
**3.5**	0	19	32	42	48	53	56	58	59	60	0	14	25	34	40	44	47	50	52	53	0	7	14	19	23	27	30	32	34	36
**3**	0	17	30	40	47	52	55	57	59	60	0	13	24	32	38	43	47	49	51	53	0	7	13	18	22	26	29	31	33	35
**2.5**	0	15	28	38	45	50	54	57	59	60	0	12	22	30	36	42	45	48	51	52	0	6	11	16	21	24	28	31	33	35
**2**	0	13	25	35	42	48	53	56	58	59	0	10	19	27	34	40	44	47	50	52	0	5	10	15	19	23	26	29	32	34
**1.5**	0	11	22	31	39	45	50	54	57	59	0	9	17	24	31	37	42	46	49	51	0	4	9	13	17	21	24	28	31	34
**1**	0	9	18	27	34	41	47	51	55	58	0	7	14	21	27	33	38	43	47	50	0	4	7	11	15	18	22	25	29	32
	$R_0 = 1.5$ – $\phi = 2$										$R_0 = 2$ – $\phi = 2$										$R_0 = 4$ – $\phi = 2$									
**4**	0	24	39	47	52	55	57	58	59	60	0	19	31	38	43	46	49	50	51	52	0	10	17	22	26	29	31	33	34	35
**3.5**	0	22	37	45	51	54	56	58	59	60	0	17	29	37	42	46	48	50	51	52	0	9	16	21	25	28	31	32	34	35
**3**	0	20	34	44	50	53	56	58	59	60	0	16	27	35	41	45	48	50	51	52	0	8	15	20	24	27	30	32	34	35
**2.5**	0	18	32	41	48	52	55	57	59	60	0	14	25	33	39	44	47	49	51	52	0	7	13	19	23	26	29	32	34	35
**2**	0	16	29	38	46	51	54	57	58	60	0	12	22	31	37	42	46	49	51	52	0	6	12	17	21	25	28	31	33	35
**1.5**	0	13	25	35	42	48	53	56	58	59	0	10	19	27	34	40	44	47	50	52	0	5	10	15	19	23	26	29	32	34
**1**	0	11	21	30	37	44	49	53	57	59	0	8	16	23	30	35	41	45	48	51	0	4	8	12	16	20	24	27	30	33
p_{aa}	**0**	**.1**	**.2**	**.3**	**.4**	**.5**	**.6**	**.7**	**.8**	**.9**	**0**	**.1**	**.2**	**.3**	**.4**	**.5**	**.6**	**.7**	**.8**	**.9**	**0**	**.1**	**.2**	**.3**	**.4**	**.5**	**.6**	**.7**	**.8**	**.9**

Supplementary Table 12: Household secondary attack rate (SAR) under UK-like assortative contact patterns. The SAR is reported, expressed in %, for various values of R_0, ϕ, ψ and p_{aa}, and is accompanied by the expected size χ^v of a household infected during the stable exponential growth window. Note how the average size of an infected household can be significantly larger than the average size of a randomly selected one or even of the household of a randomly selected individual.

ψ	$R_0 = 1.5$ – $\phi = 1$										χ^v
**4**	0	11	24	37	50	61	71	80	88	93	3.82
**3.5**	0	10	22	35	47	59	70	79	87	93	3.78
**3**	0	9	20	32	45	57	68	78	86	92	3.72
**2.5**	0	9	19	30	42	54	66	76	85	91	3.65
**2**	0	8	17	27	38	50	62	74	83	90	3.55
**1.5**	0	7	15	24	34	45	57	69	81	89	3.42
**1**	0	6	13	21	30	39	51	63	75	85	3.23
	$R_0 = 1.5$ – $\phi = 1.5$										χ^v
**4**	0	16	33	48	61	71	79	86	92	95	3.86
**3.5**	0	15	31	46	59	70	79	86	91	95	3.82
**3**	0	13	28	43	56	68	77	85	90	94	3.77
**2.5**	0	12	25	39	53	65	75	83	89	93	3.71
**2**	0	10	22	35	48	61	72	81	88	92	3.62
**1.5**	0	9	19	30	42	55	67	77	85	90	3.49
**1**	0	7	15	25	35	46	58	70	80	88	3.31
	$R_0 = 1.5$ – $\phi = 2$										χ^v
**4**	0	21	40	56	68	77	84	90	94	96	3.89
**3.5**	0	19	38	54	67	76	84	89	93	95	3.85
**3**	0	17	35	51	64	75	82	88	92	95	3.80
**2.5**	0	15	32	47	61	72	81	87	91	94	3.74
**2**	0	13	28	43	56	68	78	85	90	93	3.66
**1.5**	0	11	23	37	50	62	73	81	87	91	3.54
**1**	0	9	18	29	41	53	64	74	83	89	3.36
p_{aa}	**0**	**.1**	**.2**	**.3**	**.4**	**.5**	**.6**	**.7**	**.8**	**.9**	

ψ	$R_0 = 2$ – $\phi = 1$										χ^v
**4**	0	11	24	37	50	61	71	80	88	94	3.84
**3.5**	0	10	22	35	48	59	70	79	87	93	3.79
**3**	0	9	20	33	45	57	68	78	86	92	3.74
**2.5**	0	9	19	30	42	54	66	76	85	92	3.66
**2**	0	8	17	27	38	50	62	74	84	90	3.56
**1.5**	0	7	15	24	34	45	57	70	81	89	3.43
**1**	0	6	13	21	30	39	51	63	75	85	3.23
	$R_0 = 2$ – $\phi = 1.5$										χ^v
**4**	0	16	33	48	61	71	80	87	92	95	3.87
**3.5**	0	15	31	46	59	70	79	86	91	95	3.83
**3**	0	13	28	43	56	68	77	85	91	94	3.78
**2.5**	0	12	25	39	53	65	75	84	90	93	3.72
**2**	0	10	22	35	48	61	72	81	88	92	3.63
**1.5**	0	9	19	30	43	55	67	77	85	91	3.50
**1**	0	7	16	25	35	46	58	70	80	88	3.31
	$R_0 = 2$ – $\phi = 2$										χ^v
**4**	0	21	40	56	68	78	85	90	94	96	3.90
**3.5**	0	19	38	54	67	76	84	89	93	95	3.86
**3**	0	17	35	51	64	75	83	88	93	95	3.81
**2.5**	0	15	32	48	61	72	81	87	91	94	3.75
**2**	0	13	28	43	57	68	78	85	90	93	3.67
**1.5**	0	11	23	37	50	62	73	81	88	91	3.55
**1**	0	9	18	29	41	53	64	74	83	89	3.37
p_{aa}	**0**	**.1**	**.2**	**.3**	**.4**	**.5**	**.6**	**.7**	**.8**	**.9**	

ψ	$R_0 = 4$ – $\phi = 1$										χ^v
**4**	0	11	24	37	50	61	71	80	88	94	3.87
**3.5**	0	10	22	35	48	59	70	79	88	94	3.83
**3**	0	9	20	33	45	57	68	78	87	93	3.77
**2.5**	0	9	19	30	42	54	66	77	86	92	3.69
**2**	0	8	17	27	38	50	62	74	84	91	3.59
**1.5**	0	7	15	24	34	45	57	70	81	89	3.44
**1**	0	6	13	21	29	39	51	63	75	85	3.23
	$R_0 = 4$ – $\phi = 1.5$										χ^v
**4**	0	16	33	48	61	72	80	87	92	96	3.90
**3.5**	0	15	31	46	59	70	79	86	92	95	3.86
**3**	0	13	28	43	57	68	78	85	91	95	3.81
**2.5**	0	12	25	40	53	65	76	84	90	94	3.75
**2**	0	10	22	35	49	61	73	82	88	93	3.66
**1.5**	0	9	19	30	43	55	67	78	86	91	3.52
**1**	0	7	16	25	35	46	58	70	80	88	3.32
	$R_0 = 4$ – $\phi = 2$										χ^v
**4**	0	21	41	57	69	78	85	90	94	96	3.92
**3.5**	0	19	38	54	67	77	84	90	94	96	3.88
**3**	0	17	35	51	65	75	83	89	93	95	3.84
**2.5**	0	15	32	48	62	73	81	87	92	94	3.78
**2**	0	13	28	43	57	69	78	85	90	93	3.69
**1.5**	0	11	23	37	50	63	73	82	88	92	3.57
**1**	0	9	18	29	41	53	64	75	83	89	3.37
p_{aa}	**0**	**.1**	**.2**	**.3**	**.4**	**.5**	**.6**	**.7**	**.8**	**.9**	

Supplementary Table 13: Fraction of within-household transmission under UK-like contact patterns. The average fraction F_h of total transmission that occurs in households, expressed as %, is reported for various values of R_0, ϕ, ψ and p_{aa}.

ψ	$R_0 = 1.5$ – $\phi = 1$									
**4**	0	16	28	37	43	47	50	52	54	55
**3.5**	0	14	26	35	42	47	50	52	54	56
**3**	0	13	24	33	40	46	49	52	54	56
**2.5**	0	12	22	31	38	44	49	52	55	57
**2**	0	10	20	28	36	42	48	52	55	57
**1.5**	0	9	17	25	33	40	46	51	55	58
**1**	0	7	14	22	28	35	41	47	53	57
	$R_0 = 1.5$ – $\phi = 1.5$									
**4**	0	21	34	42	46	49	51	52	53	53
**3.5**	0	19	32	40	45	49	51	52	53	53
**3**	0	17	30	39	44	48	50	52	53	54
**2.5**	0	15	27	36	43	47	50	52	54	55
**2**	0	13	24	33	41	46	50	52	54	55
**1.5**	0	11	21	30	37	44	48	52	55	57
**1**	0	9	17	25	33	40	46	51	55	58
	$R_0 = 1.5$ – $\phi = 2$									
**4**	0	25	38	44	47	49	50	51	52	52
**3.5**	0	23	36	43	47	49	50	51	52	52
**3**	0	21	34	42	46	49	51	52	52	53
**2.5**	0	18	31	40	45	48	51	52	53	53
**2**	0	16	28	37	43	48	50	52	53	54
**1.5**	0	13	24	33	41	46	50	52	54	55
**1**	0	10	20	28	36	42	48	52	55	57
p_{aa}	**0**	**.1**	**.2**	**.3**	**.4**	**.5**	**.6**	**.7**	**.8**	**.9**

ψ	$R_0 = 2$ – $\phi = 1$									
**4**	0	12	22	29	35	39	42	44	46	48
**3.5**	0	11	20	28	34	38	42	44	46	48
**3**	0	10	19	26	32	37	41	44	46	48
**2.5**	0	9	17	24	31	36	40	44	47	49
**2**	0	8	15	22	28	34	39	43	47	49
**1.5**	0	7	13	20	26	32	37	42	46	50
**1**	0	6	11	17	22	28	33	39	44	49
	$R_0 = 2$ – $\phi = 1.5$									
**4**	0	16	27	34	38	41	43	44	45	46
**3.5**	0	15	25	32	37	40	43	44	45	46
**3**	0	13	23	31	36	40	42	44	46	46
**2.5**	0	12	21	29	35	39	42	44	46	47
**2**	0	10	19	26	33	37	41	44	46	48
**1.5**	0	8	16	23	30	35	40	44	47	49
**1**	0	7	13	20	26	32	37	42	46	50
	$R_0 = 2$ – $\phi = 2$									
**4**	0	19	30	36	40	41	43	44	44	45
**3.5**	0	18	29	35	39	41	43	44	44	45
**3**	0	16	27	34	38	41	43	44	45	45
**2.5**	0	14	25	32	37	40	43	44	45	46
**2**	0	12	22	30	35	39	42	44	46	47
**1.5**	0	10	19	26	33	37	41	44	46	48
**1**	0	8	15	22	28	34	39	43	47	49
p_{aa}	**0**	**.1**	**.2**	**.3**	**.4**	**.5**	**.6**	**.7**	**.8**	**.9**

ψ	$R_0 = 4$ – $\phi = 1$									
**4**	0	6	12	16	20	23	25	27	29	31
**3.5**	0	6	11	15	19	22	25	27	29	31
**3**	0	5	10	14	18	21	24	27	29	31
**2.5**	0	5	9	13	17	20	24	27	29	31
**2**	0	4	8	12	15	19	23	26	29	32
**1.5**	0	3	7	10	14	18	21	25	28	32
**1**	0	3	6	9	12	15	19	22	26	31
	$R_0 = 4$ – $\phi = 1.5$									
**4**	0	8	15	19	22	25	26	28	28	29
**3.5**	0	8	14	18	22	24	26	28	29	30
**3**	0	7	12	17	21	24	26	27	29	30
**2.5**	0	6	11	16	20	23	25	27	29	30
**2**	0	5	10	14	18	22	24	27	29	31
**1.5**	0	4	8	12	16	20	23	26	29	31
**1**	0	3	7	10	14	18	21	25	28	32
	$R_0 = 4$ – $\phi = 2$									
**4**	0	10	17	21	24	25	27	27	28	28
**3.5**	0	9	16	20	23	25	27	27	28	29
**3**	0	8	15	19	22	25	26	28	28	29
**2.5**	0	7	13	18	21	24	26	28	29	29
**2**	0	6	12	16	20	23	25	27	29	30
**1.5**	0	5	10	14	18	22	24	27	29	31
**1**	0	4	8	12	15	19	23	26	29	32
p_{aa}	**0**	**.1**	**.2**	**.3**	**.4**	**.5**	**.6**	**.7**	**.8**	**.9**

4 Supplementary Figures

Supplementary Figure 1: Relationship between contact rates as elements of the NGM. If T is chosen as the time of recovery, the fraction ζ of all contacts during the entire infectious period that are infectious contacts (see Supplementary Methods, Section 1.1.5) is given by the ratio of the area under the red curve and the area of the rectangle of height c and width T. If the recovery time is arbitrarily chosen to be T' instead, with $T' = 2T$ say, then ζ is halved, so that the choice of whether in some time intervals individuals are considered infectious but with zero infectivity or already recovered bears no influence on the relationship between contact rates and elements of the NGM.

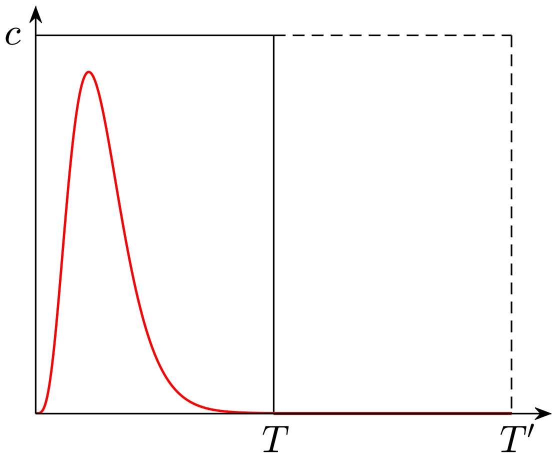

Supplementary Figure 2: Relationship between different parameterisations of within-household transmission. Model AH can be parameterised in terms of the within-household infectivity β_h or in terms of the within-household infectious-adult-to-susceptible-adult transmission probability p_{aa}. The dash-dotted lines refer to the values of $p_n = 1 - \exp(-\beta_h/(n-1))$, for $n = 2, 3, \ldots, 8$ (from top to bottom), and the continuous line is the average taken over the distribution $\left\{\tilde{h}_n\right\}$. Note the monotonic (and thus invertible) relationship.

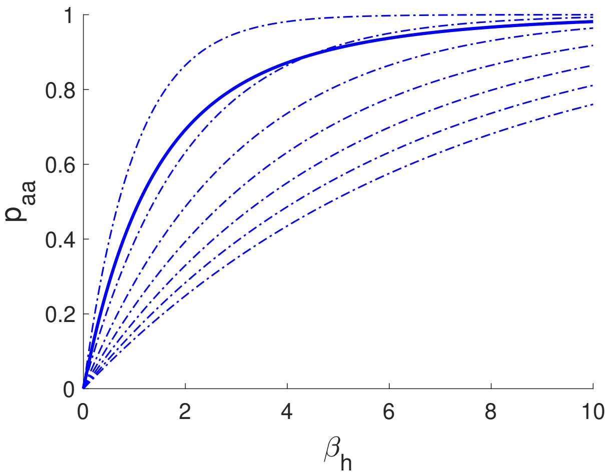

Supplementary Figure 3: Mapping assortativity from model AH to model A. A visual exploration provides insight into the conditions under which the mapping procedure estimating the assortativity parameter θ^{A} for model A from the age-stratified incidence vector v^{AH} fails. The figure shows the fractions of adults (blue) and children (red) in the incidence predicted by model AH, i.e. the components of v^{AH} (dashed horizontal lines) and those predicted by model A, i.e. the components of v^{A} (continuous lines) as a function of the assortativity θ^{A}, for different values of ψ. The mapping procedure attempts to compute the assortativity θ^{A} as the point at which the continuous and dashed lines intersect, if any. Parameters used in this example are $R_0 = 2$, $\phi = 1$, $p_{aa} = 0.5$ and baseline random mixing: γ ($= \gamma_h = \gamma_g$) $= 1$ and $\theta_g = 0.2273$). Note how a valid assortativity θ^{A} can be found for very low values of ψ but then, for increasing ψ, the mapping first returns a negative (i.e. unacceptable) value of θ^{A} (e.g. for $\psi = 0.8$), then no value at all (e.g. for $\psi = 0.99$ or $\psi = 1$) and then again a valid solution for $\psi > 1$.

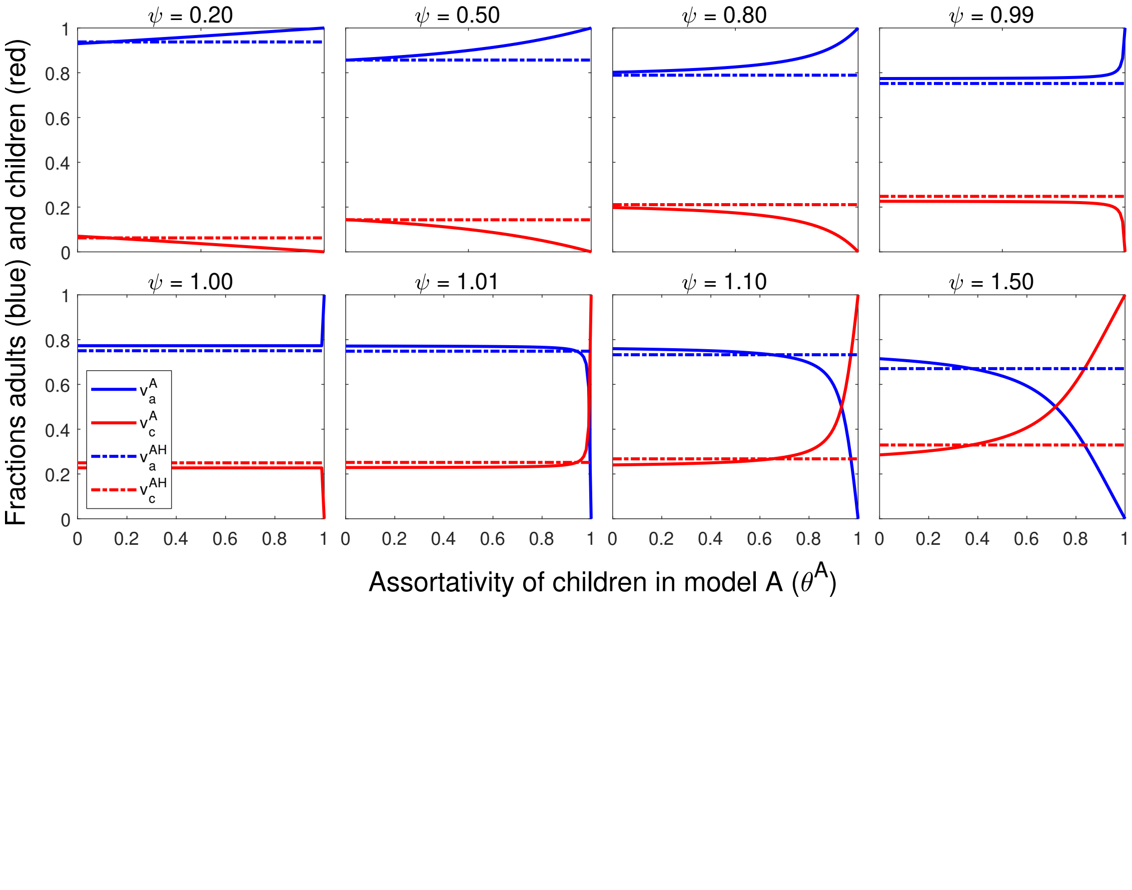

Supplementary Figure 4: Early epidemic indicators for model AH with random mixing. A) Fraction of incidence consisting of children (i.e. component v_c of v^{AH}); B) fraction of incidence of new household primary cases consisting of children (i.e. component v_h^c of v_h^{AH} – note how it depends only on ψ when random mixing is assumed); C) SAR; and D) fraction F_h of total transmission that occurs in household. All indicators are expressed in % and are plotted as functions of p_{aa} and ψ for various values of R_0 and ϕ, for the population of Great Britain and assuming random mixing: $\gamma\ (= \gamma_h = \gamma_g) = 1$, $\theta_g = 0.2273$.

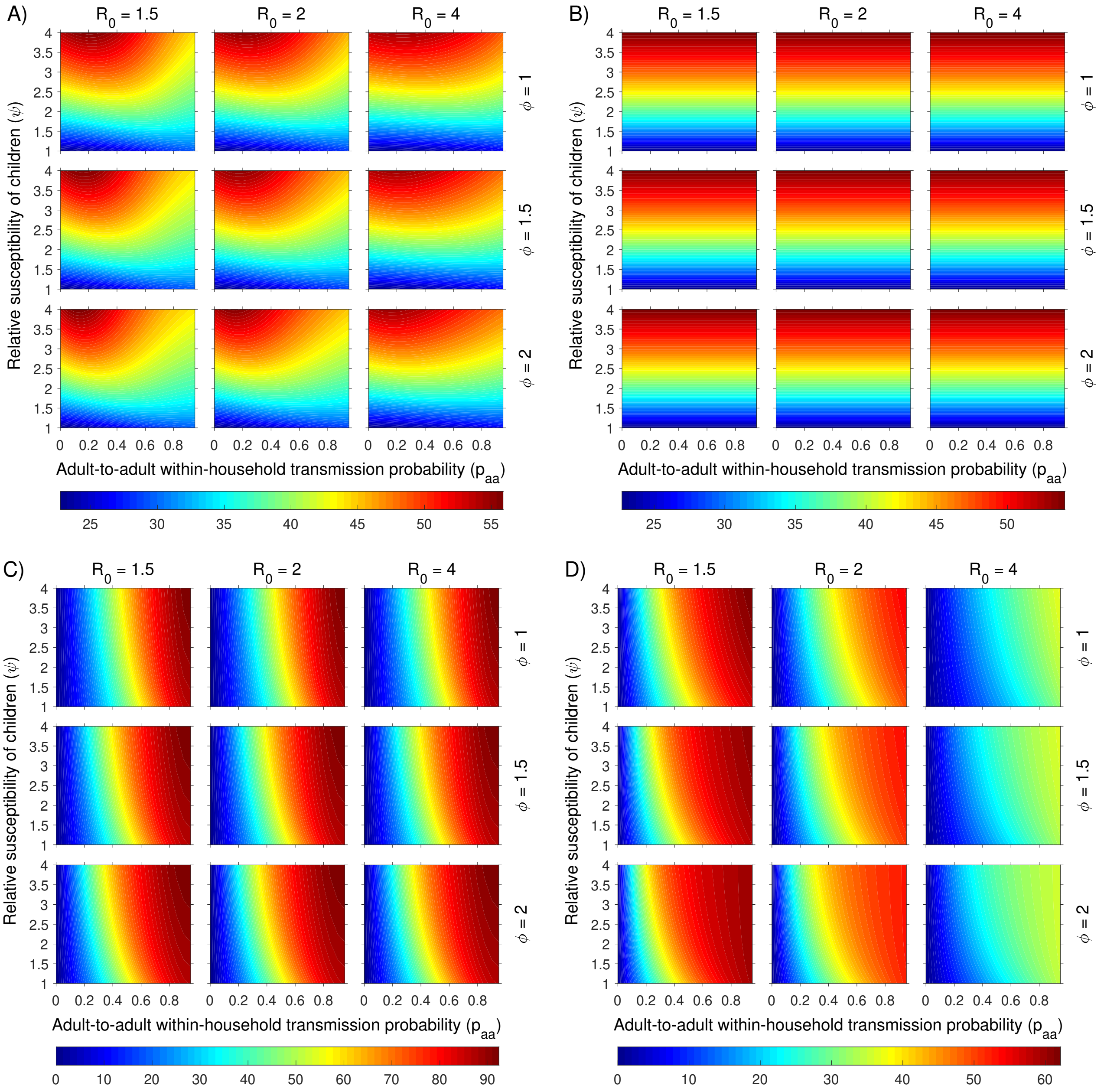

Supplementary Figure 5: Model comparison in terms of final size and peak incidence with random mixing. Average epidemic final size (top) and peak daily incidence (bottom), measured in % of population, as predicted by models AH, A and H, and comparison by simplest model acceptance regions plot, for $R_0 = 1.5$ (left: a-d, m-p), $R_0 = 2$ (middle: e-h, q-t) and $R_0 = 4$ (right: i-l, u-x), as a function of p_{aa} and ψ, for the population of Great Britain. Contact patterns assume random mixing: $\gamma = 1$, $\theta_g = 0.2273$. Other parameters: $\phi = 1$ and $\varepsilon = 5\%$.

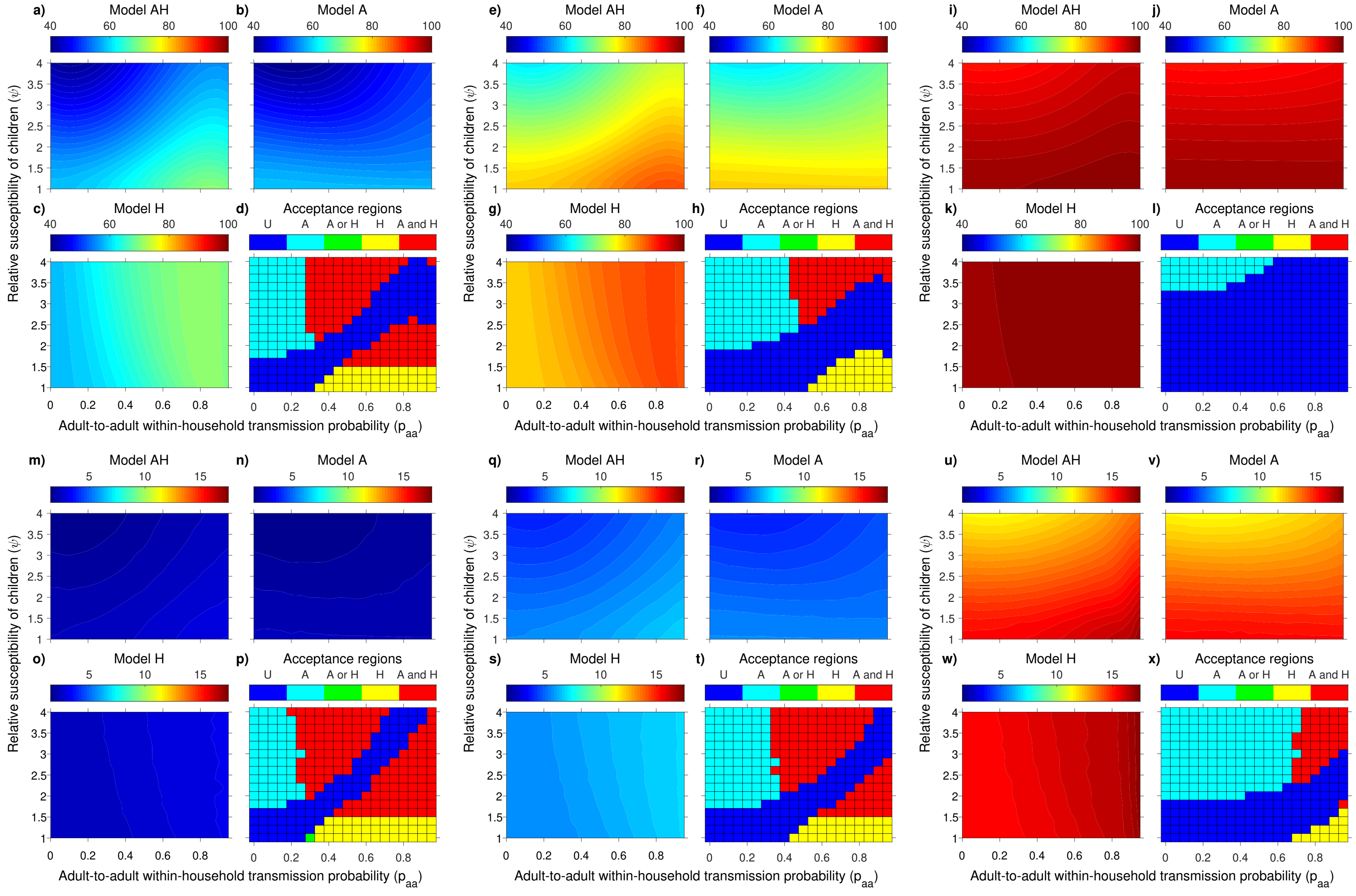

Supplementary Figure 6: Model comparison in terms of time to the peak and age-stratified final size with random mixing. Average time to peak daily incidence (top), measured in mean number of generations, as predicted by models AH, A and H, and comparison by simplest model acceptance regions plot, and average final size among adults and children (bottom), measured in % of population, as predicted by models AH and A, for $R_0 = 1.5$ (left: a-d, m-p), $R_0 = 2$ (middle: e-h, q-t) and $R_0 = 4$ (right: i-l, u-x), as a function of p_{aa} and ψ, for the population of Great Britain. Contact patterns assume random mixing: $\gamma = 1$, $\theta_g = 0.2273$. Other parameters: $\phi = 1$ and $\varepsilon = 5\%$.

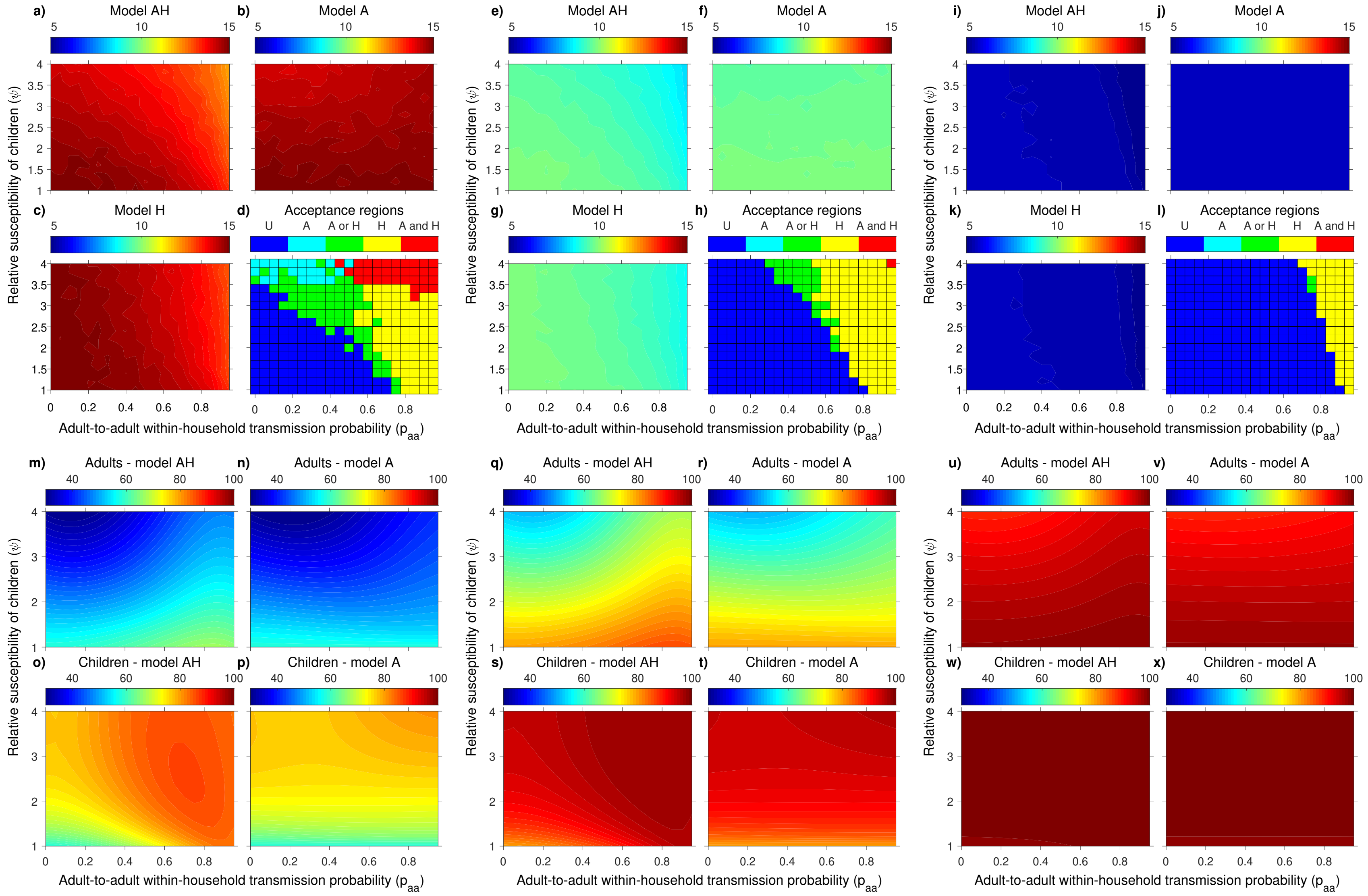

Supplementary Figure 7: Simplest model acceptance regions, based on each output separately and all three together, with random mixing. The simplest model that accurately captures each output or all three simultaneously (intersection) is identified over the (p_{aa}, ψ)-space for $R_0 = 1.5$ (left: a-d, m-p), $R_0 = 2$ (middle: e-h, q-t) and $R_0 = 4$ (right: i-l, u-x) and for $\phi = 1$ (top: a-l) and $\phi = 2$ (bottom: m-x), for the population of Great Britain. Contact patterns assume random mixing: $\gamma = 1$, $\theta_g = 0.2273$. Other parameters: $\varepsilon = 5\%$.

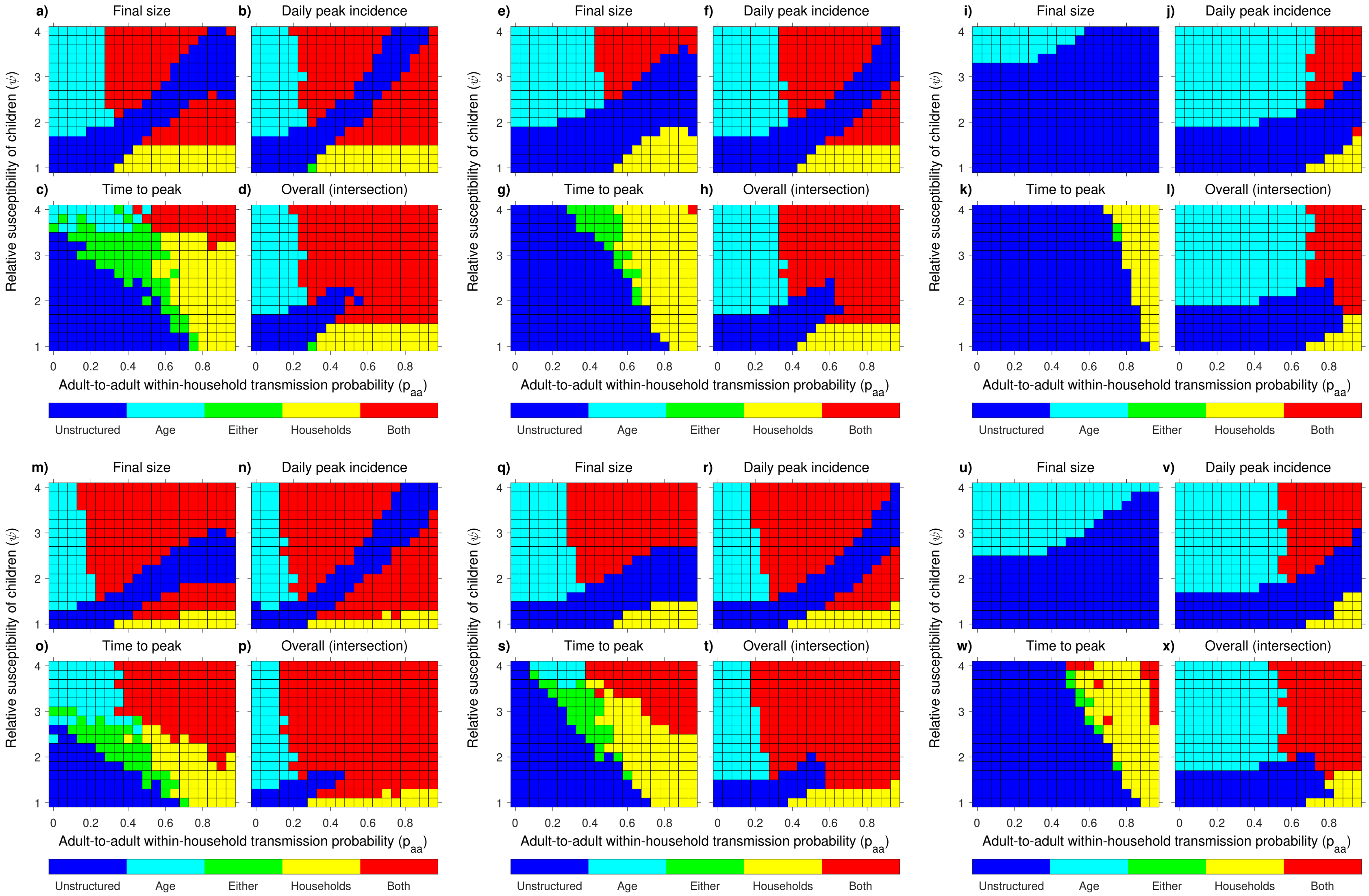

Supplementary Figure 8: Simplest model acceptance regions, based on each output separately and all three together, with random mixing. The simplest model that accurately captures each output or all three simultaneously (intersection) is identified over the (p_{aa}, ψ)-space for $R_0 = 1.5$ (left: a-d, m-p), $R_0 = 2$ (middle: e-h, q-t) and $R_0 = 4$ (right: i-l, u-x) and for $\varepsilon = 1\%$ (top: a-l) and $\varepsilon = 10\%$ (bottom: m-x), for the population of Great Britain. Contact patterns assume random mixing: $\gamma = 1$, $\theta_g = 0.2273$. Other parameters: $\phi = 1$.

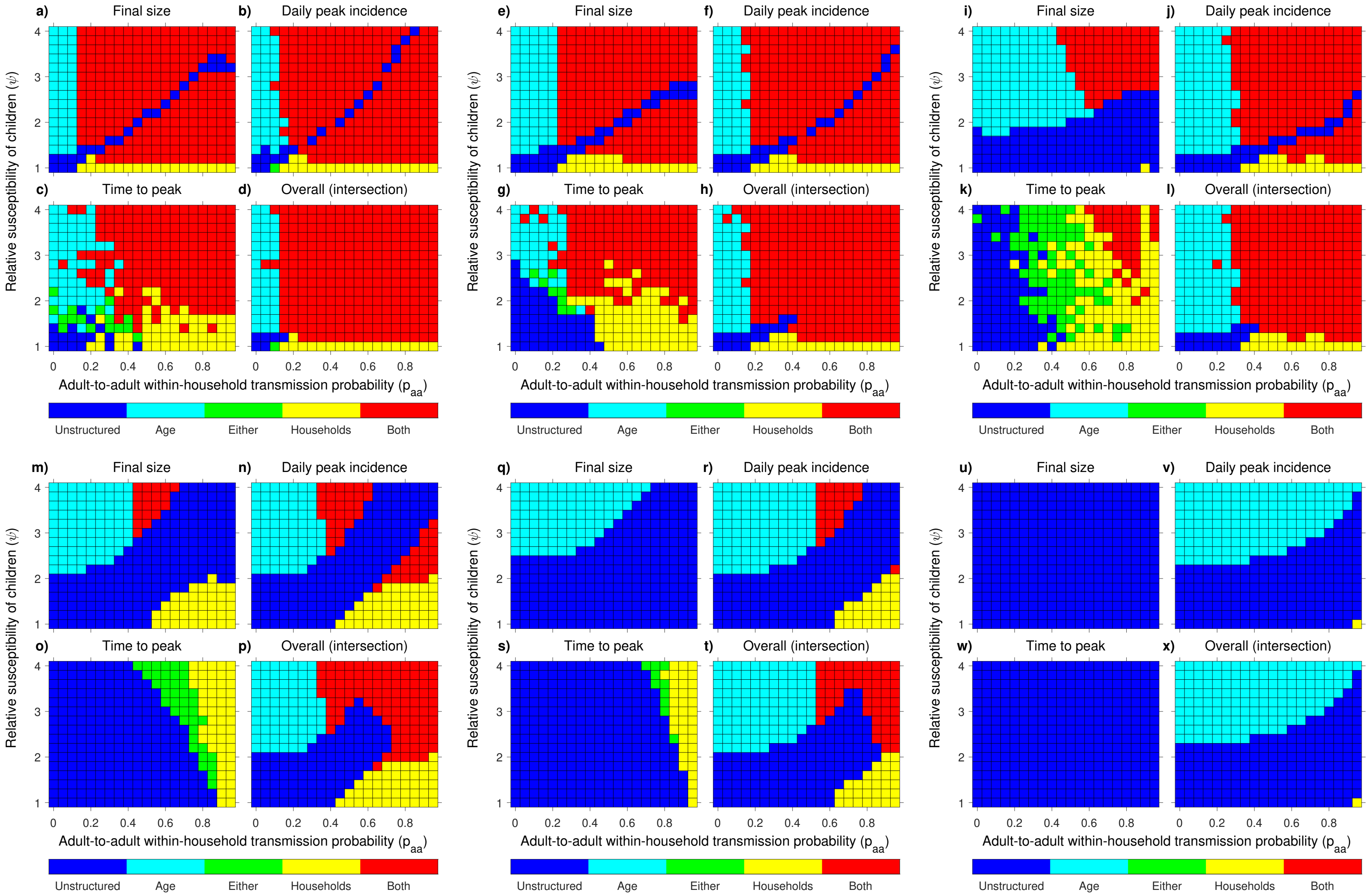

Supplementary Figure 9: Sensitivity analysis on overall simplest model acceptance regions with random mixing. Overall simplest model acceptance regions and superimposed SAR contour levels are plotted for various values of R_0 and ϕ, with rejection thresholds $\varepsilon = 1\%$ (A), $\varepsilon = 5\%$ (B; same regions as in Figure 3A of main text) and $\varepsilon = 10\%$ (C), for the population of Great Britain. Contact patterns assume random mixing: $\gamma = 1$, $\theta_g = 0.2273$.

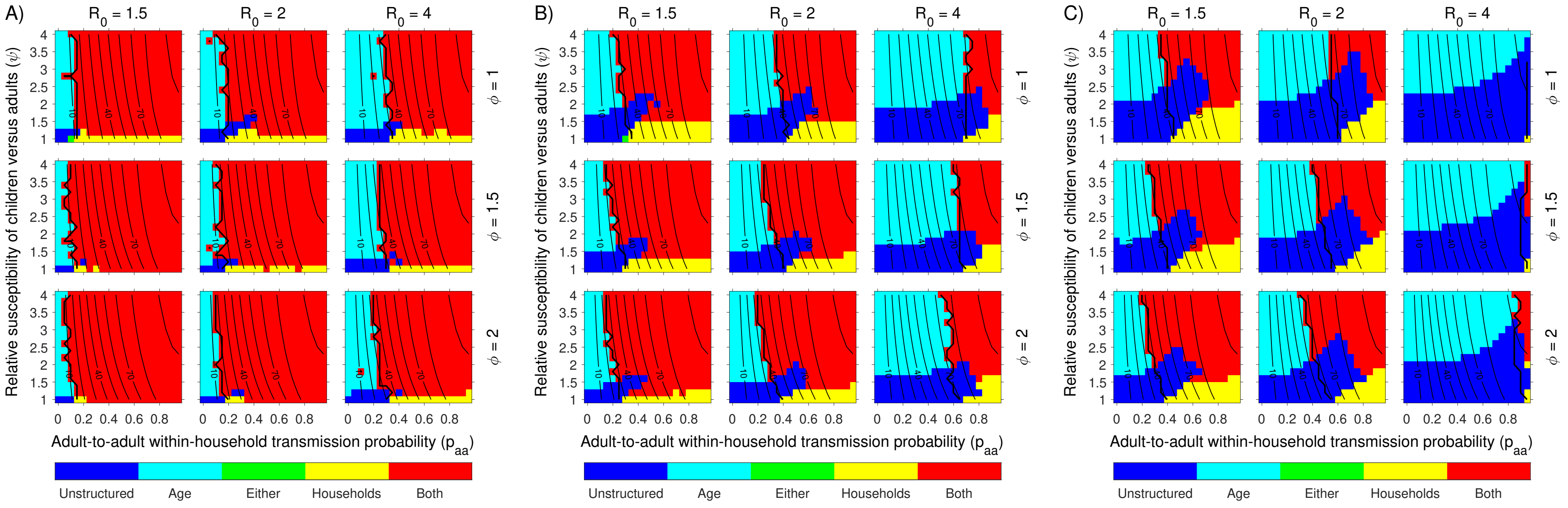

Supplementary Figure 10: Mapped assortativity of model A with random mixing and UK-like contact patterns. Global assortativity θ^{A} estimated for model A from the model mapping procedure, with random mixing (A: $\gamma = 1$, $\theta_g = 0.2273$) and UK-like contact patterns including assortative mixing (B: $\gamma = 0.75$, $\theta_g = 0.58$) as a function of p_{aa} and ψ for various values of R_0 and ϕ, for the population of Great Britain. The dashed line (also along the y-axis) shows where the assortativity has not changed when households are removed ($\theta^{\mathrm{A}} = \theta_g$). Note the limited impact of R_0 and ϕ, and the fact that the presence of households introduces an apparent assortative mixing that is generally higher than random but lower than what measured in UK-like contact patterns. Only the region for $\psi > 1$ is plotted, as no value of θ^{A} can be found for $\psi = 1$ when random mixing is assumed (see Supplementary Methods, Section 1.3, and Supplementary Discussion, Section 2.3.3).

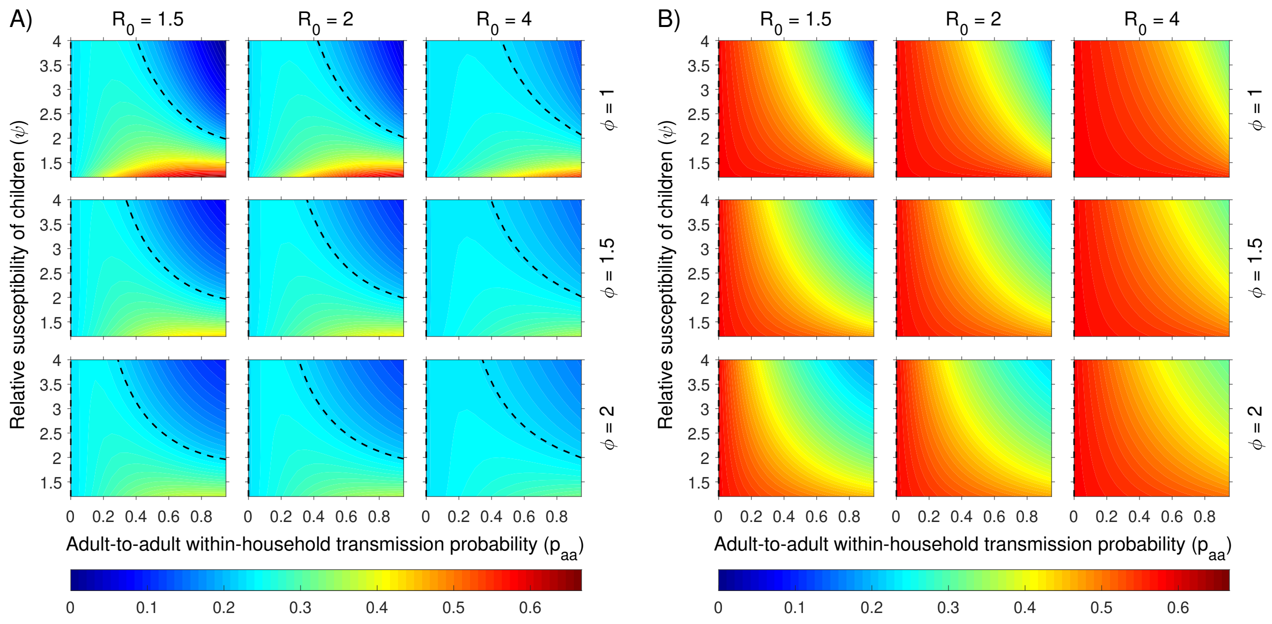

Supplementary Figure 11: Early epidemic indicators for model AH with UK-like contact patterns. A) Fraction of incidence consisting of children (i.e. component v_c of v^{AH}); B) fraction of incidence of new household primary cases consisting of children (i.e. component v_h^c of v_h^{AH}); C) SAR; and D) fraction F_h of total transmission that occurs in household. All indicators are expressed in % and are plotted as functions of p_{aa} and ψ for various values of R_0 and ϕ, for the population of Great Britain and assuming UK-like contact patterns: $\gamma = 0.75$, $\theta_g = 0.58$.

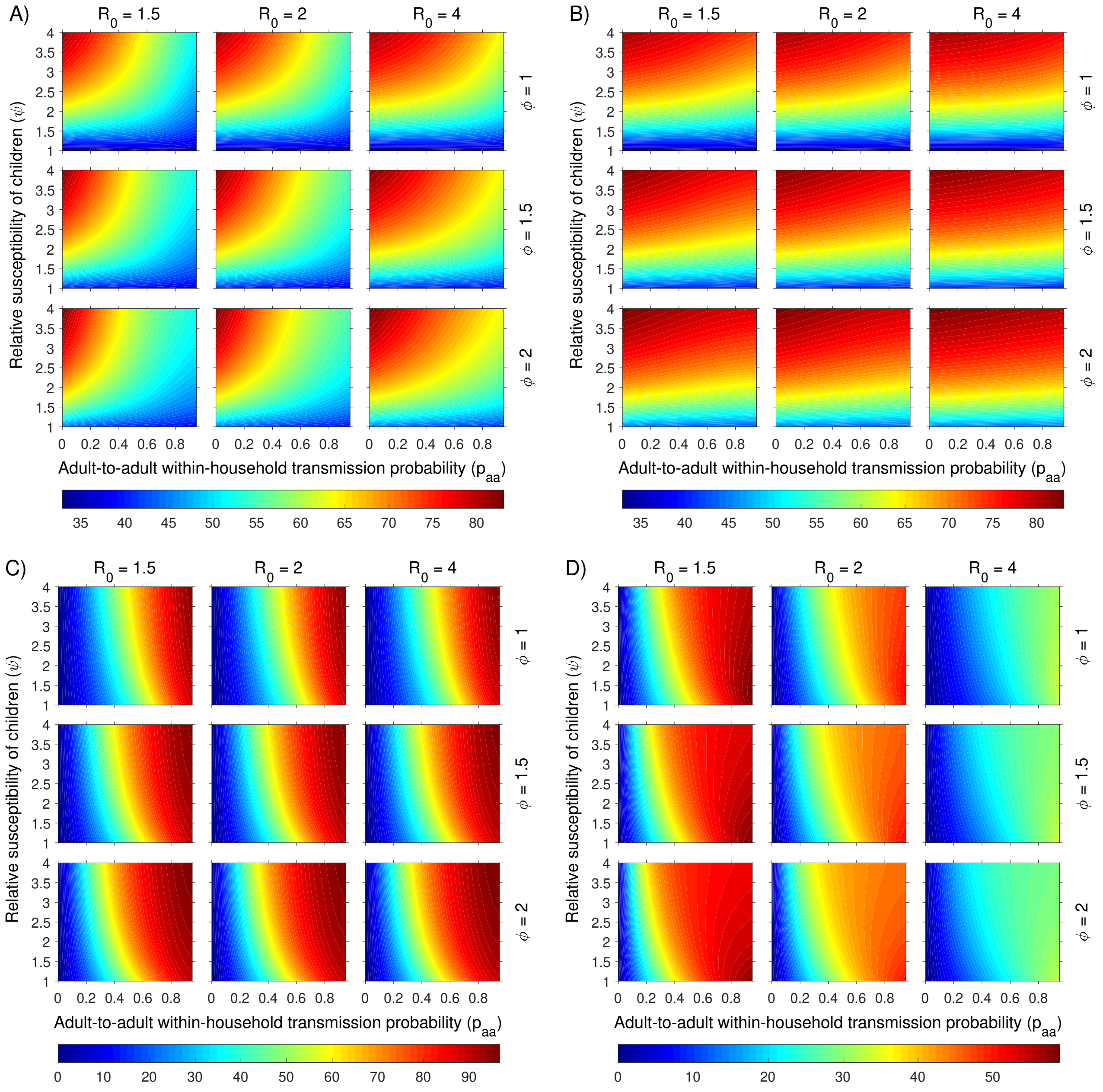

Supplementary Figure 12: Model comparison in terms of final size and peak incidence with UK-like contact patterns. Average epidemic final size (top) and peak daily incidence (bottom), measured in % of population, as predicted by models AH, A and H, and comparison by simplest model acceptance regions plot, for $R_0 = 1.5$ (left: a-d, m-p), $R_0 = 2$ (middle: e-h, q-t) and $R_0 = 4$ (right: i-l, u-x), as a function of p_{aa} and ψ, for the population of Great Britain. UK-like contact patterns are assumed: $\gamma = 0.75$, $\theta_g = 0.58$. Other parameters: $\phi = 1$ and $\varepsilon = 5\%$.

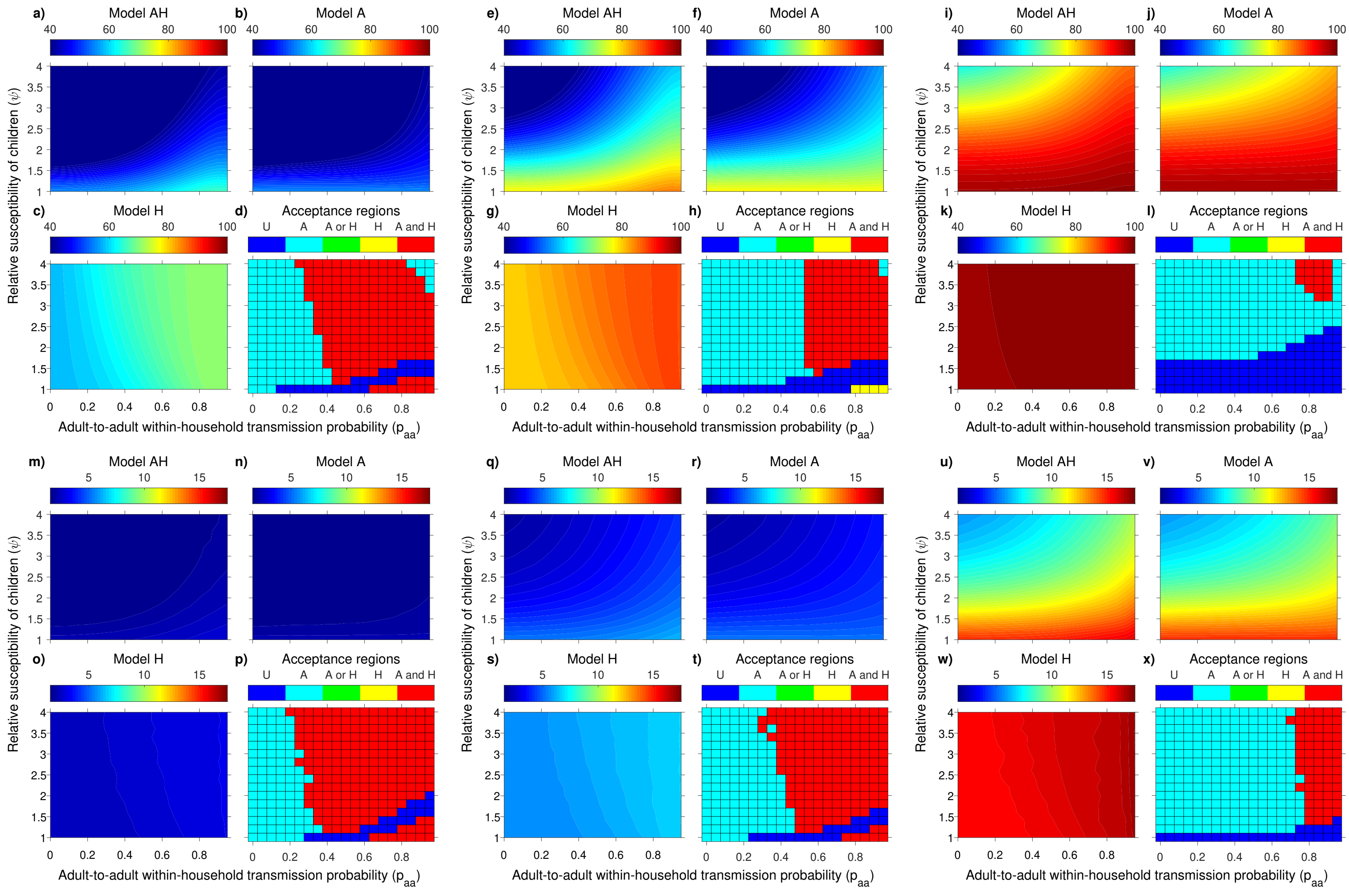

Supplementary Figure 13: Model comparison in terms of time to the peak and age-stratified final size with UK-like contact patterns. Average time to peak daily incidence (top), measured in mean number of generations, as predicted by models AH, A and H, and comparison by simplest model acceptance regions plot, and average final size among adults and children (bottom), measured in % of population, as predicted by models AH and A, for $R_0 = 1.5$ (left: a-d, m-p), $R_0 = 2$ (middle: e-h, q-t) and $R_0 = 4$ (right: i-l, u-x), as a function of p_{aa} and ψ, for the population of Great Britain. UK-like contact patterns are assumed: $\gamma = 0.75$, $\theta_g = 0.58$. Other parameters: $\phi = 1$ and $\varepsilon = 5\%$.

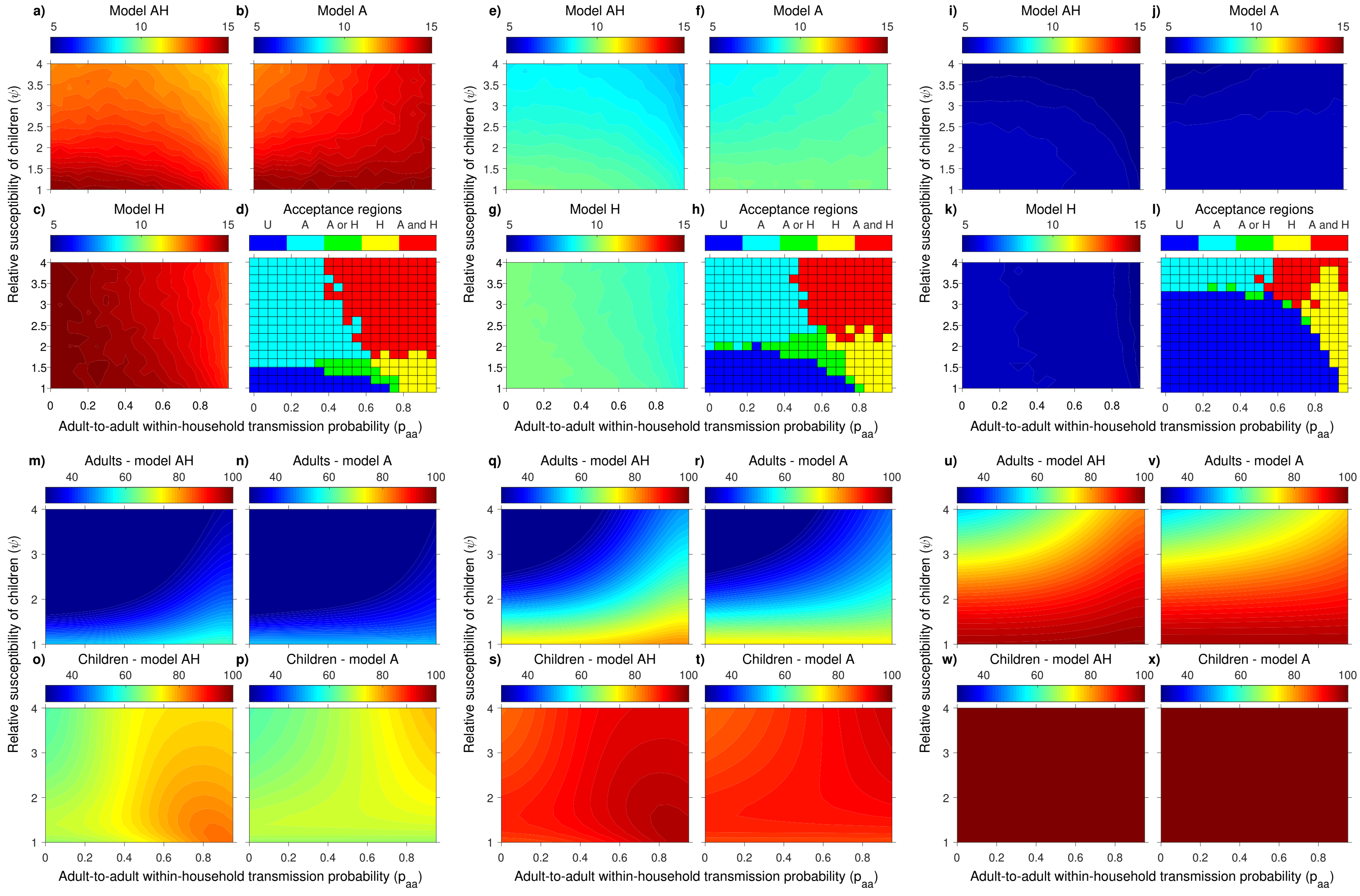

Supplementary Figure 14: Simplest model acceptance regions, based on each output separately and all three together, with UK-like contact patterns. The simplest model that accurately captures each output or all three simultaneously (intersection) is identified over the (p_{aa}, ψ)-space for $R_0 = 1.5$ (left: a-d, m-p), $R_0 = 2$ (middle: e-h, q-t) and $R_0 = 4$ (right: i-l, u-x) and for $\phi = 1$ (top: a-l) and $\phi = 2$ (bottom: m-x), for the population of Great Britain. UK-like contact patterns are assumed: $\gamma = 0.75$, $\theta_g = 0.58$. Other parameters: $\varepsilon = 5\%$.

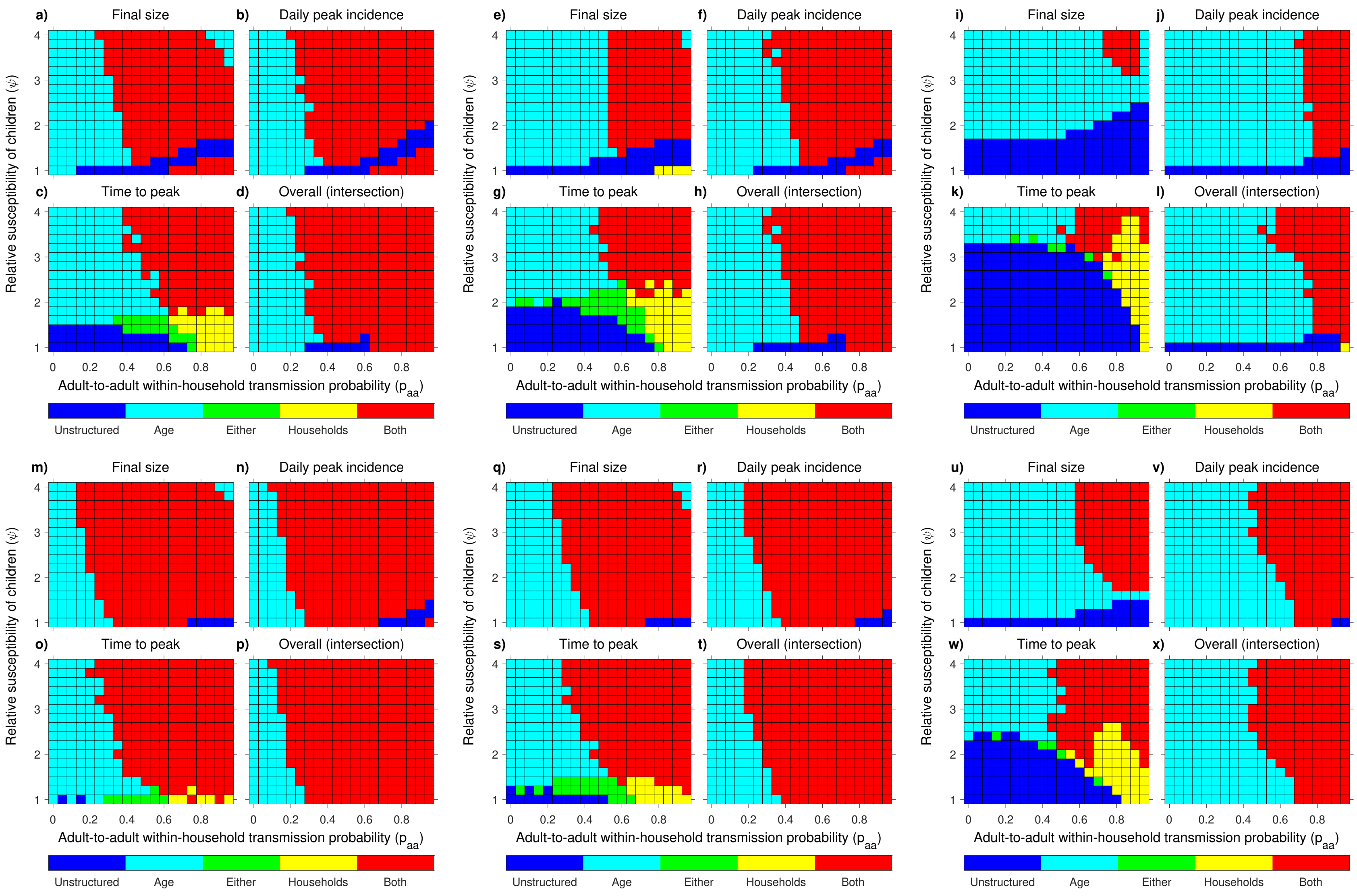

Supplementary Figure 15: Simplest model acceptance regions, based on each output separately and all three together, with UK-like contact patterns. The simplest model that accurately captures each output or all three simultaneously (intersection) is identified over the (p_{aa}, ψ)-space for $R_0 = 1.5$ (left: a-d, m-p), $R_0 = 2$ (middle: e-h, q-t) and $R_0 = 4$ (right: i-l, u-x) and for $\varepsilon = 1\%$ (top: a-l) and $\varepsilon = 10\%$ (bottom: m-x), for the population of Great Britain. UK-like contact patterns are assumed: $\gamma = 0.75$, $\theta_g = 0.58$. Other parameters: $\phi = 1$.

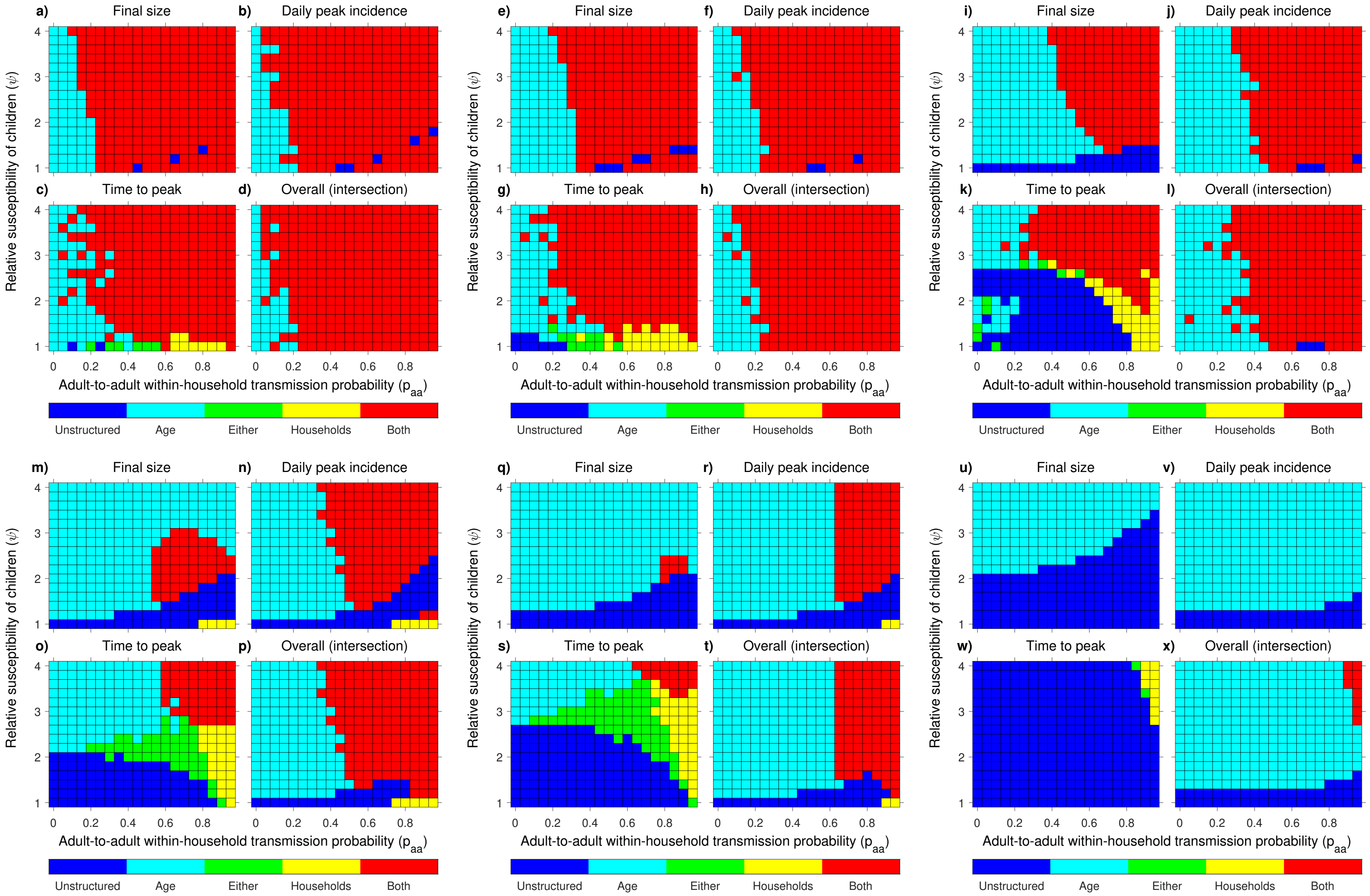

Supplementary Figure 16: Sensitivity analysis on overall simplest model acceptance regions with UK-like contact patterns. Overall simplest model acceptance regions and superimposed SAR contour levels are plotted for various values of R_0 and ϕ, with rejection thresholds $\varepsilon = 1\%$ (A), $\varepsilon = 5\%$ (B; same regions as in Figure 3B of main text) and $\varepsilon = 10\%$ (C), for the population of Great Britain. UK-like contact patterns are assumed: $\gamma = 0.75$, $\theta_g = 0.58$.

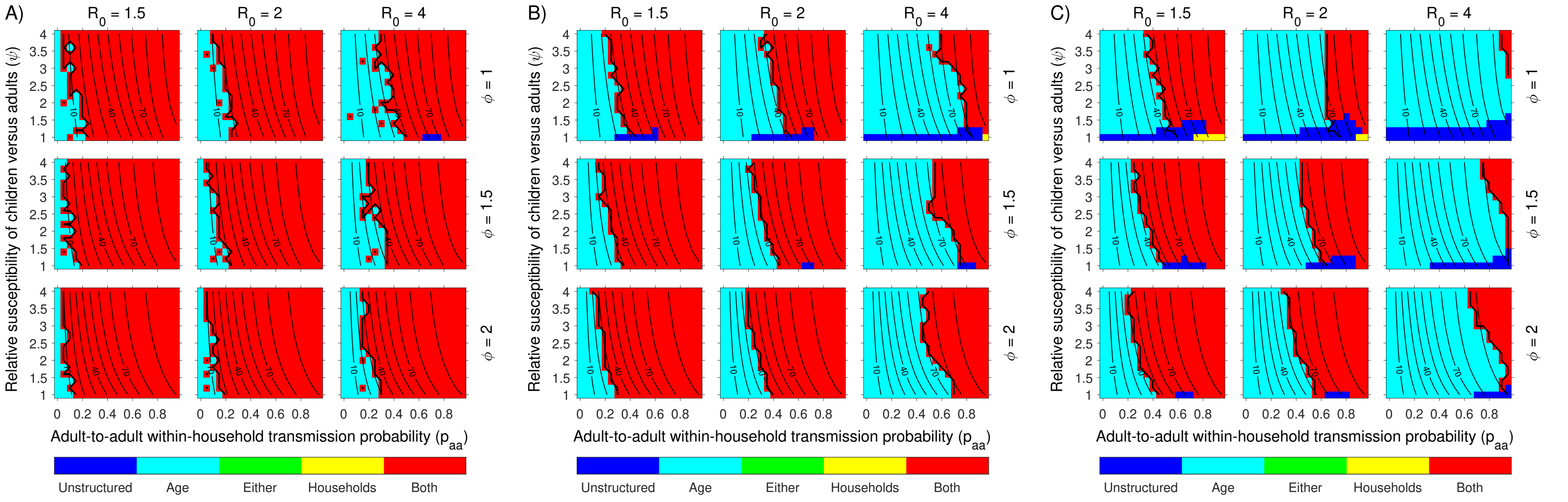

Supplementary Figure 17: Sensitivity analysis on overall simplest model acceptance regions with intermediate contact patterns. Overall simplest model acceptance regions and superimposed SAR contour levels are plotted for various values of R_0 and ϕ, with a rejection threshold $\varepsilon = 5\%$ and contact patterns intermediate between random ($\gamma = 1, \theta_g = 0.2273$) and UK-like ($\gamma = 0.75, \theta_g = 0.58$). Top: $\theta_g = 0.4$ (A: $\gamma = 1$; B: $\gamma = 0.75$). Bottom: $\theta_g = 0.5$ (C: $\gamma = 1$; D: $\gamma = 0.75$).

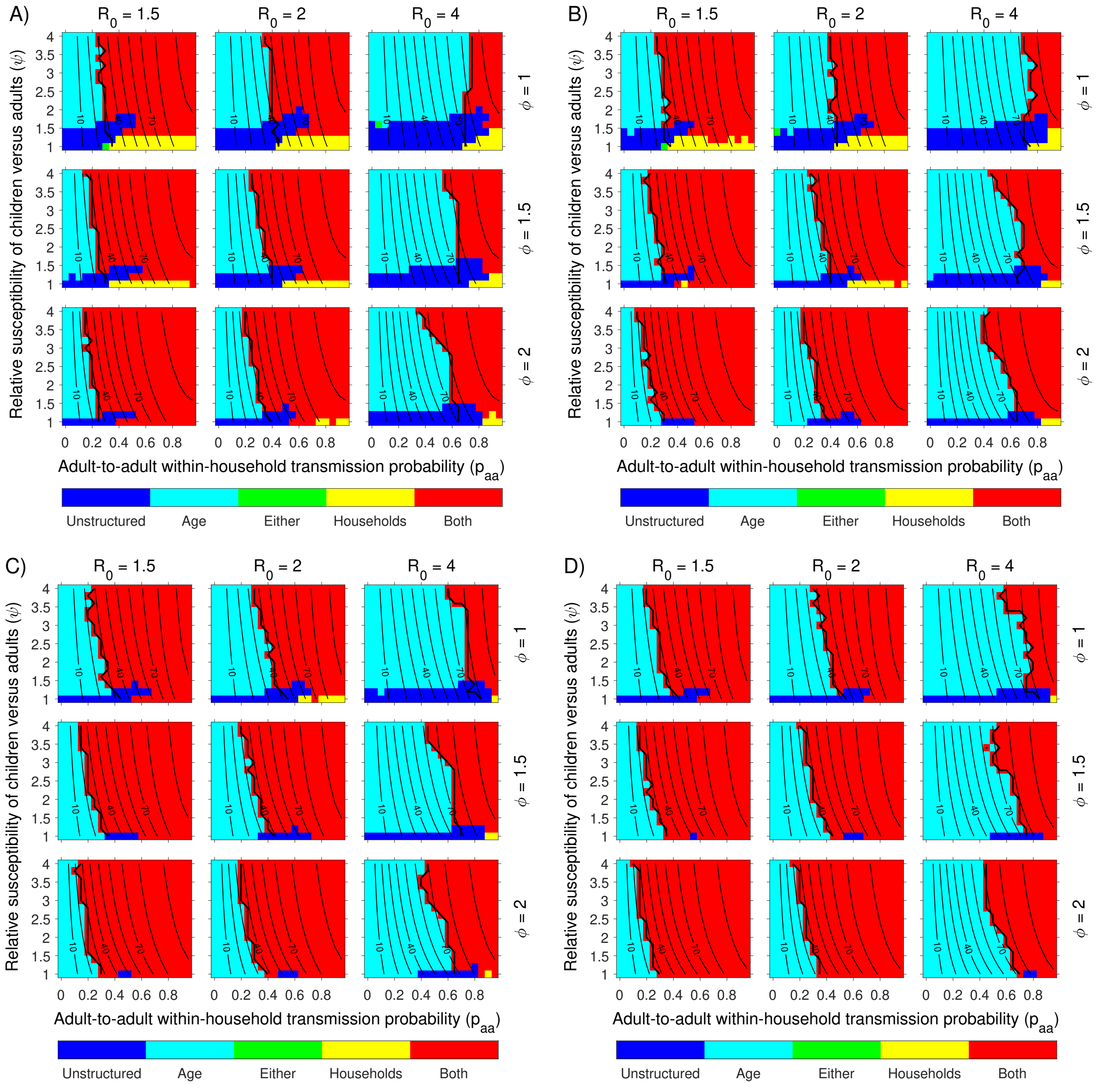

Supplementary Figure 18: Model mapping at constant real-time growth rate r. Overall simplest model acceptance regions are plotted after the model mapping procedure is performed by matching the same r across all models, for various values of r (0.14552, 0.25282 and 0.52588, respectively corresponding to $R_0 = 1.5$, 2 and 4 at $p_{aa} = 0$) and ϕ, for random mixing (top row: A-C) and UK-like contact patterns (bottom row: D-F), and $\varepsilon = 1\%$ (left: A and D), $\varepsilon = 5\%$ (middle: B and E) and $\varepsilon = 10\%$ (right: C and F).

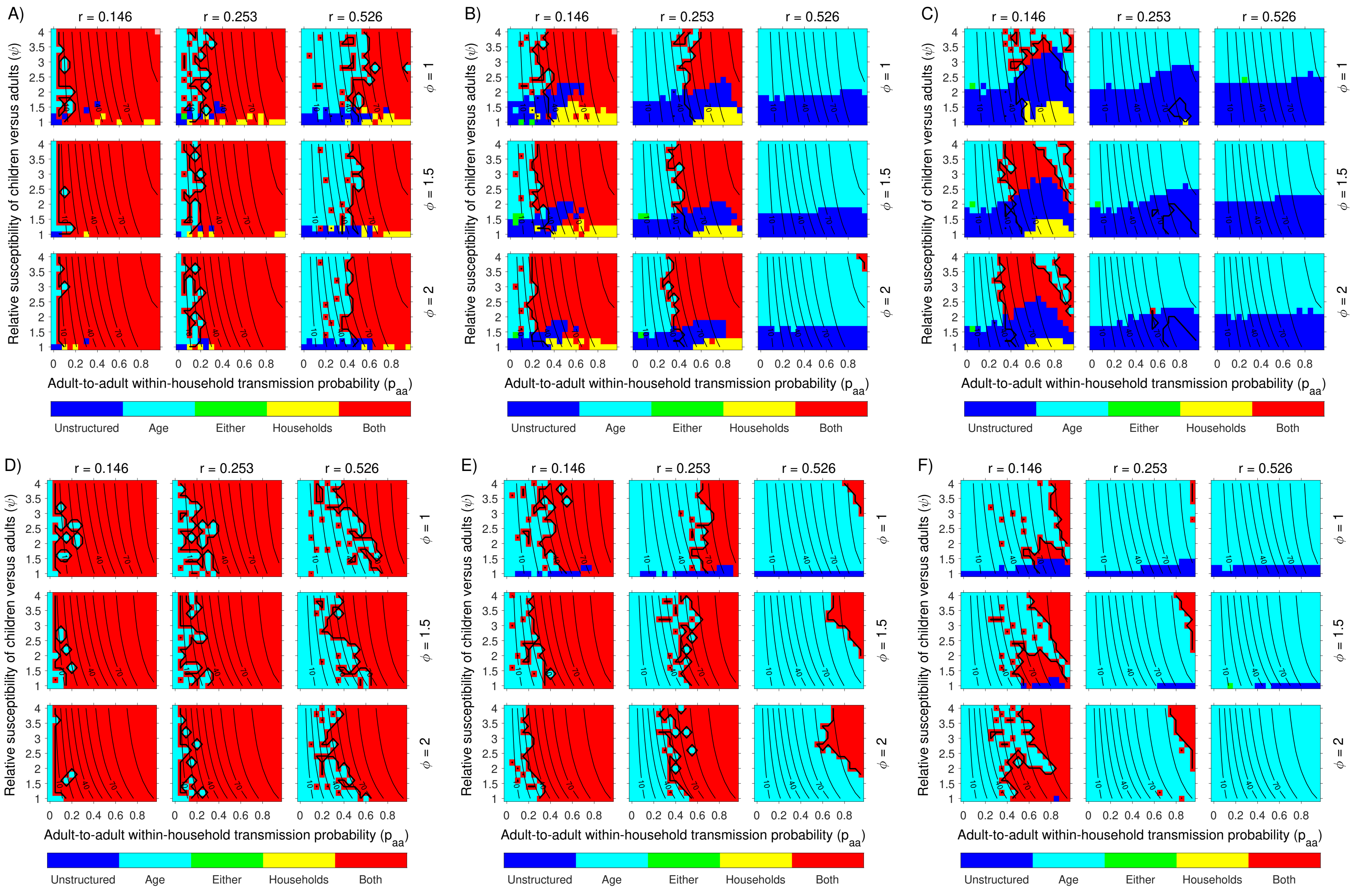

Supplementary Figure 19: Model mapping when children can also be less susceptible/infectious than adults. Overall simplest model acceptance regions are plotted for parameter values including $\phi < 1$ and/or $\psi < 1$, for random mixing (top: A-C) and UK-like contact patterns (bottom: D-F), and $\varepsilon = 1\%$ (left: A and D), $\varepsilon = 5\%$ (middle: B and E) and $\varepsilon = 10\%$ (right: C and F). Note that the y-axis is expressed in $\log_2 \psi$ and that ϕ takes values 1/2, 1 and 2. Note the broad lighter-shaded regions where no valid assortativity for model A can be found.

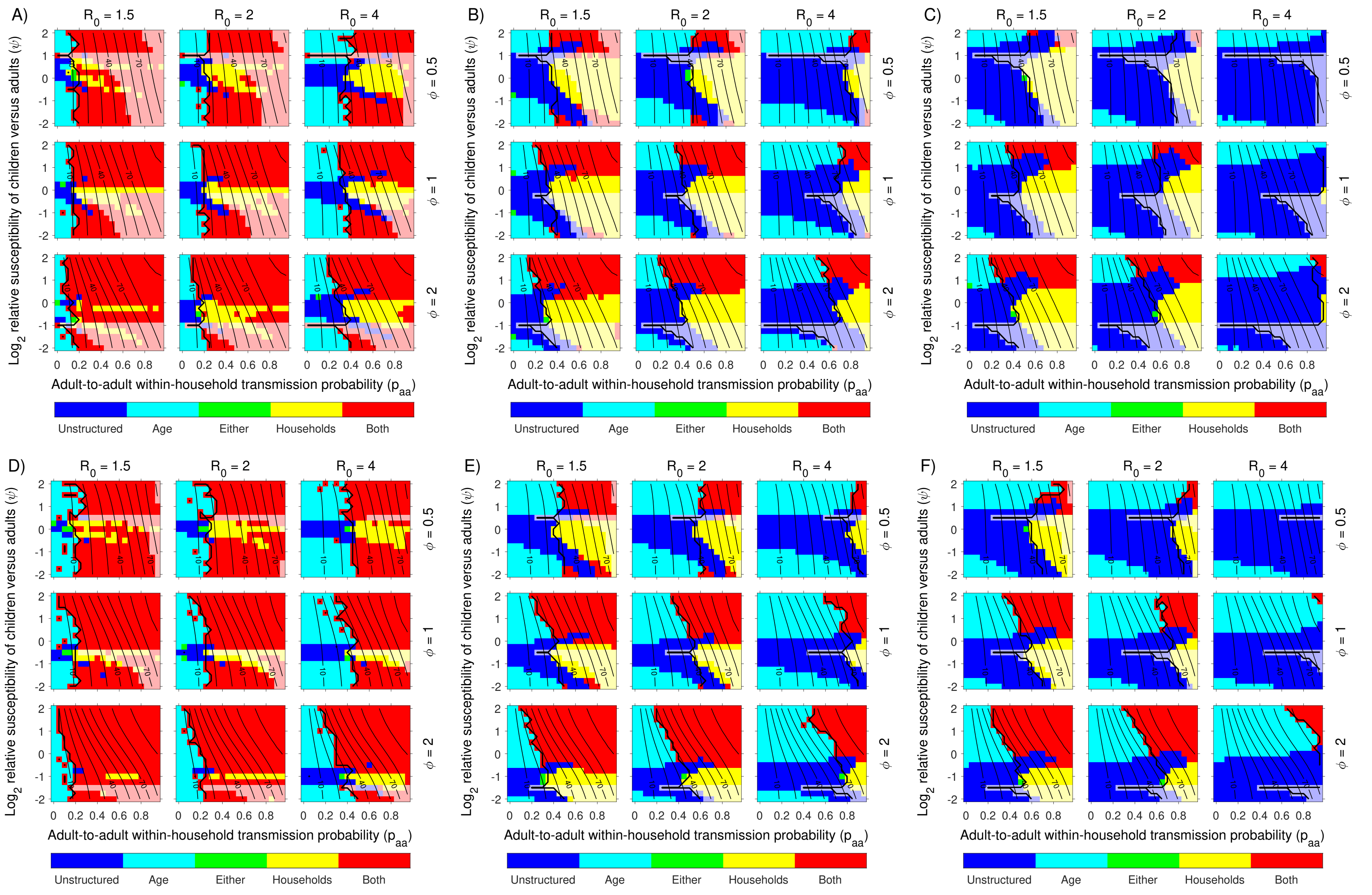

Supplementary Figure 20: Mapped assortativity of model A for Sierra Leone, with random mixing and UK-like contact patterns. Global assortativity θ^{A} estimated for model A from the model mapping procedure, with random mixing (A: $\gamma = 1$, $\theta_g = 0.5381$) and UK-like contact patterns including assortative mixing (B:, $\gamma = 0.75$, $\theta_g = 0.58$) as a function of p_{aa} and ψ for various values of R_0 and ϕ, for the population of Sierra Leone. The dashed line (also along the y-axis) shows where the assortativity has not changed when households are removed ($\theta^{\mathrm{A}} = \theta_g$). In the white region, no value of θ^{A} can be found to match the same age-stratified incidence as model AH (see Supplementary Discussion, Section 2.3.3). For the same reason, only the region $\psi > 1$ is plotted, as no value of θ^{A} can be found for $\psi = 1$ when random mixing is assumed (see Supplementary Methods, Section 1.3, and Supplementary Discussion, Section 2.3.3). The color scale is consistent with that of Supplementary Figure 10 to facilitate comparison (though note the reduced range of p_{aa}).

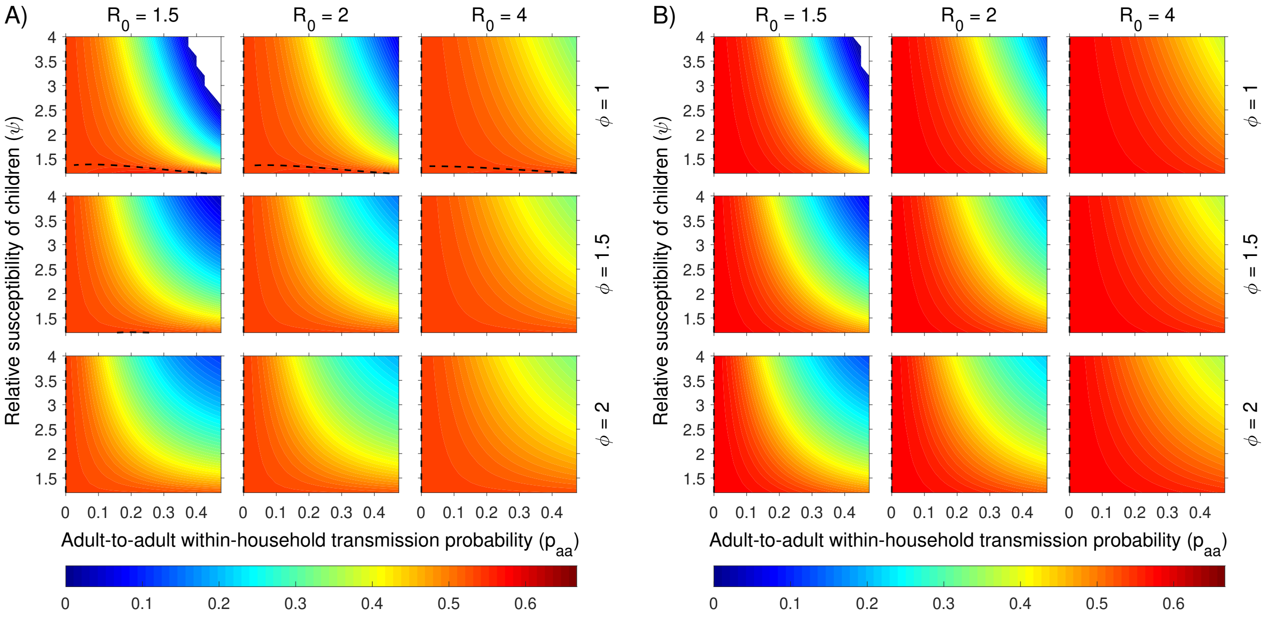

Supplementary Figure 21: Sensitivity analysis on overall simplest model acceptance regions for Sierra Leone. Overall simplest model acceptance regions and superimposed SAR contour levels are plotted with random mixing (A: $\gamma = 1$, $\theta_g = 0.5381$) and UK-like contact patterns (B: $\gamma = 0.75$, $\theta_g = 0.58$), for various values of R_0 and ϕ, a rejection threshold $\varepsilon = 5\%$, and the population of Sierra Leone.

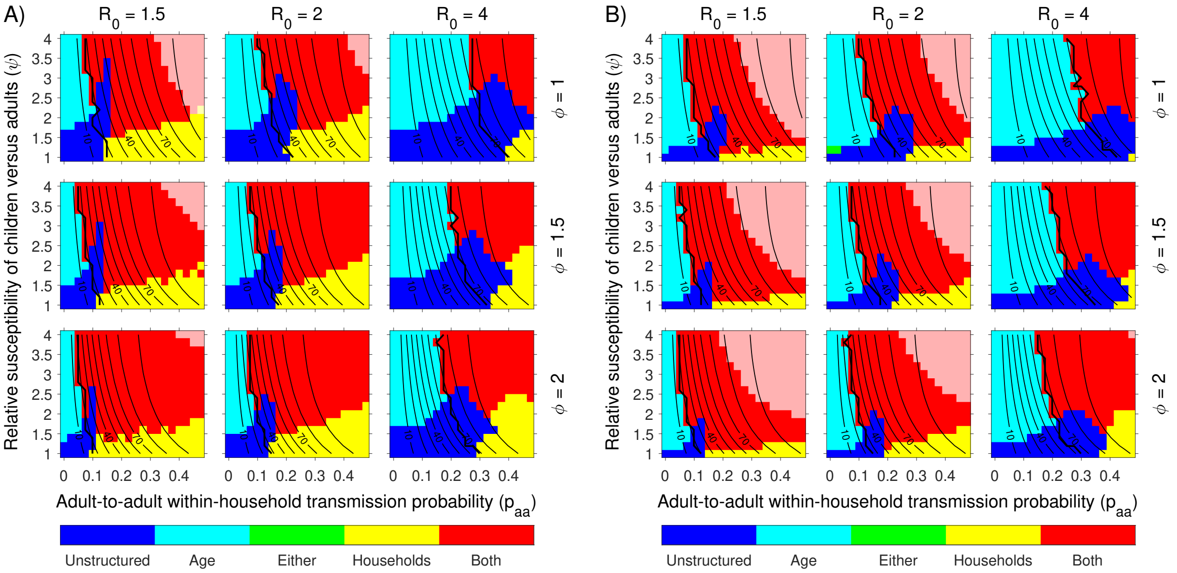

Supplementary Figure 22: Mapped assortativity of model A for South Africa, with random mixing and UK-like contact patterns. Global assortativity θ^{A} estimated for model A from the model mapping procedure, with random mixing (A: $\gamma = 1$, $\theta_g = 0.4592$) and UK-like contact patterns including assortative mixing (B:, $\gamma = 0.75$, $\theta_g = 0.58$) as a function of p_{aa} and ψ for various values of R_0 and ϕ, for the population of South Africa. The dashed line (also along the y-axis) shows where the assortativity has not changed when households are removed ($\theta^{\mathrm{A}} = \theta_g$). In the white region, no value of θ^{A} can be found to match the same age-stratified incidence as model AH (see Supplementary Discussion, Section 2.3.3). For the same reason, only the region $\psi > 1$ is plotted, as no value of θ^{A} can be found for $\psi = 1$ when random mixing is assumed (see Supplementary Methods, Section 1.3, and Supplementary Discussion, Section 2.3.3). The color scale is consistent with that of Supplementary Figure 10 to facilitate comparison (though note the reduced range of p_{aa}).

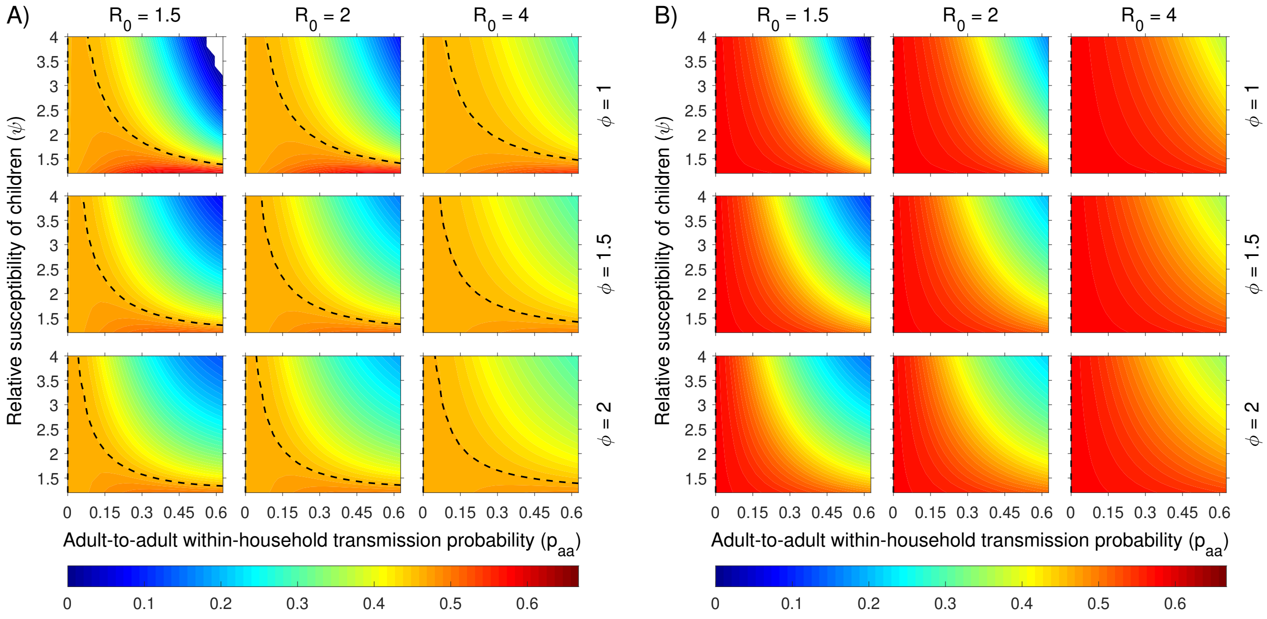

Supplementary Figure 23: Sensitivity analysis on overall simplest model acceptance regions for South Africa. Overall simplest model acceptance regions and superimposed SAR contour levels are plotted with random mixing (A: $\gamma = 1$, $\theta_g = 0.4592$) and UK-like contact patterns (B: $\gamma = 0.75$, $\theta_g = 0.58$), for various values of R_0 and ϕ, a rejection threshold $\varepsilon = 5\%$, and the population of South Africa.

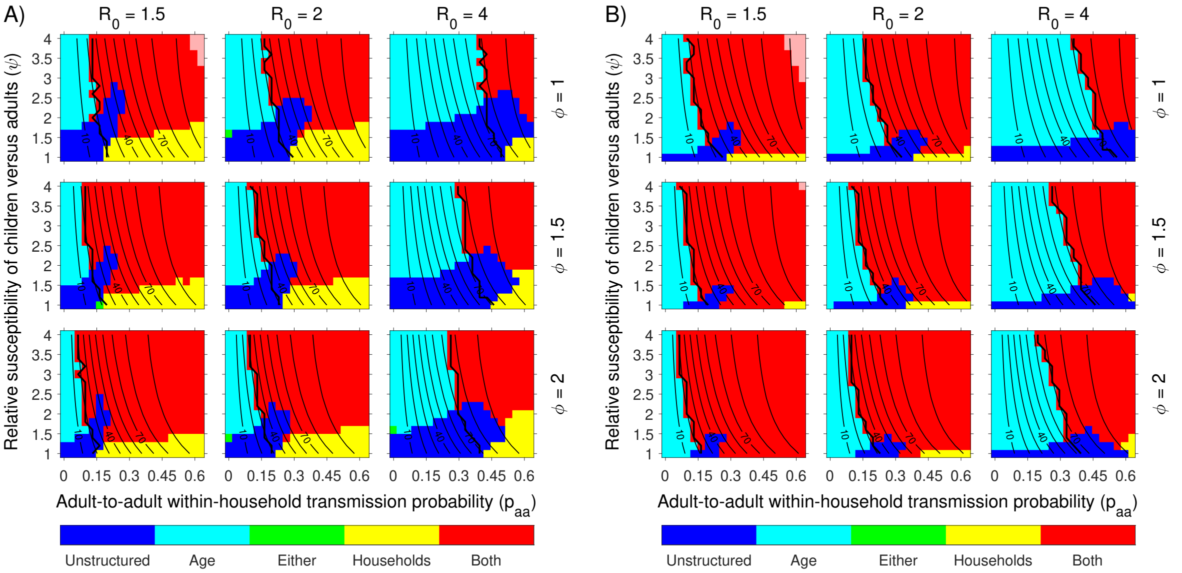

Supplementary Figure 24: Assessment of range of validity of linear fit for the rule of thumb. The table assesses the quality of fit of linear regression models through SAR data over multiple possible intervals of R_0 values from 1.1 to 4, to inform the formulation of the rule of thumb, for random mixing (A) and UK-like contact patterns (B), for the population of Great Britain and an accuracy threshold of 5%. Each sub-table refers to a different value of ϕ (each row) and range of values of ψ (each column). In each sub-table, the values of R_0 along the left-most column and the values of R_0 on the top row indicate, respectively, the start and end (inclusive) of the range of values of R_0 through which the linear regression is performed. At minimum, 3 consecutive values of R_0 are used. The coefficients for each fit are shown in each table cell (top: regression coefficient; bottom: intercept), and the adjusted R^2 value is shown by the cell colour.

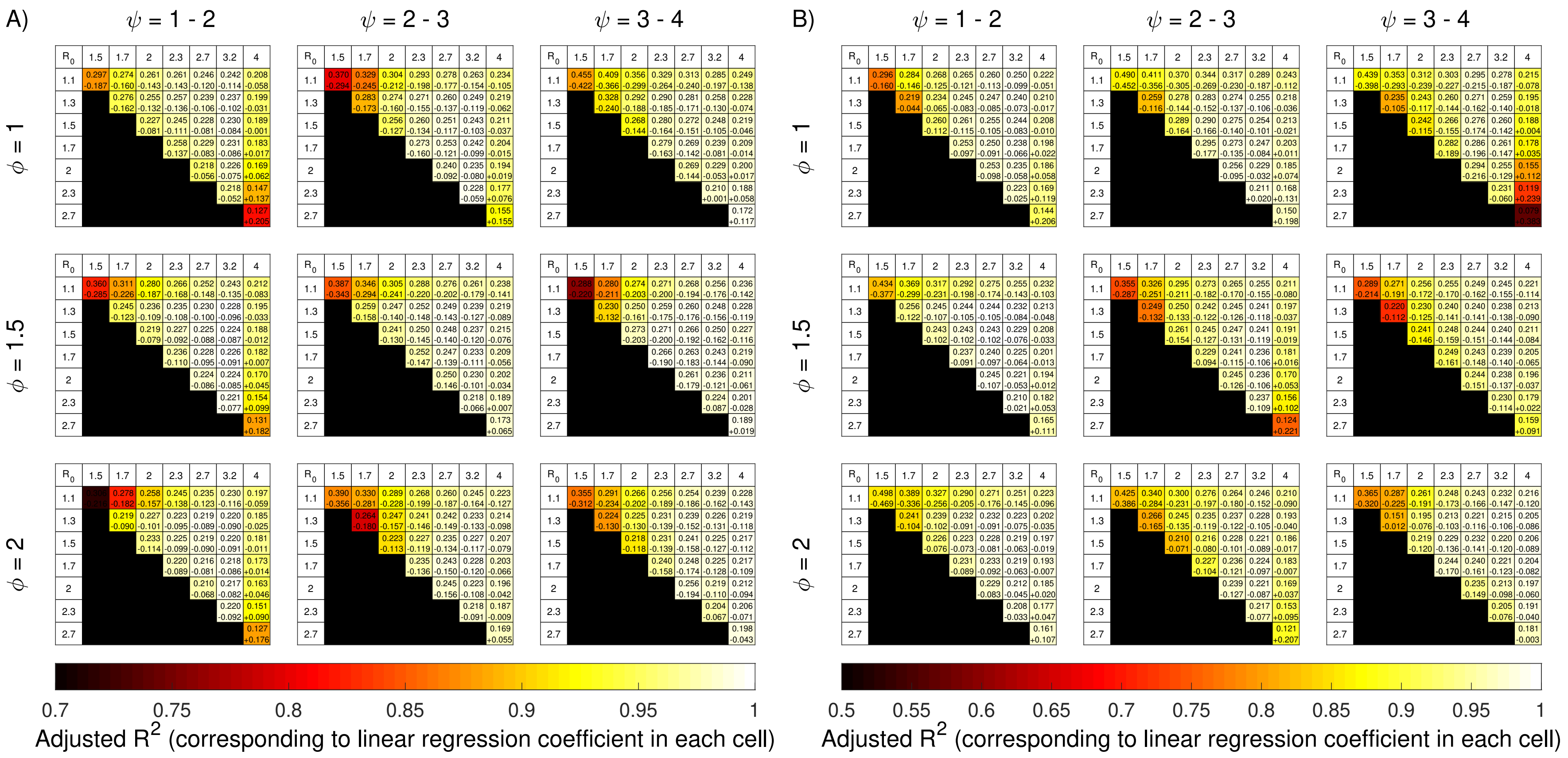

Supplementary Figure 25: Empirical rule of thumb for when the household structure is needed for accurate predictions. Each plot specifies the level of SAR above which a household structure is necessary for output prediction within a $\varepsilon = 1\%$ (A), $\varepsilon = 5\%$ (B) and $\varepsilon = 10\%$ (C) relative accuracy for Great Britain (left; fraction of children = 22.73%, mean household size = 2.35), South Africa (middle; fraction of children = 45.92%, mean household size = 4.27) and Sierra Leone (right; fraction of children = 53.81%, mean household size = 5.85), for random mixing and UK-like contact patterns (see Supplementary Discussion, Section 2.4 for details). In (D) the coefficients of the rule of thumb (regression coefficient β_1 and intercept β_0) are plotted against each other, for different populations and accuracy thresholds.

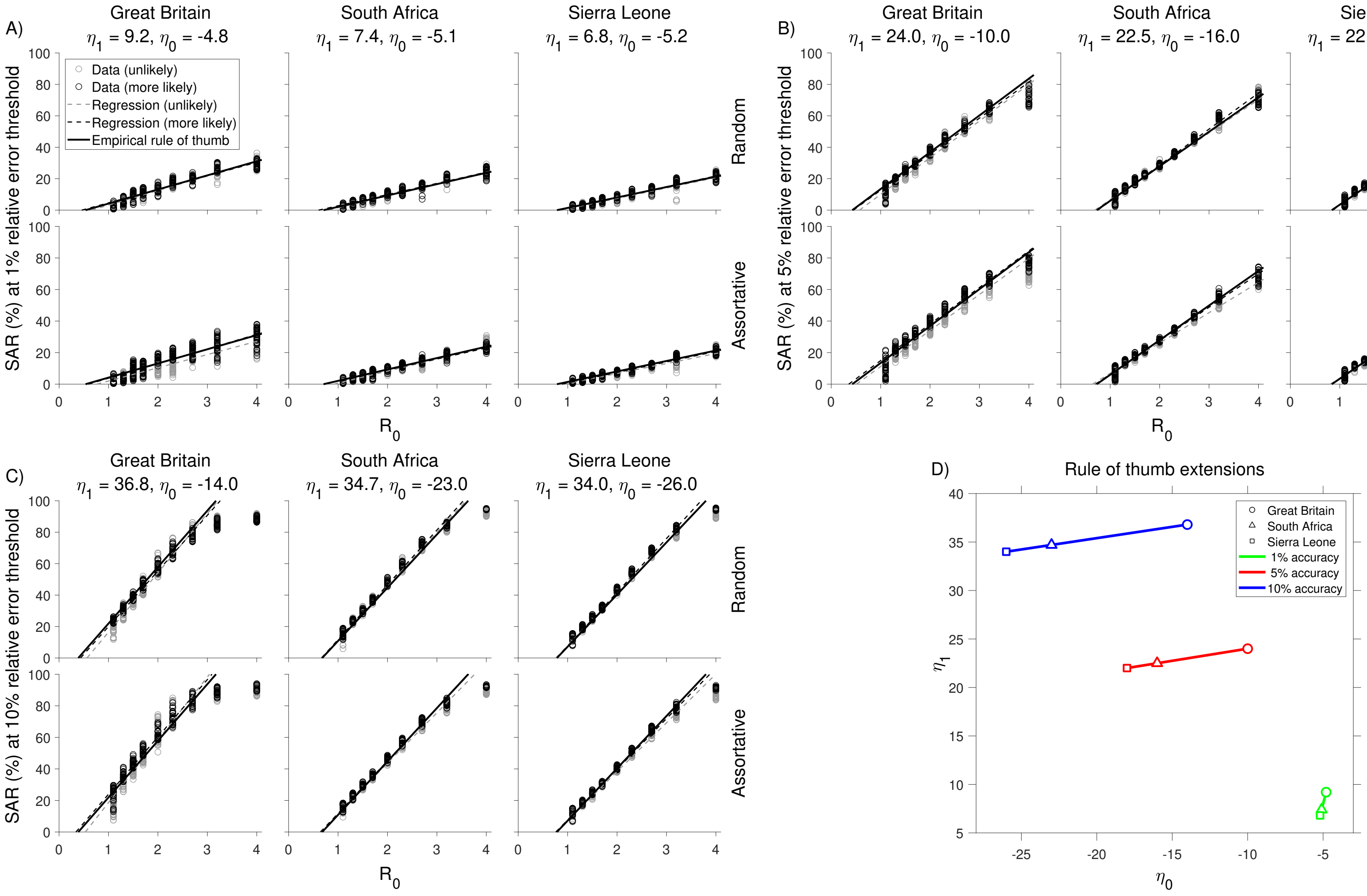

Supplementary References

C. L. Addy, I. M. Longini Jr, and M. Haber. A generalized stochastic model for the analysis of infectious disease final size data. *Biometrics*, 47(3):961–74, 1991.

M. Ajelli, B. Gonçalves, D. Balcan, V. Colizza, H. Hu, J. J. Ramasco, S. Merler, and A. Vespignani. Comparing large-scale computational approaches to epidemic modeling: agent-based versus structured metapopulation models. *BMC Infectious Diseases*, 10(1):1, 2010.

M. Ajelli, S. Merler, A. Pugliese, and C. Rizzo. Model predictions and evaluation of possible control strategies for the 2009 A/H1N1v influenza pandemic in Italy. *Epidemiology and Infection*, 139(1): 68–79, 2011.

H. Andersson and T. Britton. *Stochastic epidemic models and their statistical analysis.* Springer, 2000.

V. Andreasen. The final size of an epidemic and its relation to the basic reproduction number. *Bulletin of Mathematical Biology*, 2011.

F. Ball, T. Britton, and D. Sirl. Household epidemic models with varying infection response. *Journal of Mathematical Biology*, 63(2):309–337, 2011.

F. Ball, L. Pellis, and P. Trapman. Reproduction numbers for epidemic models with households and other social structures II: Comparisons and implications for vaccination. *Mathematical Biosciences*, 274:108–139, 2016.

F. G. Ball. A unified approach to the distribution of total final size and total area under the trajectory of infectives in epidemic models. *Advances in Applied Probability*, 18(2):289–310, 1986.

F. G. Ball and P. Neal. A general model for stochastic SIR epidemics with two levels of mixing. *Mathematical Biosciences*, 180(1-2):73–102, 2002.

S. Cauchemez, F. Carrat, C. Viboud, A.-J. J. Valleron, and P.-Y. Y. Boëlle. A Bayesian MCMC approach to study transmission of influenza: application to household longitudinal data. *Statistics in Medicine*, 23(22):3469–87, 2004.

S. Cauchemez, C. A. Donnelly, C. Reed, A. C. Ghani, C. Fraser, C. K. Kent, L. Finelli, and N. M. Ferguson. Household transmission of 2009 pandemic influenza A (H1N1) virus in the United States. *The New England Journal of Medicine*, 361(27):2619–27, 2009.

S. Cauchemez, A. Bhattarai, T. L. Marchbanks, R. P. Fagan, S. Ostroff, N. M. Ferguson, and D. L. Swerdlow. Role of social networks in shaping disease transmission during a community outbreak of 2009 H1N1 pandemic influenza. *Proceedings of the National Academy of Sciences of the United States of America*, 108(7):2825–2830, 2011.

H. Chen, Y. Wang, W. Liu, J. Zhang, B. Dong, X. Fan, M. D. de Jong, J. Farrar, S. Riley, G. J. D. Smith, and Y. Guan. Serologic survey of pandemic (H1N1) 2009 virus, Guangxi Province, China. *Emerging Infectious Diseases*, 15(11):1849–1850, 2009.

B. J. Cowling, K. H. Chan, V. J. Fang, L. L. H. Lau, H. C. So, R. O. P. Fung, E. S. K. Ma, A. S. K. Kwong, C.-W. Chan, W. W. S. Tsui, H.-Y. Ngai, D. W. S. Chu, P. W. Y. Lee, M.-C. Chiu, G. M. Leung, and J. S. M. Peiris. Comparative epidemiology of pandemic and seasonal influenza A in households. *The New England Journal of Medicine*, 362(23):2175–84, 2010.

Department of Health/South Africa and Macro International. 2002. South Africa Demographic and Health Survey 1998 [Dataset]. ZAPR31FL.SAV. Pretoria, South Africa: Department of Health/South Africa [Producer]. ICF [Distributor], 2002.

O. Diekmann, H. Heesterbeek, and T. Britton. *Mathematical tools for understanding infectious disease dynamics*. Princeton University Press, 2012.

N. M. Ferguson, D. a. T. Cummings, C. Fraser, J. C. Cajka, P. C. Cooley, and D. S. Burke. Strategies for mitigating an influenza pandemic. *Nature*, 442(7101):448–52, 2006.

C. Fraser. Estimating individual and household reproduction numbers in an emerging epidemic. *PLoS ONE*, 2(8):e758, 2007.

C. Fraser, C. A. Donnelly, S. Cauchemez, W. P. Hanage, M. D. Van Kerkhove, T. D. Hollingsworth, J. Griffin, R. F. Baggaley, H. E. Jenkins, E. J. Lyons, T. Jombart, W. R. Hinsley, N. C. Grassly, F. Balloux, A. C. Ghani, N. M. Ferguson, A. Rambaut, O. G. Pybus, H. Lopez-Gatell, C. M. Alpuche-Aranda, I. B. Chapela, E. P. Zavala, D. M. E. Guevara, F. Checchi, E. Garcia, S. Hugonnet, and C. Roth. Pandemic potential of a strain of influenza A (H1N1): early findings. *Science (New York, N.Y.)*, 324(5934):1557–61, 2009.

C. Fraser, D. a. T. Cummings, D. Klinkenberg, D. S. Burke, and N. M. Ferguson. Influenza transmission in households during the 1918 pandemic. *American Journal of Epidemiology*, 174(5):505–14, 2011.

K. Glass, G. N. Mercer, H. Nishiura, E. S. McBryde, and N. G. Becker. Estimating reproduction numbers for adults and children from case data. *Journal of the Royal Society, Interface / the Royal Society*, 8 (62):1248–59, 2011.

T. House and M. J. Keeling. Deterministic epidemic models with explicit household structure. *Mathematical Biosciences*, 213(1):29–39, 2008.

T. House, N. Inglis, J. V. Ross, F. Wilson, S. Suleman, O. Edeghere, G. Smith, B. Olowokure, and M. J. Keeling. Estimation of outbreak severity and transmissibility: Influenza A (H1N1) pdm09 in households. *BMC Medicine*, 10(1):117, 2012.

ICF. The DHS Program. Funded by USAID. https://dhsprogram.com/ [Accessed January, 22, 2020].

M. J. Keeling and P. Rohani. *Modeling infectious diseases in humans and animals*. Princeton University Press, 2008.

E. Kenah. Contact intervals, survival analysis of epidemic data, and estimation of R_0. *Biostatistics*, 12 (3):548–566, jul 2011.

E. Kenah, M. Lipsitch, and J. M. Robins. Generation interval contraction and epidemic data analysis. *Mathematical Biosciences*, 213(1):71–9, may 2008.

J. Miller. Epidemic size and probability in populations with heterogeneous infectivity and susceptibility. *Physical Review E*, 76(1):1–4, 2007.

J. Mossong, N. Hens, M. Jit, B. Philippe, A. Kari, R. Mikolajczyk, M. Massari, S. Salmaso, G. Scalia-Tomba, J. Wallinga, J. Heijne, M. Sadkowska-Todys, M. Rosinska, W. J. Edmunds, J. W. Edmunds, P. Beutels, K. Auranen, and G. S. Tomba. Social contacts and mixing patterns relevant to the spread of infectious diseases. *PLoS Medicine*, 5(3):e74, 2008.

Office for National Statistics. Table C0844, UK census, 2001. Available at: https://github.com/lorenzo-pellis/model-mapping.

L. Pellis, N. M. Ferguson, and C. Fraser. The relationship between real-time and discrete-generation models of epidemic spread. *Mathematical Biosciences*, 216(1):63–70, 2008.

L. Pellis, N. M. Ferguson, and C. Fraser. Epidemic growth rate and household reproduction number in communities of households, schools and workplaces. *Journal of Mathematical Biology*, 63(4):691–734, 2010.

L. Pellis, F. G. Ball, and P. Trapman. Reproduction numbers for epidemic models with households and other social structures. I. Definition and calculation of R0. *Mathematical Biosciences*, 235(1):85–97, 2012.

L. Pellis, S. E. F. Spencer, and T. House. Real-time growth rate for general stochastic SIR epidemics on unclustered networks. *Mathematical Biosciences*, 265:65–81, 2015.

G. Scalia-Tomba. Asymptotic final size distribution of the multitype reed–frost process. *Journal of Applied Probability*, 23(3):563–584, 1986.

R. E. H. Simpson. Infectiousness of communicable diseases in the household (measles, chickenpox, and mumps). *Lancet*, 2(6734):549–554, 1952.

Statistics Sierra Leone (SSL) and ICF Macro. 2009. Sierra Leone Demographic and Health Survey 2008 [Dataset]. SLPR51FL.SAV. Calverton, Maryland, USA: SSL and ICF Macro [Producers]. ICF [Distributor], 2009.

A. Svensson. A note on generation times in epidemic models. *Mathematical Biosciences*, 208(1):300–11, 2007.

P. Trapman, F. Ball, J.-S. Dhersin, V. C. Tran, J. Wallinga, and T. Britton. Inferring R_0 in emerging epidemics – the effect of common population structure is small. *Journal of the Royal Society Interface*, 13(121):20160288, 2016.

J. Wallinga and M. Lipsitch. How generation intervals shape the relationship between growth rates and reproductive numbers. *Proceedings of the Royal Society B: Biological Sciences*, 274(1609):599–604, 2007.
